# A Dynamic Self-Reporting Metal–Organic Framework System for Catalysis and Real-Time Reaction Monitoring

**DOI:** 10.1021/jacs.6c15370

**Published:** 2026-10-22

**Authors:** Jiatong Huo, Zongsu Han, Wenxuan Li, Yihao Yang, Zekihan Ozerdem, Narasimhan Loganathan, Xincheng Liu, Joshua Rushlow, Zhaoyi Liu, Angela K. Wilson, Kun-Yu Wang, Hong-Cai Zhou

**Affiliations:** † Department of Chemistry, 168556 Texas A&M University, College Station, Texas 77843, United States; ‡ Department of Chemistry, 6740 Princeton University, Princeton, New Jersey 08540, United States; § Department of Chemistry, Michigan State University, East Lansing, Michigan 44823, United States; ∥ Department of Chemistry, 6798 The University of Sheffield, Western Bank, Sheffield S10 2TN, United Kingdom; ⊥ Department of Chemistry, 14736 University of Miami, Coral Gables, Florida 33146, United States

## Abstract

Modern catalysis research has primarily focused on improving catalyst activity, selectivity, and stability through active-site engineering, microenvironment regulation, and mechanistic understanding. However, the sustainability and efficiency of catalytic processes also depend on the dynamic evolution of reaction systems, including substrate consumption, catalyst deactivation, and product accumulation. Despite their importance, these changes are typically assessed by off-line analysis rather than real-time monitoring, which hinders efficient process optimization and sustainable operation. Here, we develop a dynamic self-reporting (DSR) catalytic system based on Ag@UiO-66-(COOH)_2_/NDCA that simultaneously functions as a heterogeneous oxidation catalyst and an optical reporter for reaction progress. During oxidation, the emission intensity at 440 nm increases with product formation and exhibits a strong quantitative correlation with benzaldehyde yield, enabling reaction progress to be continuously tracked across a series of substituted benzyl alcohols. Mechanistic studies indicate that the luminescence response is governed by competitive absorption and substrate-dependent interactions. Density functional theory calculations further reveal that benzyl alcohol forms stronger dispersion-stabilized π···π interactions with the emissive NDCA^2–^ linkers than benzaldehyde, resulting in more pronounced luminescence quenching. As benzyl alcohol is converted into the more weakly interacting product, this quenching is progressively relieved, producing a measurable increase in emission intensity that reports the reaction progress. This integration provides a platform for reaction monitoring, process optimization, and future automated manufacturing.

## Graphical abstract

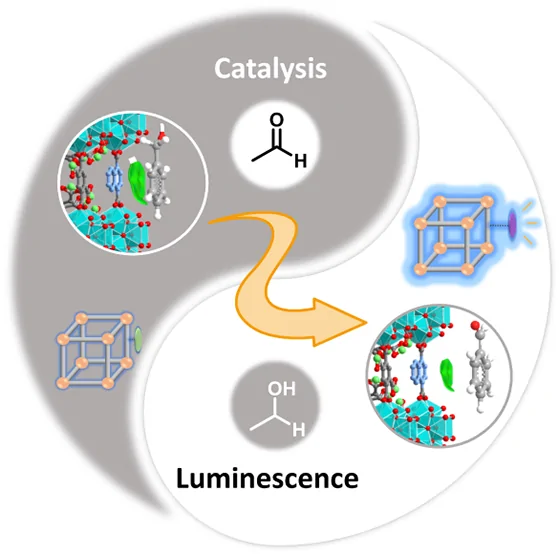

## Introduction

Across diverse fields of catalysis, catalyst design and catalytic performance have been extensively advanced through the optimization of active site structures,^1,2^ reaction microenvironments,^3,4^ and mechanistic pathways,^5^ improving production efficiency and supporting broader industrial and societal development. However, practical catalytic processes are also governed by multiple dynamic factors, including substrate concentration, reactant replenishment, catalyst addition, catalyst activation or regeneration, catalyst activity, and long-term stability.^6–8^ The efficiency and sustainability of industrial production therefore depend on real-time feedback on these operational factors, enabling timely process interventions, including reactant replenishment, product separation, catalyst regeneration or replacement, and solvent exchange, ultimately improving production efficiency, resource utilization, selectivity, and operational stability.^9–11^ Within the framework of process analytical technology, modern chemical manufacturing relies on in-line, online, or at-line measurements to provide continuous information on critical quality attributes and process variables.^12–15^ Such monitoring typically relies on external spectroscopic probes or chromatographic analysis based on sampling loops,^16–18^ requiring skilled operation, precise system control, well-maintained instrumentation, and a stable operating environment. Moreover, effective implementation requires rigorous integration between the analytical module and the catalytic reactor to ensure that the measured signals accurately reflect changes within the local catalytic microenvironment.^19,20^ In these systems, process information is obtained from external analytical components, while the catalyst itself primarily serves as a reaction promoter and provides limited information about its own operating state. This separation between catalysis and process monitoring limits adaptive process control and optimization. Therefore, developing catalytic systems capable of instant, sensitive, and visually readable self-reporting is of great importance for intelligent chemical manufacturing.

Oxidation reactions constitute an important class of catalysis and are widely used in industrial production and everyday life. Among them, the selective oxidation of benzyl alcohol to benzaldehyde is a representative model reaction owing to the industrial significance of benzaldehyde in pharmaceuticals, fragrances, dyes, and agrochemicals.^21–27^ Conventional analysis enables the quantification of conversion and selectivity after sampling, but it faces challenges in providing real-time visual readouts of reaction evolution during the catalytic process.^14,16,28^ Therefore, a catalytic system capable of simultaneously driving benzyl alcohol oxidation and reporting concentration changes of reactants or products would provide a useful model for integrating catalytic conversion with process monitoring.

Metal–organic frameworks (MOFs), porous crystalline materials constructed from metal nodes and organic linkers, possess high surface areas, ordered pore architectures, structural tunability, and programmable chemical environments.^29–32^ These features arise from the modular assembly of metal nodes and organic linkers, which allows pore size, topology, functionality, and local coordination environments to be precisely regulated.^33–35^ In catalysis, MOFs can provide confined reaction space and designable active sites that can promote selective molecular transformations and facilitate substrate-catalyst interactions.^36–38^ In luminescent recognition, emissive linkers, metal centers, or guest species can be incorporated to generate analyte-responsive optical signals through defined sites and pathways.^39,40^ However, current MOF-based catalysis research primarily focuses on optimizing catalytic activity, selectivity, and stability through active-site engineering and framework design, while luminescent sensing studies are mainly limited to static analyte detection under equilibrium conditions, where signals are generated only when target species exceed a threshold concentration. This one-to-one static sensing mode limits the ability to directly report the evolving state of dynamic processes such as catalytic reactions. Extending luminescent recognition from static detection to dynamic monitoring requires the signal to follow the continuous evolution of chemical composition and catalyst state during the reaction.

Herein, we develop a dynamic self-reporting (DSR) system that integrates catalytic conversion and optical feedback within a single MOF material, enabling the reaction to be monitored as it proceeds. Embedding this dynamic sensing behavior with catalytic activity in a single framework would yield “self-reporting” catalysts that couple catalytic transformation with *in situ* monitoring of reaction progress (Scheme 1). Based on this, an integrated catalysis-sensing system is designed and constructed, in which catalytically active and luminescence-responsive modules are spatially organized within a single MOF architecture, yielding a bifunctional material capable of simultaneously catalyzing the reaction and providing real-time visual readout of its progress. Using a dual-step pore engineering strategy, the luminescence-responsive center 1,4-naphthalenedicarboxylic acid (H_2_NDCA) and the catalytic center Ag­(I) were sequentially introduced into a robust Zr-MOF UiO-66-(COOH)_2_ (Figure 1). The resulting material Ag@UiO-66-(COOH)_2_/NDCA thus functions as both a heterogeneous oxidation catalyst and an *in situ* luminescent response unit, establishing a high-performance DSR system to track reaction progress.

**Scheme 1:**
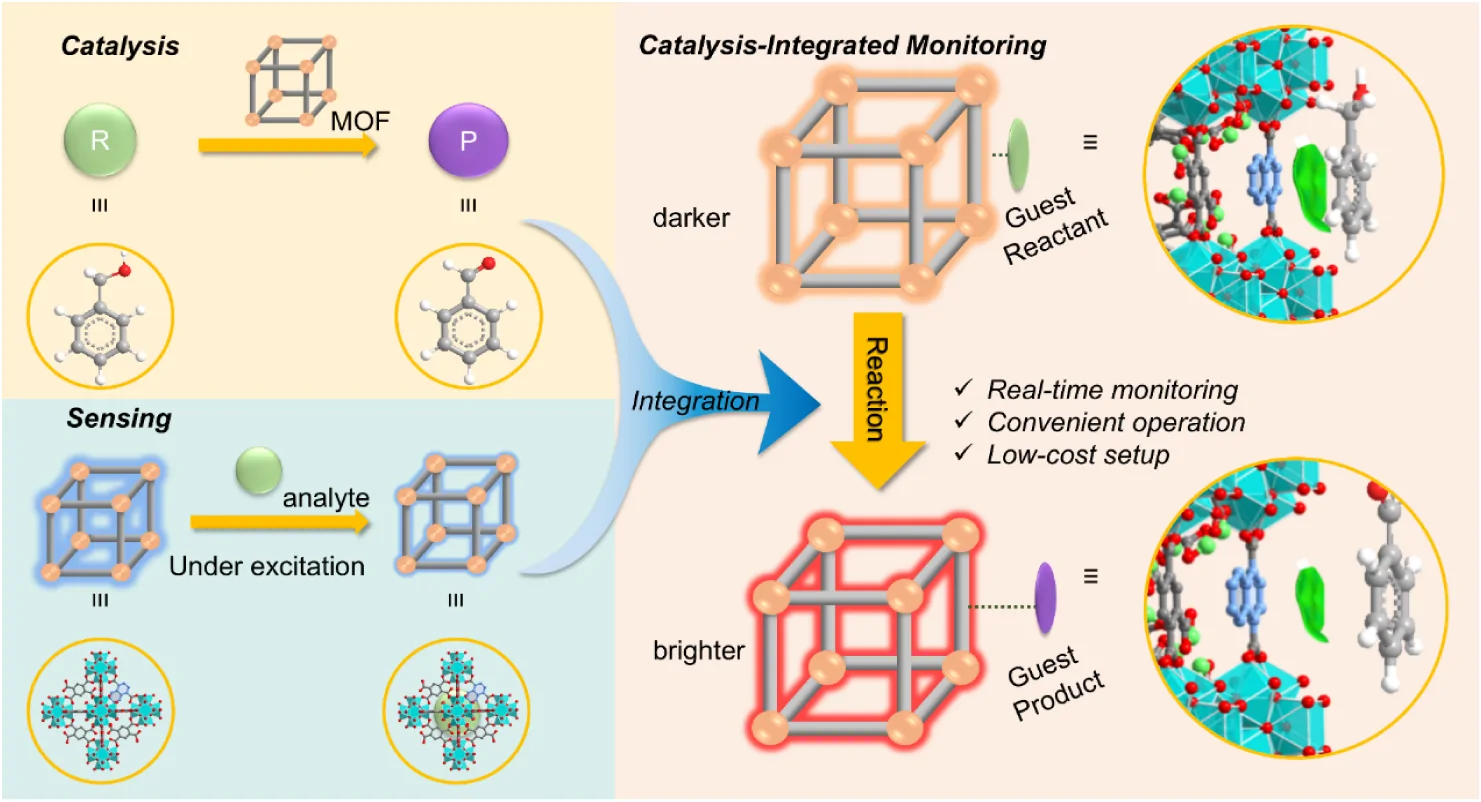
Integration of Catalytic and Luminescent Functions in a Single System to Enable *In Situ* Optical Monitoring of Reaction Progress

**Figure 1:**
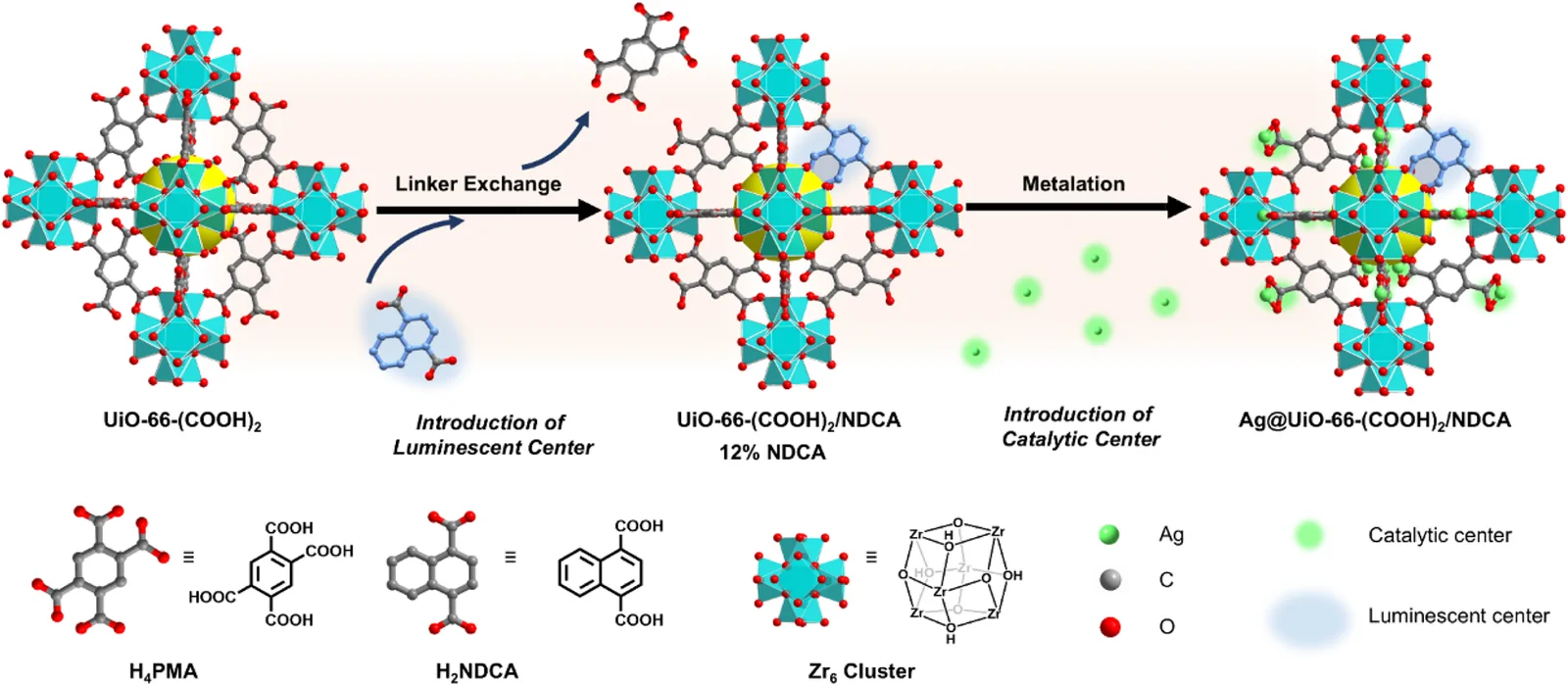
Design route for the dual-functional luminescent and catalytic system Ag@UiO-66-(COOH)_2_/NDCA. Yellow spheres represent the internal pore cavity of the MOFs.

## Results and Discussion

### Structures and Characterization

A dual-step pore engineering strategy was employed in which a luminescent linker was first introduced into a stable Zr-MOF by linker exchange, followed by incorporation of catalytically active metal centers (Figure 1). UiO-66-(COOH)_2_ was selected because of its excellent chemical and thermal stability^41–43^ and the presence of two additional, initially uncoordinated carboxylic acid groups per linker that offer potential coordination sites.^42^ The luminescent linker H_2_NDCA was successfully installed by partial linker exchange to serve as the luminescent center, and Ag­(I) was then introduced as the catalytic center via postsynthetic metalation of the remaining carboxylate sites. Ag­(I) was selected due to its high catalytic activity in alcohol oxidation and its limited light absorption capacity.^44^ These steps integrate catalytic and luminescent functionalities within a single MOF, enabling simultaneous catalysis and *in situ* optical monitoring.

Partial linker exchange of UiO-66-(COOH)_2_ with H_2_NDCA affords UiO-66-(COOH)_2_/NDCA. The emission spectrum of H_2_NDCA-exchanged material confirms the effective incorporation of the luminescent linker (Figure S1). ^1^H nuclear magnetic resonance (NMR) of UiO-66-(COOH)_2_/NDCA shows characteristic chemical shifts from both pyromellitic acid (H_4_PMA) and H_2_NDCA (Figure S2), and the integration of these signals gives an H_4_PMA:H_2_NDCA molar ratio of 7.26:1.00, corresponding to replacement of ∼12% of H_4_PMA by H_2_NDCA. The incorporation of H_2_NDCA represents a balance between maintaining luminescence intensity and providing sufficient Ag^+^ loading sites. Subsequent postsynthetic metalation with Ag­(I) yields Ag@UiO-66-(COOH)_2_/NDCA. Inductively coupled plasma mass spectrometry (ICP-MS) confirms the successful incorporation of Ag and reveals a Zr:Ag molar ratio of 6.94:1.00 (Figure S3), corresponding to approximately one Ag^+^ ion per 3.05 H_4_PMA linkers.

Powder X-ray diffraction (PXRD) patterns of UiO-66-(COOH)_2_, UiO-66-(COOH)_2_/NDCA and Ag@UiO-66-(COOH)_2_/NDCA all matched well with the simulated data (Figures S4–S6), indicating that the MOF was preserved throughout the modification process. As shown in Figure S7, infrared (IR) spectra of H_4_PMA and H_2_NDCA linkers display a characteristic carboxylic C=O stretching vibration at approximately 1670 cm^–1^.^45^ After linker exchange and metalation, this absorption band in UiO-66-(COOH)_2_/NDCA and Ag@UiO-66-(COOH)_2_/NDCA becomes markedly weaker, consistent with deprotonation and coordination of most carboxyl groups to the metal nodes, while extra free −COOH groups remain. Coordination to the Zr_6_ clusters shifts the C=O vibration from ∼1670 cm^–1^ to around 1573 cm^–1^, corresponding to the asymmetric stretching of coordinated carboxylate groups,^46^ and in Ag@UiO-66-(COOH)_2_/NDCA, the band at 1543 cm^–1^ is attributed to Ag-carboxylate coordination, reflecting further perturbation of the carboxylate stretching by Ag–O bonding.^47^ Scanning electron microscopy (SEM) images show the particle morphology and size of Ag@UiO-66-(COOH)_2_/NDCA (Figures S8–S10), while energy-dispersive X-ray spectroscopy (EDS) mapping confirms a uniform distribution of elements throughout the framework (Figures S11 and S12). Thermogravimetric analysis (TGA) of Ag@UiO-66-(COOH)_2_/NDCA reveals no obvious weight loss before 170 °C (Figure S13). In addition, single-crystal X-ray diffraction (SCXRD) indicates that Ag^+^ can coordinate to the carboxyl groups in a chelating mode (Table S1).

### Reaction Monitoring

The excitation and emission spectra of UiO-66-(COOH)_2_, UiO-66-(COOH)_2_/NDCA, and Ag@UiO-66-(COOH)_2_/NDCA confirm the contribution of the H_2_NDCA linker to the luminescence of the framework (Figures S14–S16). Time-dependent emission spectra of Ag@UiO-66-(COOH)_2_/NDCA confirm its optical stability at both room temperature and reaction temperature (Figures S17 and S18). Its dual-functional performance in the oxidation of benzyl alcohol was then evaluated using Na_2_S_2_O_8_ as the oxidant at 60 °C (Figure 2a). During the reaction, time-resolved emission spectra were recorded (Figure 2b), and ^1^H NMR spectra were tested to quantify benzaldehyde yield at various time points. The emission intensity at 440 nm increases rapidly within the first 30 min and then approaches a plateau, indicating fast initial conversion followed by gradual saturation (Figure 2c). The luminescence-time profile can be well described by an exponential function and closely matches the evolution of product yield (Figure 2c and Table S2), with replicate experiments showing highly reproducible traces (Figure S19).

**Figure 2:**
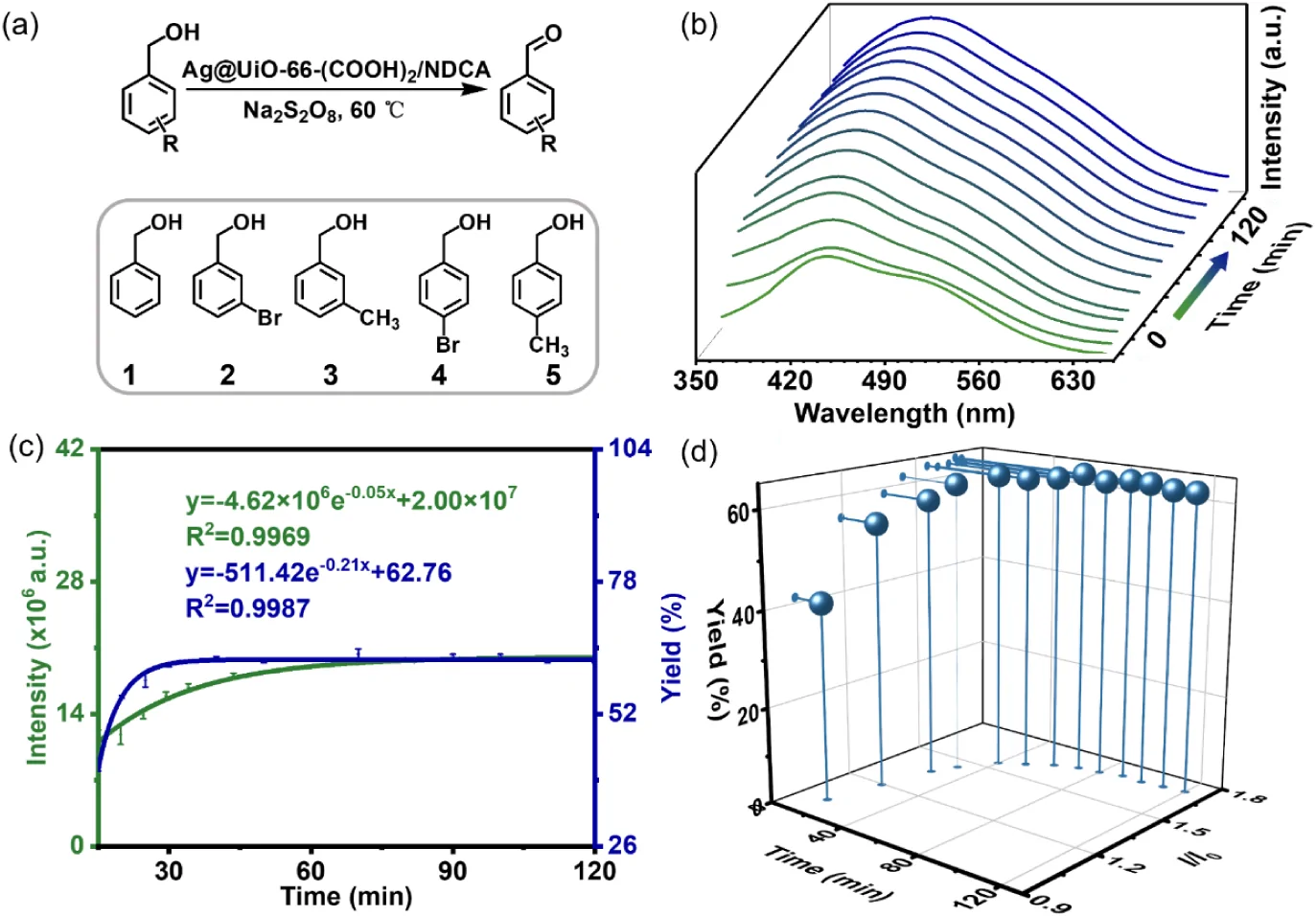
Real-time luminescence monitoring of benzyl alcohol oxidation using Ag@UiO-66-(COOH)_2_/NDCA. (a) Schematic of benzyl alcohol oxidation catalyzed by Ag@UiO-66-(COOH)_2_/NDCA. (b) Time-resolved emission spectra recorded during reaction **1**. (c) Emission intensity at 440 nm and benzaldehyde yield as a function of reaction time during reaction **1**. (d) Three-dimensional plot showing the relationship between yield, emission intensity changes, and reaction time for reaction **1**.

Moreover, plotting the emission intensity at 440 nm against benzaldehyde yield gives an exponential calibration curve with a high correlation coefficient (*R* ^2^ > 0.99) (Figure S20), with the luminescence signal increasing progressively as the reaction progressed (Figure 2d). These results collectively demonstrate that luminescence intensity changes in this system serve as robust optical proxies for monitoring the reaction process. As shown in Figure S21, PXRD patterns recorded before and after reactions remain unchanged, indicating that the framework structure was well preserved throughout the reaction. Furthermore, the concentration of Ag^+^ bound to the MOF remains constant, confirming that no significant Ag^+^ leaching occurred during the reaction process. The observed catalytic activity can therefore be attributed to Ag^+^ sites retained within the MOF pores rather than to homogeneous Ag^+^ species dispersed in the solution phase.

To evaluate the generality of Ag@UiO-66-(COOH)_2_/NDCA for this category of reactions, the oxidation of structurally related benzyl alcohols was tested under identical catalytic conditions (Tables S3–S6). Substrates bearing methyl or bromo groups at the meta- or para-positions were selected to evaluate the generality of this approach. As shown in Figure 3, all substituted benzyl alcohols display similar luminescence evolution. The emission intensity at 440 nm rises rapidly at an early stage and then approaches a plateau, indicating efficient catalytic conversion across the substrate set. Time-dependent luminescence traces can be well fitted by exponential functions, with R^2^ > 0.96, and the corresponding product yields exhibit nearly identical exponential growth within the first 30 min followed by steady-state behavior. Plotting emission intensity against product yield for each substrate (Figures S22–S25) gives exponential calibration curves with good correlation, demonstrating that the luminescence response quantitatively reports reaction progress irrespective of the electronic nature or position of the substituent. This robustness underscores the general applicability of Ag@UiO-66-(COOH)_2_/NDCA as a self-reporting catalytic system for simultaneous benzylic oxidation catalysis and real-time reaction monitoring.

**Figure 3:**
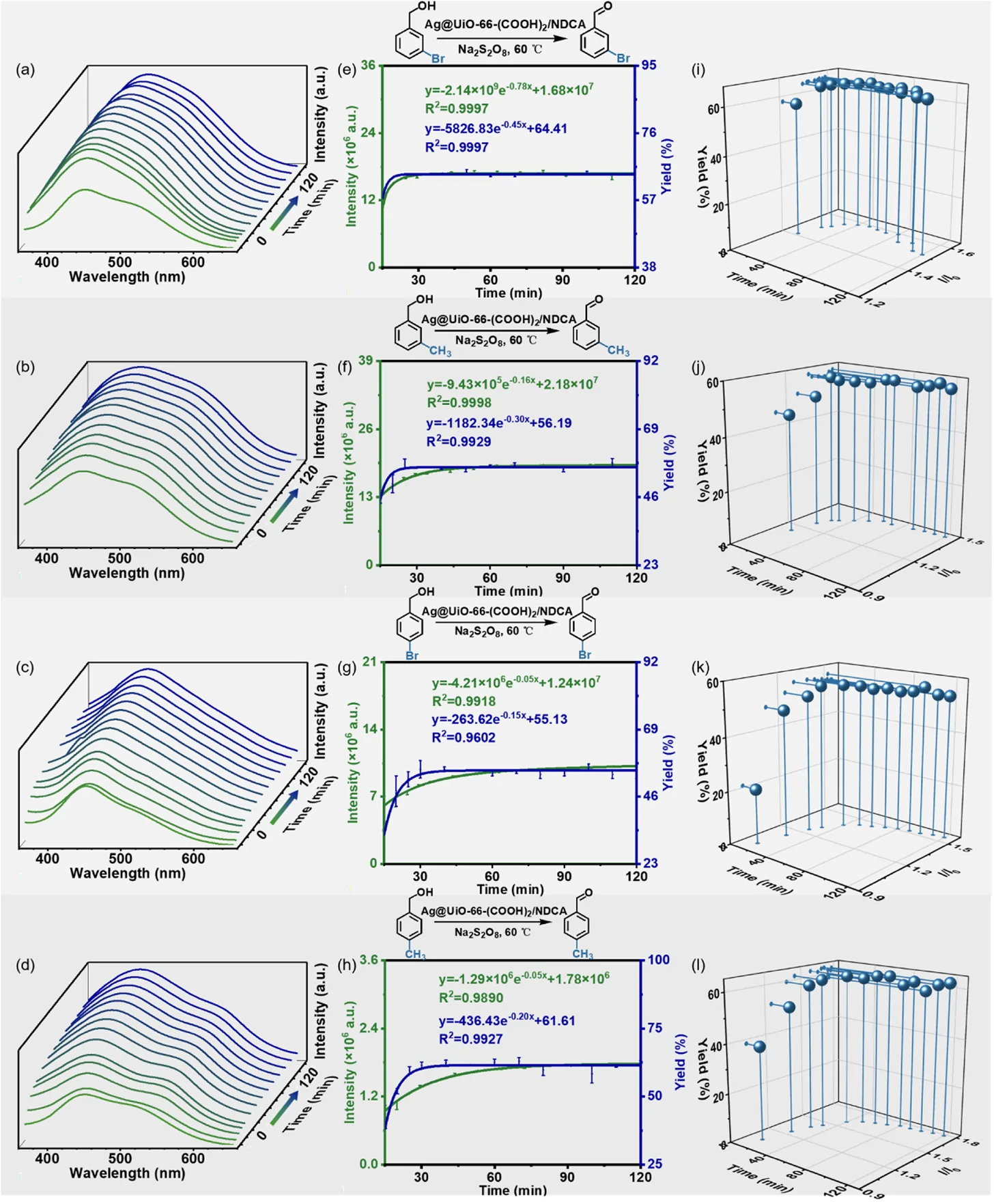
Real-time luminescence monitoring of substituted benzyl alcohol oxidation using Ag@UiO-66-(COOH)_2_/NDCA. Time-resolved emission spectra recorded during reaction (a) **2**, (b) **3**, (c) **4**, and (d) **5**. Emission intensity at 440 nm and the corresponding aldehyde yield as a function of reaction time during reaction (e) **2**, (f) **3**, (g) **4**, and (h) **5**. Three-dimensional plot showing the relationship between yield, emission intensity changes, and reaction time for reaction (i) **2**, (j) **3**, (k) **4**, and (l) **5**.

### Mechanistic Analysis

The luminescence response mechanisms toward individual reactant or product were investigated through a series of fundamental characterizations. Comparison of the ultraviolet–visible (UV–vis) spectra of the reactant, product, and MOF reveals significant spectral overlap, consistent with a competitive absorption effect, reducing the amount absorbed by the MOF and thus decreasing its luminescence intensity (Figures S26–S30). There is no obvious overlap between the emission spectrum of the MOF and the UV–vis absorption spectra of the substrates, indicating that Förster resonance energy transfer (FRET) is unlikely to exist in this system (Figures S31–S35). Frontier-orbital calculations show that the lowest unoccupied molecular orbital (LUMO) levels of the substrates possess higher energy levels than those of the MOF linkers, indicating that photoinduced electron transfer (PET) is thermodynamically unfavorable (Figure S36).

To further elucidate the mechanism underlying the dynamic luminescence intensity changes during the reaction process, a more detailed mechanistic investigation was carried out. Density functional theory (DFT) calculations were performed on model complexes of the NDCA^2–^ linker with benzyl alcohol and benzaldehyde (Figure 4 and Figures S37–S47). The results indicate that the reactant benzyl alcohol forms stronger dispersion-stabilized π···π interactions with the NDCA^2–^ units than the oxidized product. These results support a mechanism in which the MOF luminescence is quenched at the beginning of the reaction by strong π···π interactions between the emissive linkers and the benzyl alcohol molecules (Figure 4a and b). As the reaction proceeds and benzyl alcohol is converted into benzaldehyde, these interactions become weakened, leading to reduced quenching and recovery of the emission, which manifests as the measurable increase in luminescence intensity.

**Figure 4:**
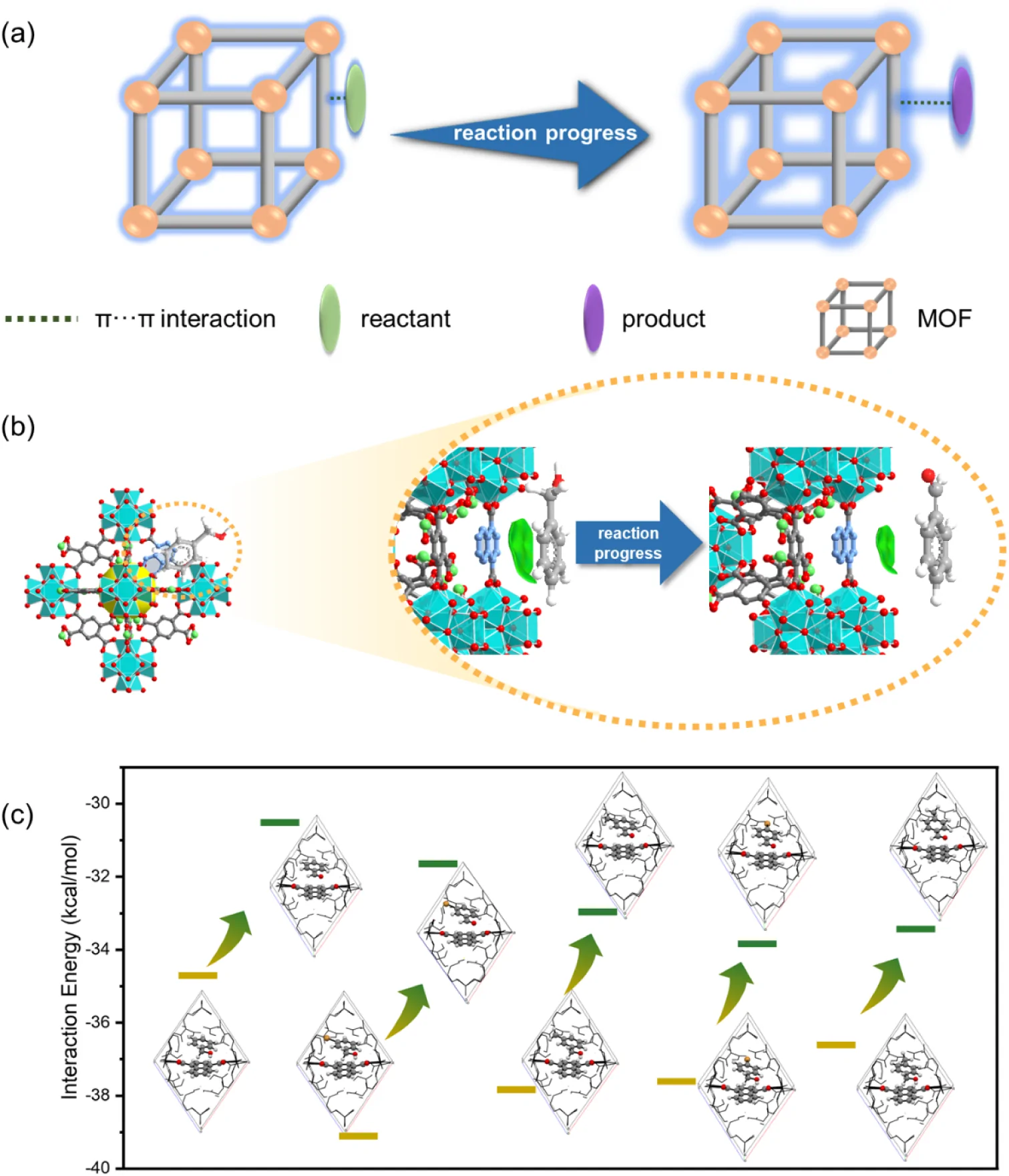
Illustration of the sensing mechanism based on DFT calculations and experimental results. (a) Scheme of the response mechanism. (b) Structural model of the reactants and products docked in the MOF. (c) Dispersion interaction between the unit cell with reactants and products. Atom codes: Zr, cyan; O, red; C, gray; H, white; Br, yellow; Ag, green.

Plane-wave (PW) DFT calculations were further performed on the periodic UiO-66-(COOH)_2_/NDCA with linkers to analyze noncovalent interactions between the atoms of the MOF and the reactants/products. The energetic and electronic structures obtained from PW-DFT calculations indicate that π···π interactions between NDCA^2–^ and the reactant benzyl alcohol are stronger than those with the benzaldehyde product for all the substrates examined (Figure 4c). The reported interaction energies validate the outcomes from the cluster DFT calculations. In addition, the computed bandgaps of the MOF and all complex structures are ∼2.7 eV (450 nm), which is closer to the tested emission wavelength of the MOF. To provide further insights into the change in the luminescence behavior during the reaction, transition dipole moment (TDM) analysis was performed at 346 nm (∼3.5 eV). TDM calculations were performed for all reactants and products to evaluate the positions of substitution in the luminescence response. It is evident from Figure S48 that the square of TDM is approximately 0.5 D^2^ for UiO-66-(COOH)_2_/NDCA. In contrast, irrespective of the reactant substrate, the TDM of adsorbed benzyl alcohol is less than 0.2 D^2^ and could be attributed to quenching of the luminescence. Upon oxidation to benzaldehyde, the TDM of the corresponding products is greater than 0.3 D^2^, which demonstrates reduced quenching and increased luminescence activity.

## Conclusion

In summary, an MOF-based catalytic self-reporting system was developed, which integrates benzyl alcohol oxidation with real-time luminescence reporting, enabling simultaneous catalysis and *in situ* reaction monitoring within a single material. By coupling catalytic activity with an intrinsic optical response, this system continuously converts reaction progress into a measurable luminescence signal, providing direct feedback without interrupting the reaction or relying on repeated off-line analysis.

Beyond the present catalytic system, this work introduces a general strategy for integrating catalytic function with self-reporting capability through rational materials design. Building on this strategy, alternative catalytic sites could be incorporated into the MOF to enable real-time self-reporting of other catalytic transformations, thereby further broadening the application scope of this platform. Such an approach provides a practical route toward more resource-efficient catalytic processes by enabling continuous reaction monitoring, reducing analytical requirements, and facilitating timely process adjustment. Looking forward, coupling DSR catalytic platforms with artificial intelligence and automated data analysis could further enable intelligent reaction monitoring, adaptive process optimization, and more efficient, cost-effective, and controllable catalytic manufacturing.

## Supplementary Material



## Notes

Experimental details, basic characterizations of the relative MOFs, luminescent sensing, sensing mechanism, and related tables for these MOFs toward analytes (PDF)
