# Supplementary material for: A Dynamic Self-Reporting Metal–Organic Framework System for Catalysis and Real-Time Reaction Monitoring
Source: J Am Chem Soc. 2026 Oct 22;148(43):47179–86. doi: 10.1021/jacs.6c15370 (PMC13741826; doi:10.1021/jacs.6c15370)
Supplement: Supplementary file 1 [file ja6c15370_si_001.pdf]

Supporting Information

A Dynamic Self-Reporting Metal-Organic Framework System for Catalysis and Real-Time Reaction Monitoring

Jiatong Huo,[a,†] *Zongsu Han,*[a,†] *Wenxuan Li,*[b,†] *Yihao Yang,*[a] *Zekihan Ozerdem,*[a,c] *Narasimhan Loganathan,*[a,c] *Xincheng Liu,*[d] *Joshua Rushlow,*[a] *Zhaoyi Liu,*[a] *Angela K. Wilson,*[a,c,*] *Kun-Yu Wang,*[e,*] *and Hong-Cai Zhou* [a,*]

[a]Department of Chemistry, Texas A&M University, College Station, Texas 77843, United States

[b]Department of Chemistry, Princeton University, Princeton, New Jersey 08540, United States

[c]Department of Chemistry, Michigan State University, East Lansing, Michigan 44823, United States

[d]Department of Chemistry, The University of Sheffield, Western Bank, Sheffield S10 2TN, United Kingdom

[e]Department of Chemistry, University of Miami, Coral Gables, Florida 33146, United States

Table of Contents

Materials and Methods..5

Synthesis 5

Luminescence Experiments 5

Reaction Monitoring 5

Calculation Methods 5

Figures...7

Tables 34

References 40

Materials and Methods

All reagents were commercially available and used without further purification. Liquid ^1H NMR spectra were recorded on a Bruker Avance NEO 400 NMR spectrometer. PXRD measurements were performed using a Bruker Powder-ECO X-ray diffractometer with Cu-Kα radiation. IR spectra were tested by a Shimadzu IRAffinity-1S spectrometer. TGA curves were obtained under nitrogen atmosphere on a Mettler Toledo TGA/DSC 1 thermogravimetric analyzer from 25 °C to 800 °C. ICP-MS measurements were performed using a PerkinElmer NexION 300D ICP-MS instrument. Luminescence spectra were recorded on a Horiba Fluoromax-4 fluorescence spectrophotometer and a Shimadzu RF-5301 fluorescence spectrophotometer. UV-vis absorption spectra were measured by a Shimadzu UV-2450 absorption spectrometer. Single crystal X-ray diffraction patterns were collected by a Bruker-Axs Venture Ius Cmos Kappa X-ray Apex2 diffractometer with Cu-Kα radiation. 3 mg MOFs for liquid NMR tests were dissolved by 0.5 mL d_6-dimethyl sulfoxide (DMSO) and 5 μL D_2SO_4.

Synthesis

UiO-66-(COOH)$_2$ was synthesized via a solvothermal method[1]. $ZrCl_4$ (0.242 g, 1.00 mmol) and H_4PMA (0.254 g, 1.00 mmol) were dissolved in 6 mL H_2O and 4 mL acetic acid. The mixture was sonicated for 15 minutes and then heated at 120 °C for 24 hours in a 20 mL vial. After cooling to room temperature, the resulting sample was collected by centrifugation, washed three times with deionized water and methanol, respectively.

UiO-66-(COOH)$_2$/NDCA was synthesized via a linker exchange method. To prepare the mixed-ligand MOF via a linker exchange method, 20 mg UiO-66-(COOH)$_2$ was soaked in a 0.05 M H_2NDCA solution in 3 mL DMF at 100 °C for 24 hours. Then washed three times with fresh DMF.

Ag@UiO-66-(COOH)$_2$/NDCA was synthesized via a post-synthetic coordination method. 20 mg UiO-66-(COOH)$_2$/NDCA was immersed in a 0.1 M $AgNO_3$ solution in 3 mL DMF at 80 °C for 24 hours. The solid sample was separated by centrifugation, thoroughly washed three times with DMF and methanol, and dried at 80 °C.

Luminescence Experiments

MOFs were ground into fine powder and dispersed in DMF by ultrasonication for 10 minutes to form a stable suspension with a concentration of 0.15 mg mL^{-1}.

Reaction Monitoring

Benzyl alcohol (0.1081 g, 1.00 mmol) or an analogous substrate (1.00 mmol), $Na_2S_2O_8$ (0.5953 g, 2.50 mmol), and the Ag@UiO-66-(COOH)$_2$/NDCA (9 mg) were added to DMF (6 mL), giving a catalyst concentration of 1.5 mg mL^{-1}. The resulting reaction mixture was then heated to 60 °C. At the first stage, emission spectra were taken every 5 minutes. Then the measurements were taken every 10 minutes for the relatively stable signals. At the same time, 5 μL supernatant was added to 0.5 mL d_6-DMSO for subsequent NMR analysis.

Calculation Methods

All cluster DFT calculations were performed using ORCA software package (version 5.0.4)[2,3]. The geometry optimization for all molecules were carried out at r^2SCAN-3c/def2-mTZVVP/def2-mTZVVP/J[4] level of theory. Calculations were corrected utilizing the geometrical counterpoise correction gCP[5] and the atom-pairwise dispersion correction based on tight binding partial charges (D4)[6,7]. The optimized structures were then used for symmetry-adapted perturbation theory (SAPT)[8] analysis to decompose the interaction energies. These SAPT calculations were carried out at the SAPT2+/aug-cc-pVQZ level of theory with the Psi4 package[9]. To visualize the nature of the intermolecular interactions, an analysis based on the independent gradient model based on Hirshfeld partitioning (IGMH)[10] was conducted using the Multiwfn program[11,12], and all figures were rendered with Visual Molecular Dynamics (VMD)[13].

The periodic calculations for UiO-66-$(COOH)_2$-NDCA were performed using the Vienna *ab initio* Simulation Package (VASP)[14-16] under the Kohn-Sham DFT framework (version 6.5.1). The projected augmented wave function (PAW)[17] pseudopotentials were used for all of the calculations. The periodic model is composed of a face centered cubic cell with the lattice constant of 14.67 Å. The Perdew-Burke-Ernzerhof (PBE)[18] exchange-correlation functional with Grimme dispersion correction (D3) with Becke-Johnson damping function[19] was used for structure optimization. A kinetic energy cutoff of 500 eV, energy convergence threshold of 10^{-6} eV and force convergence threshold of 0.01 eV/Å were used for all of the calculations. Notably, all atoms and the lattice constants were relaxed using a conjugate gradient algorithm at the

gamma-centered 1×1×1 k-point grid (Γ) point.

To compute accurate band gap structure, the computationally intensive HSE06[20] hybrid approach was used in the single point SCF calculations at the Γ-point on the optimized geometries with the same kinetic energy cutoff and electronic structure convergence threshold as for the PBE geometry optimization calculations. The transition dipole moments were calculated by VASPKIT[21] and the structural visualizations were performed using CCDC Mercury software[22].

Figures

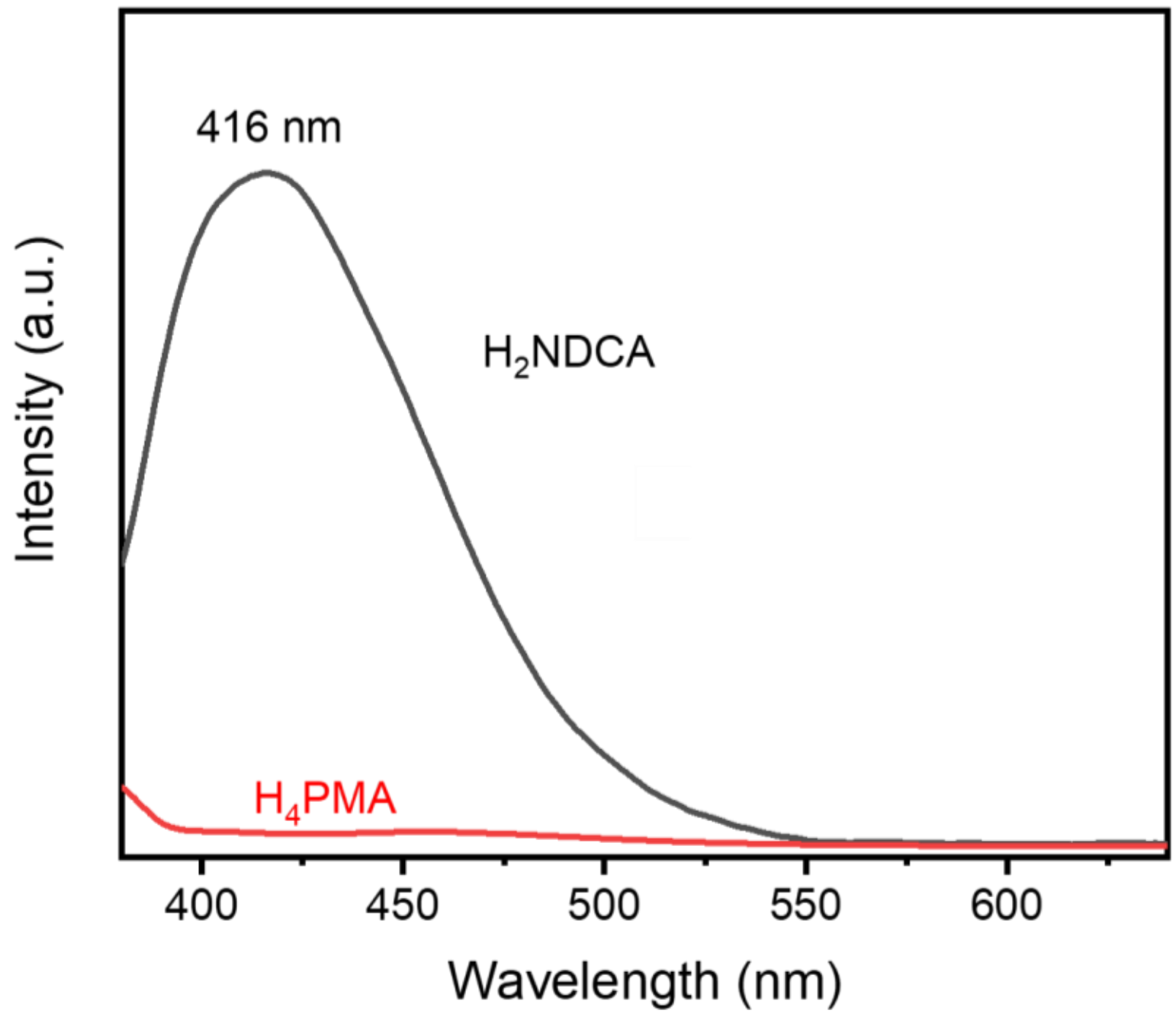

Figure S1. Emission spectra of H_2NDCA and H_4PMA.

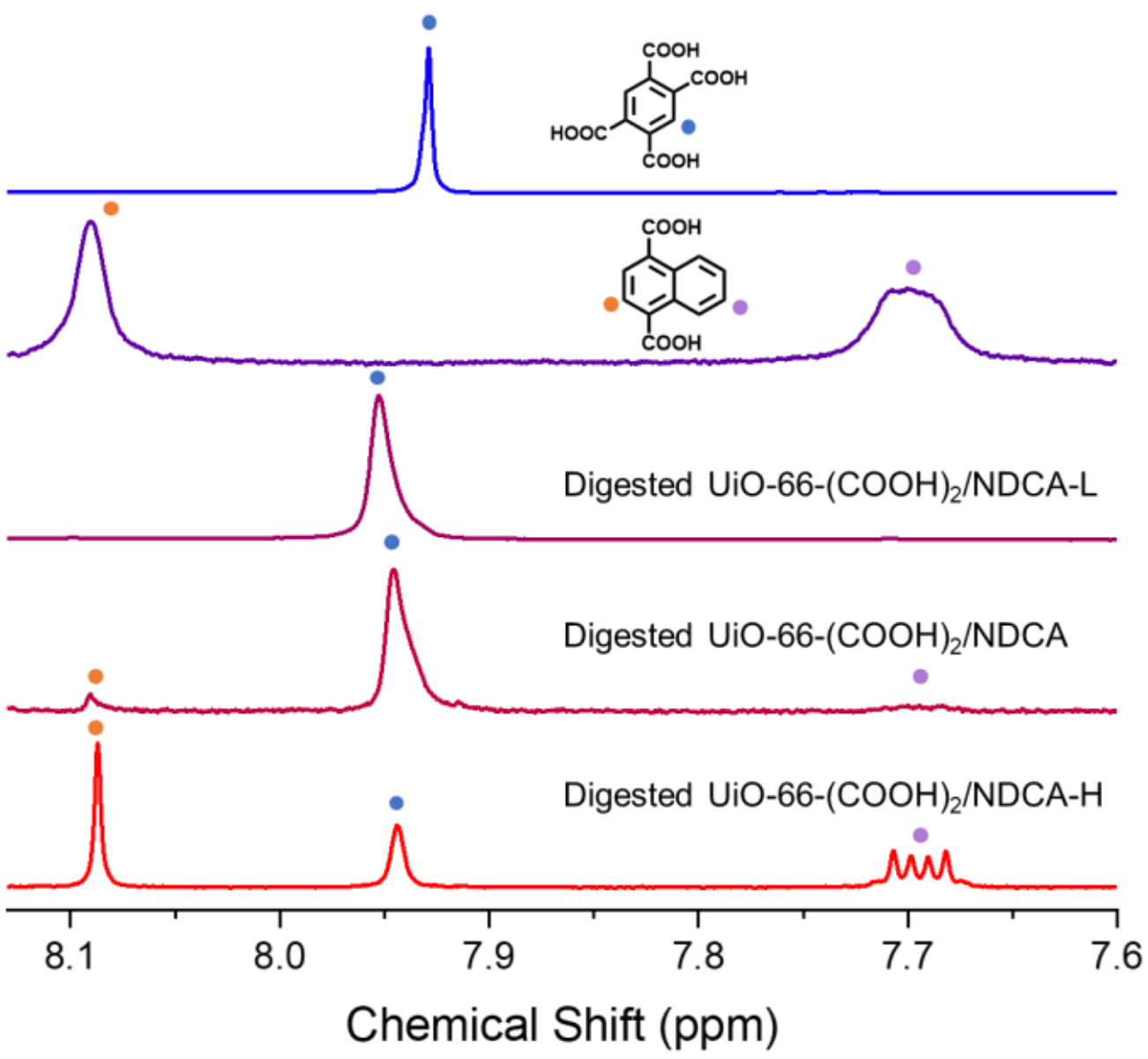

Figure S2. ^{1}H NMR spectra of H_4PMA, H_2NDCA UiO-66-$(COOH)_2$/NDCA-L ([H_2NDCA] = 0.005 M), UiO-66-$(COOH)_2$/NDCA ([H_2NDCA] = 0.05 M) and UiO-66-$(COOH)_2$/NDCA-H ([H_2NDCA] = 0.5 M).

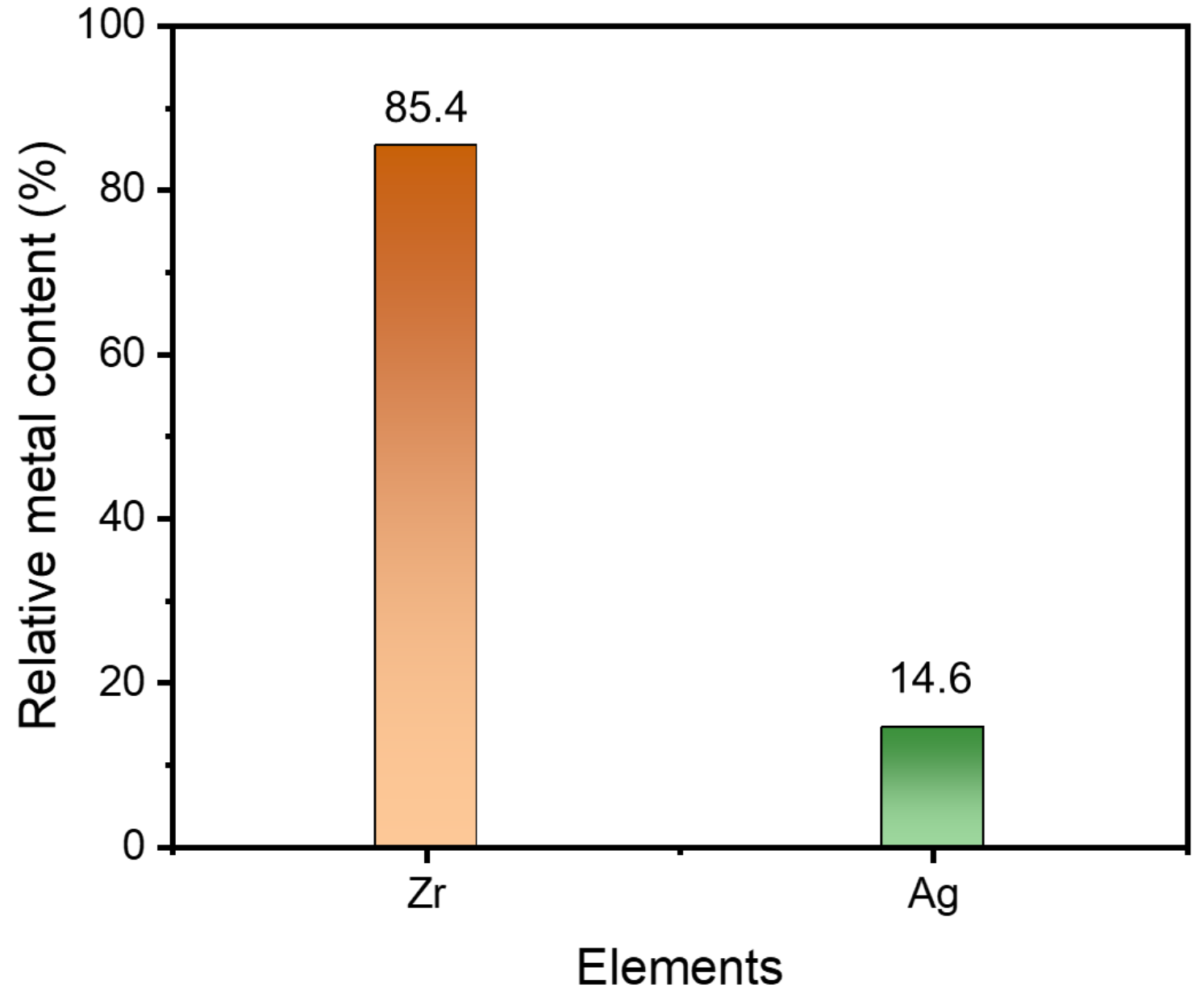

Figure S3. ICP-MS results of Ag@UiO-66-$(COOH)_2$/NDCA.

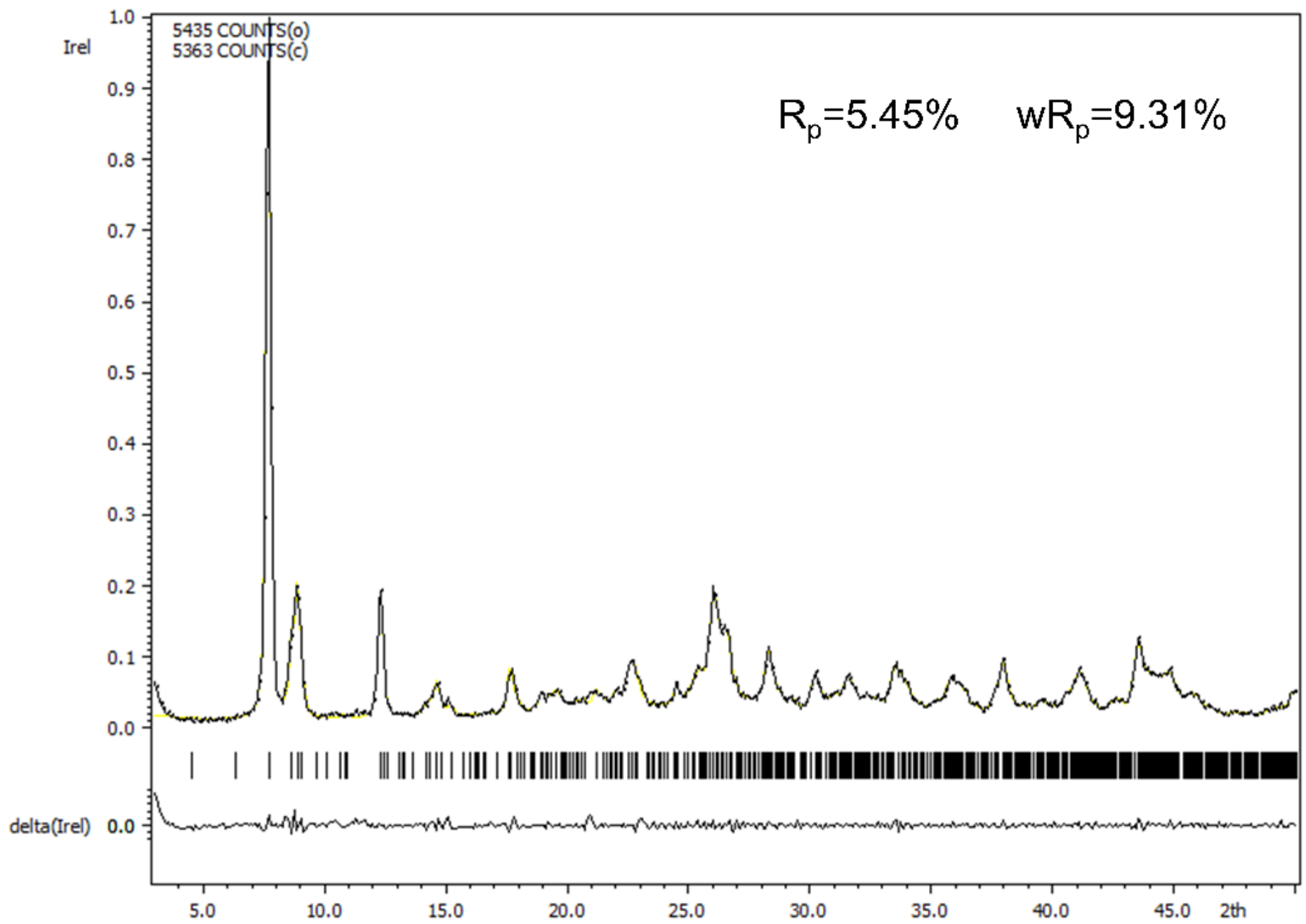

Figure S4. PXRD pattern of UiO-66-$(COOH)_2$.

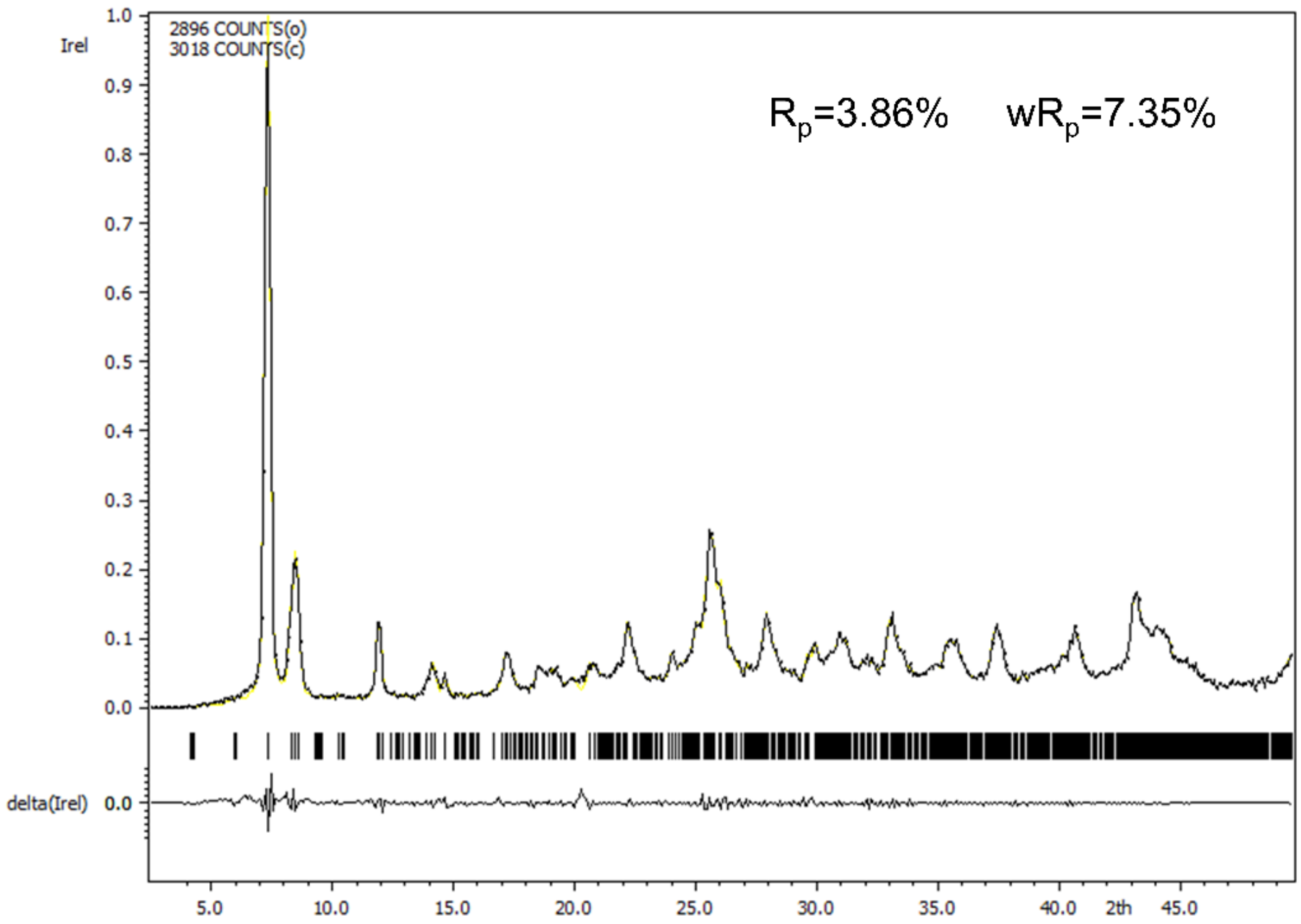

Figure S5. PXRD pattern of UiO-66-$(COOH)_2$/NDCA.

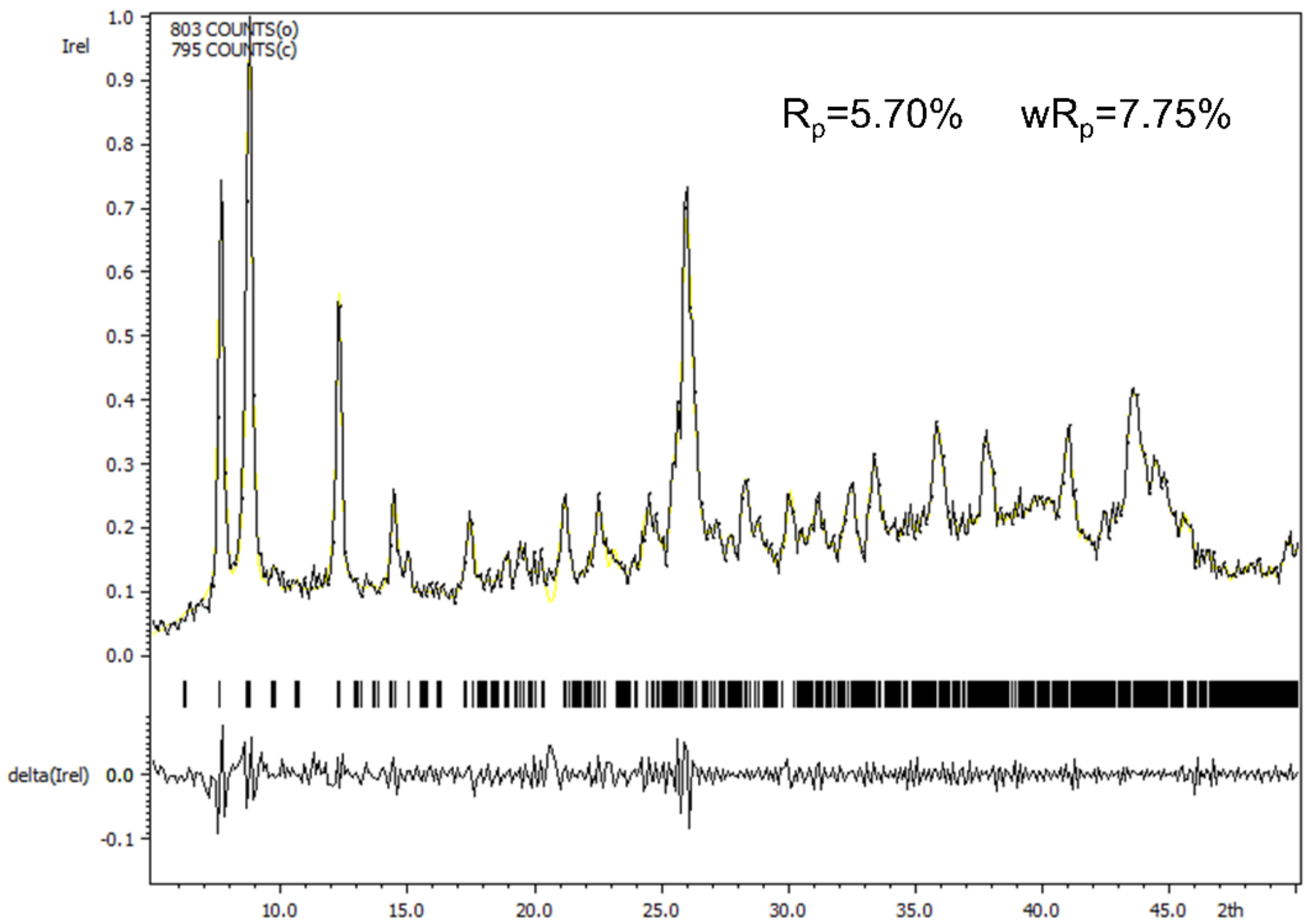

Figure S6. PXRD pattern of Ag@UiO-66-$(COOH)_2$/NDCA.

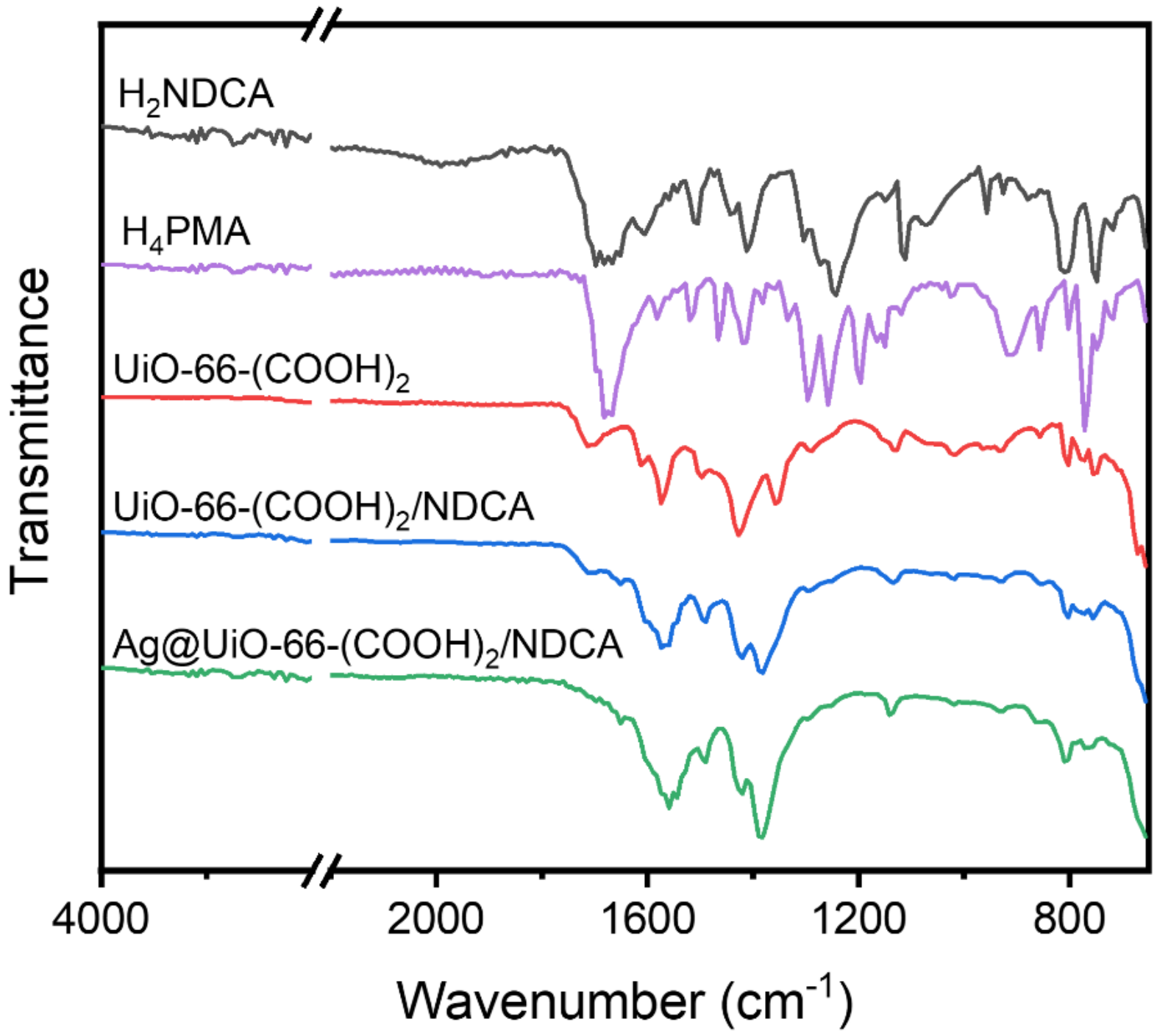

Figure S7. IR spectra of H_2NDCA, H_4PMA, UiO-66-$(COOH)_2$, UiO-66-$(COOH)_2$/NDCA and Ag@UiO-66-$(COOH)_2$/NDCA.

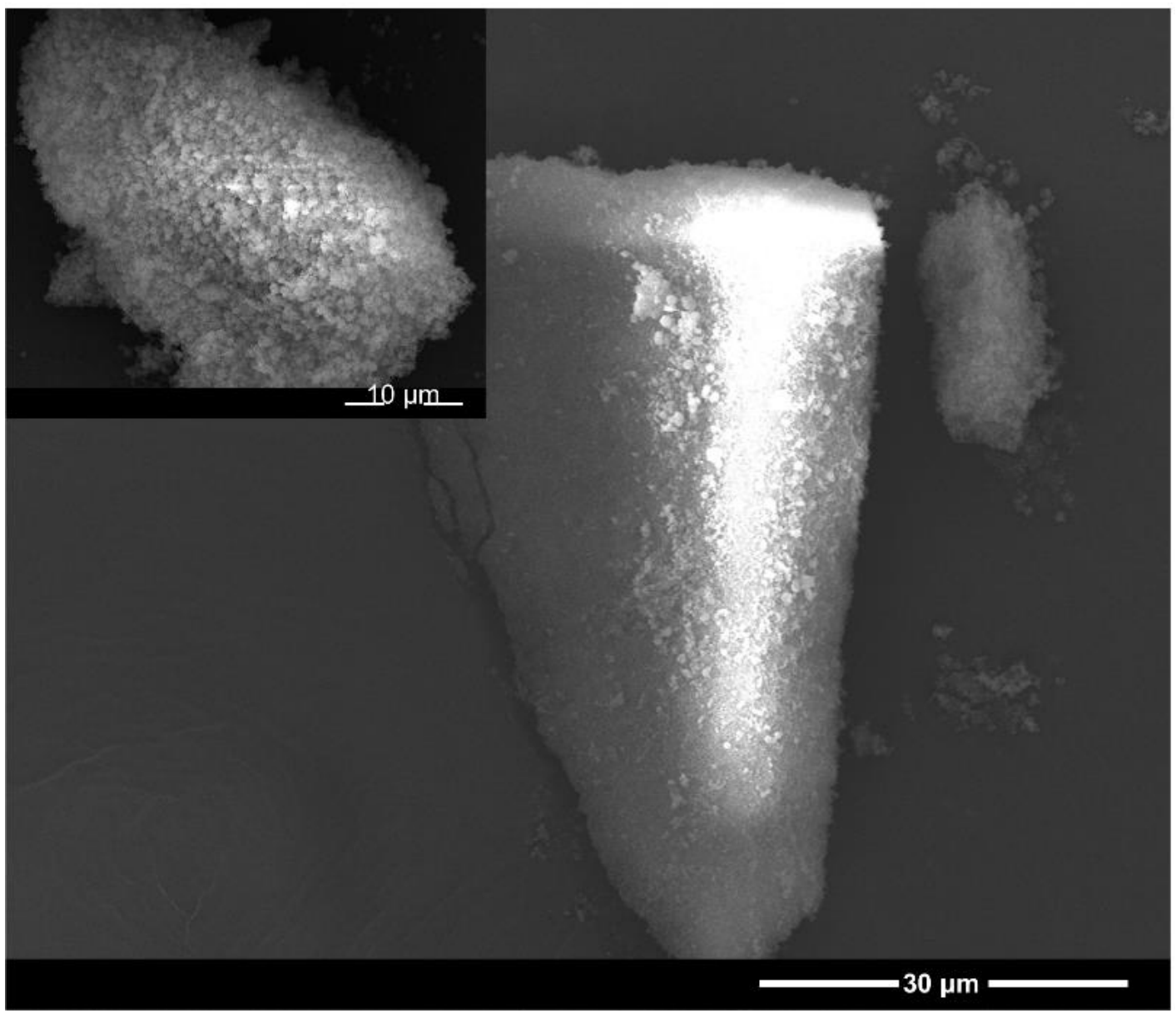

Figure S8. SEM images of UiO-66-$(COOH)_2$.

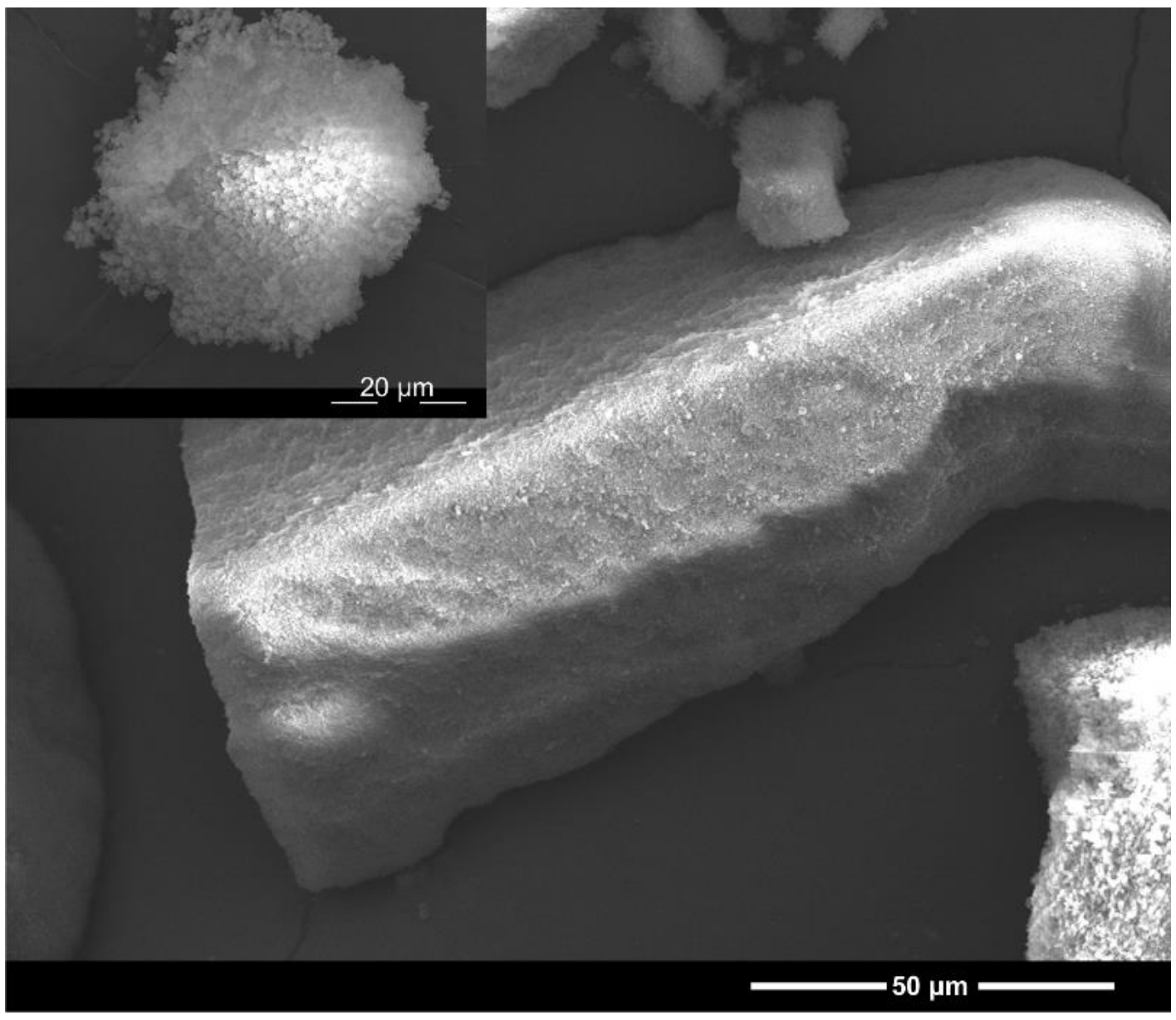

Figure S9. SEM images of UiO-66-$(COOH)_2$/NDCA.

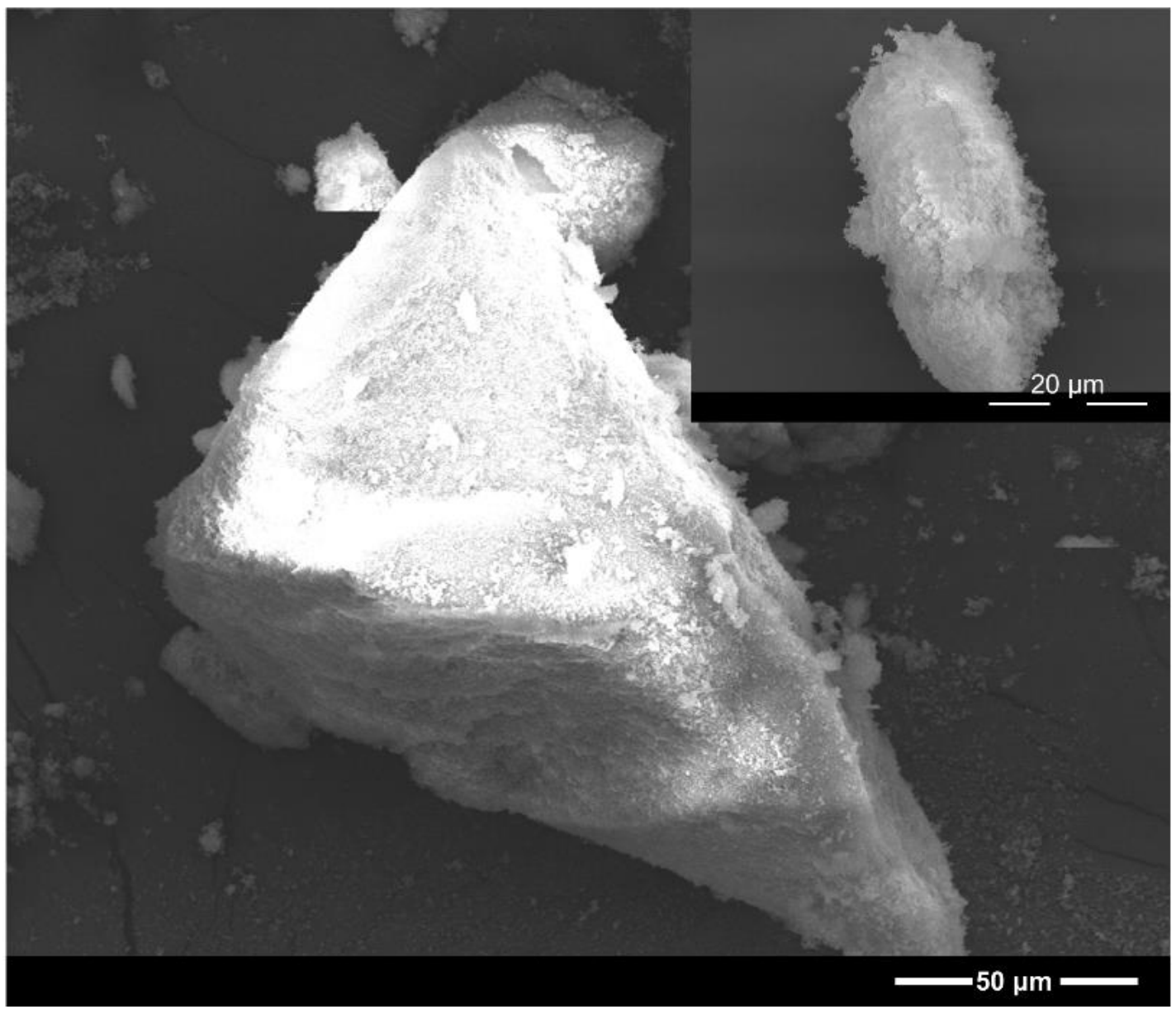

Figure S10. SEM images of Ag@UiO-66-$(COOH)_2$/NDCA.

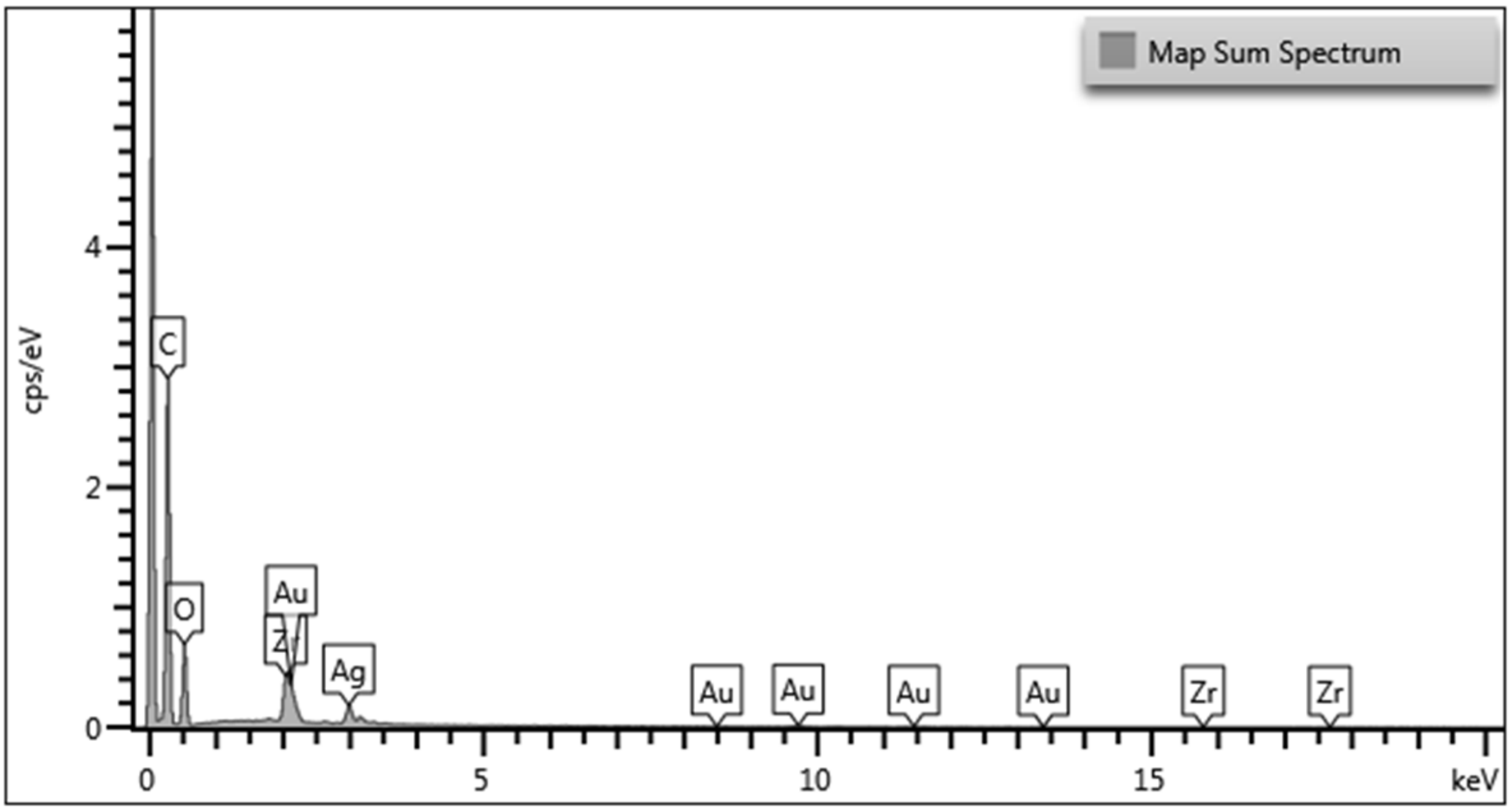

Figure S11. EDS pattern of Ag@UiO-66-$(COOH)_2$/NDCA.

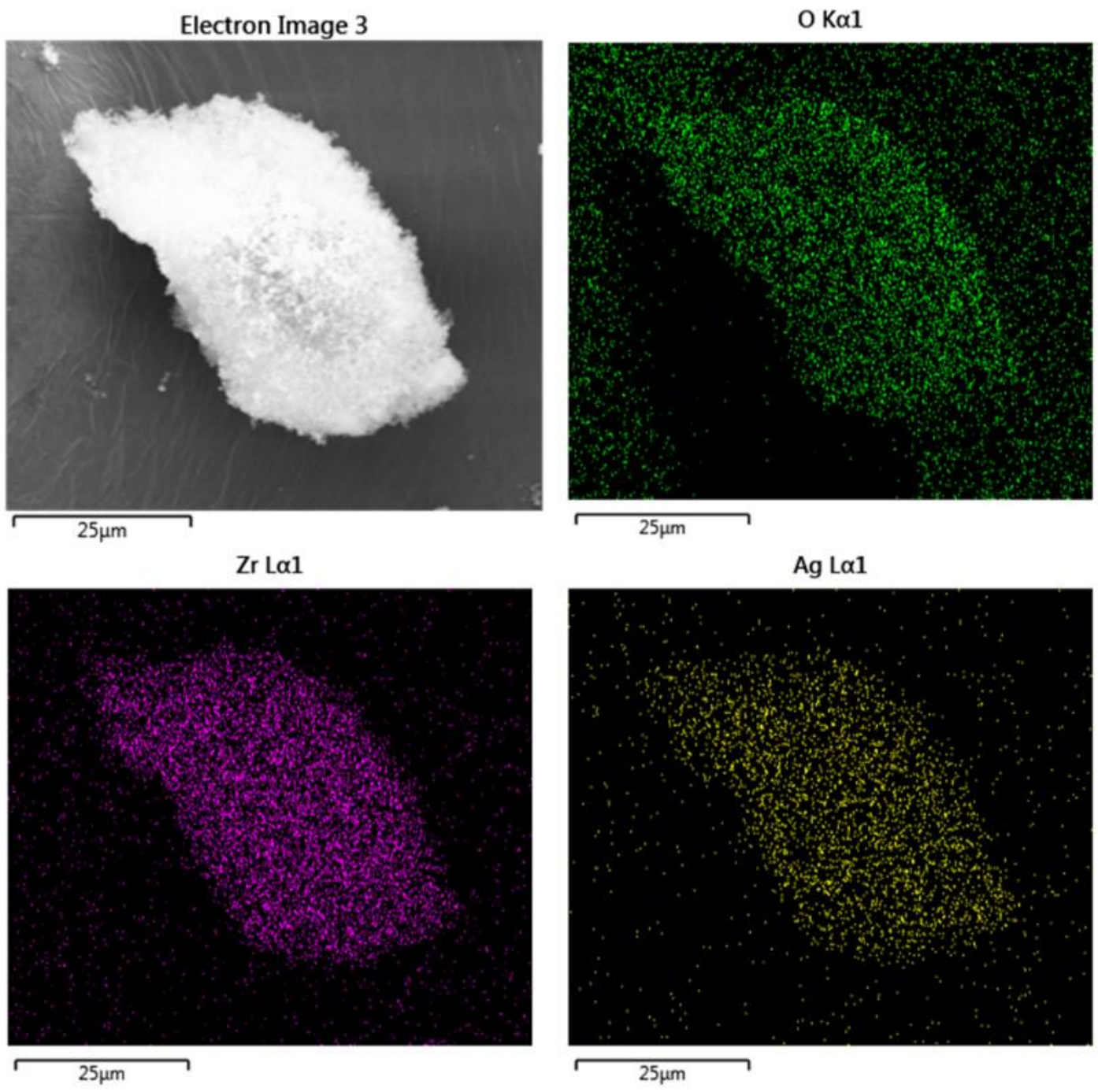

Figure S12. EDS mapping patterns of Ag@UiO-66-$(COOH)_2$/NDCA (Elemental mapping of O, Zr and Ag).

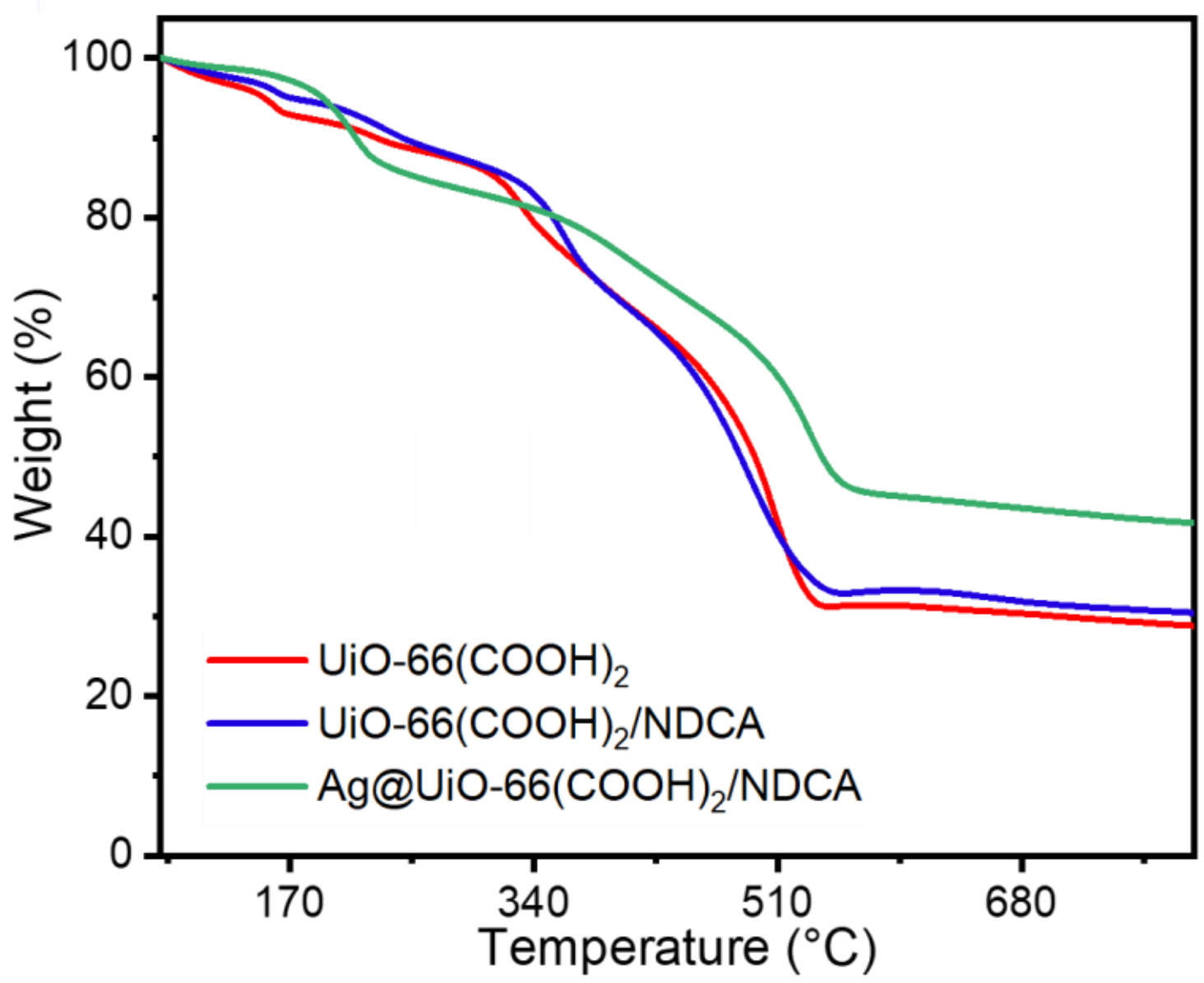

Figure S13. TGA curves of UiO-66-$(COOH)_2$, UiO-66-$(COOH)_2$/NDCA and Ag@UiO-66-$(COOH)_2$/NDCA.

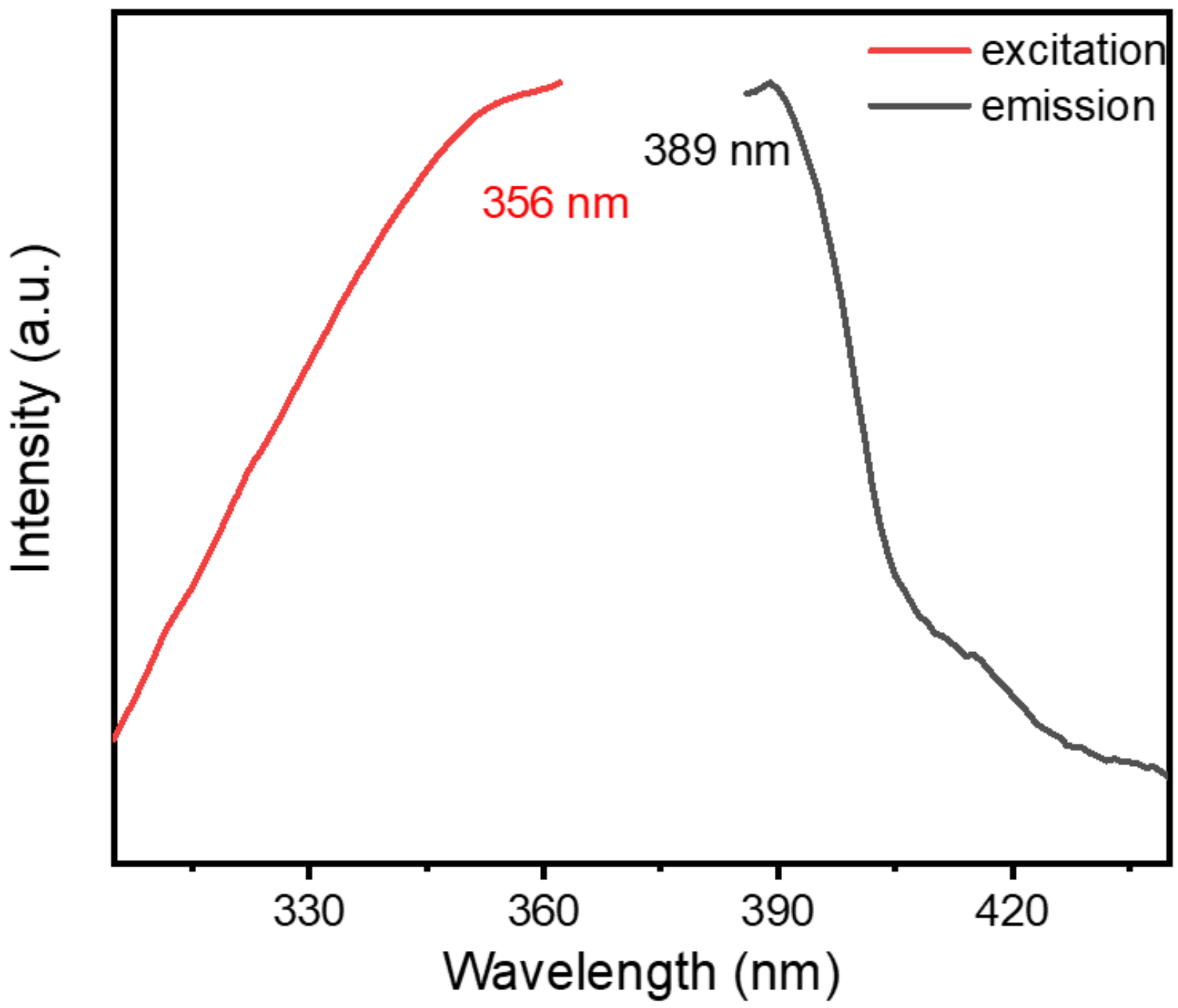

Figure S14. Excitation and emission spectra of UiO-66-$(COOH)_2$.

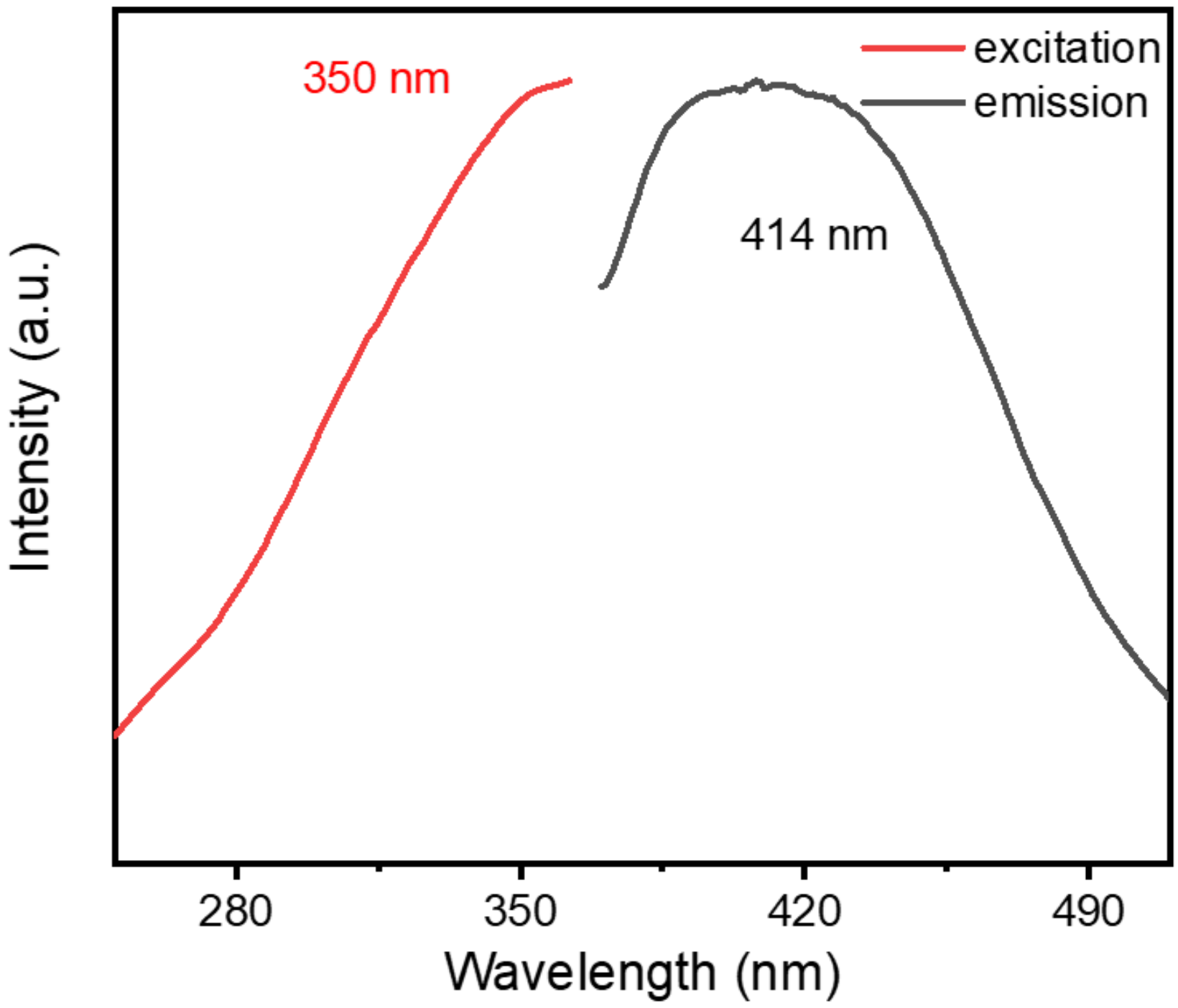

Figure S15. Excitation and emission spectra of UiO-66-$(COOH)_2$/NDCA.

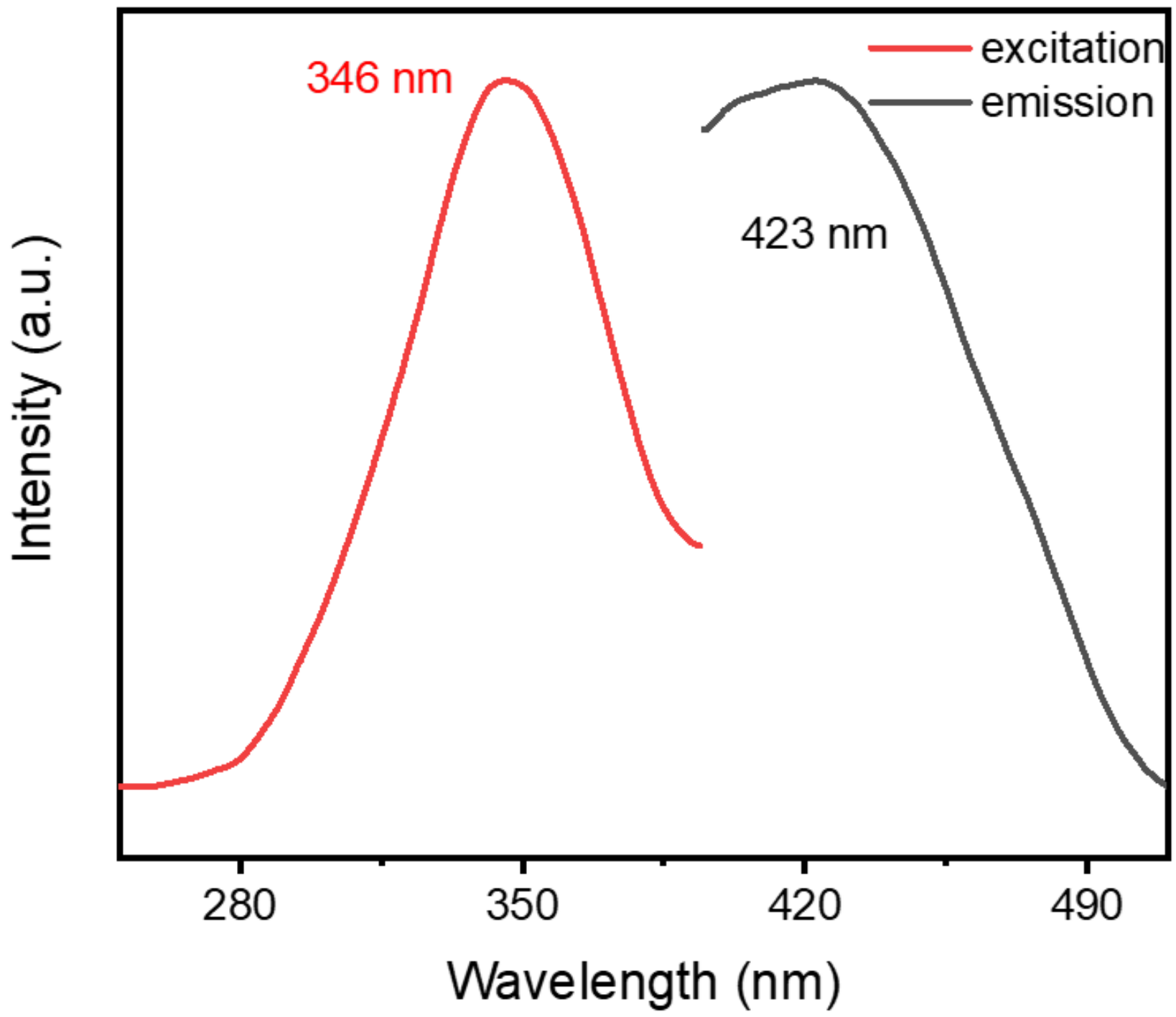

Figure S16. Excitation and emission spectra of Ag@UiO-66-$(COOH)_2$/NDCA.

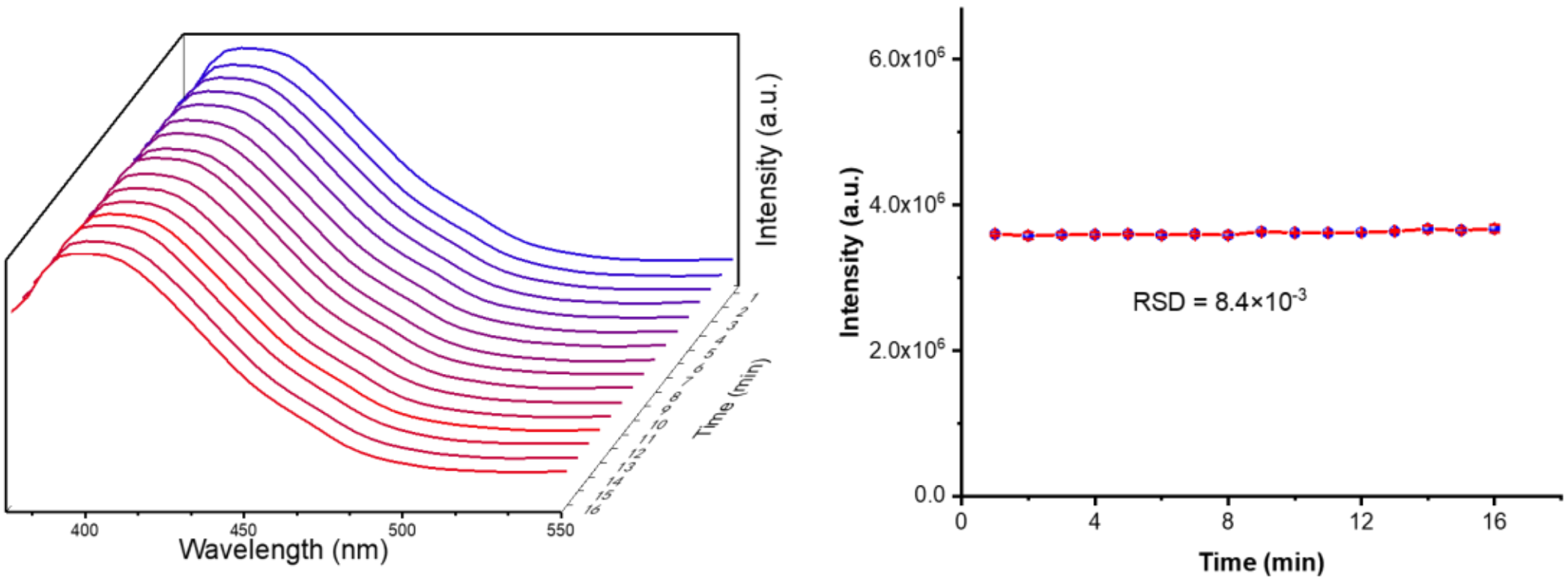

Figure S17. Emission spectra and intensity changes of Ag@UiO-66-$(COOH)_2$/NDCA with time at room temperature.

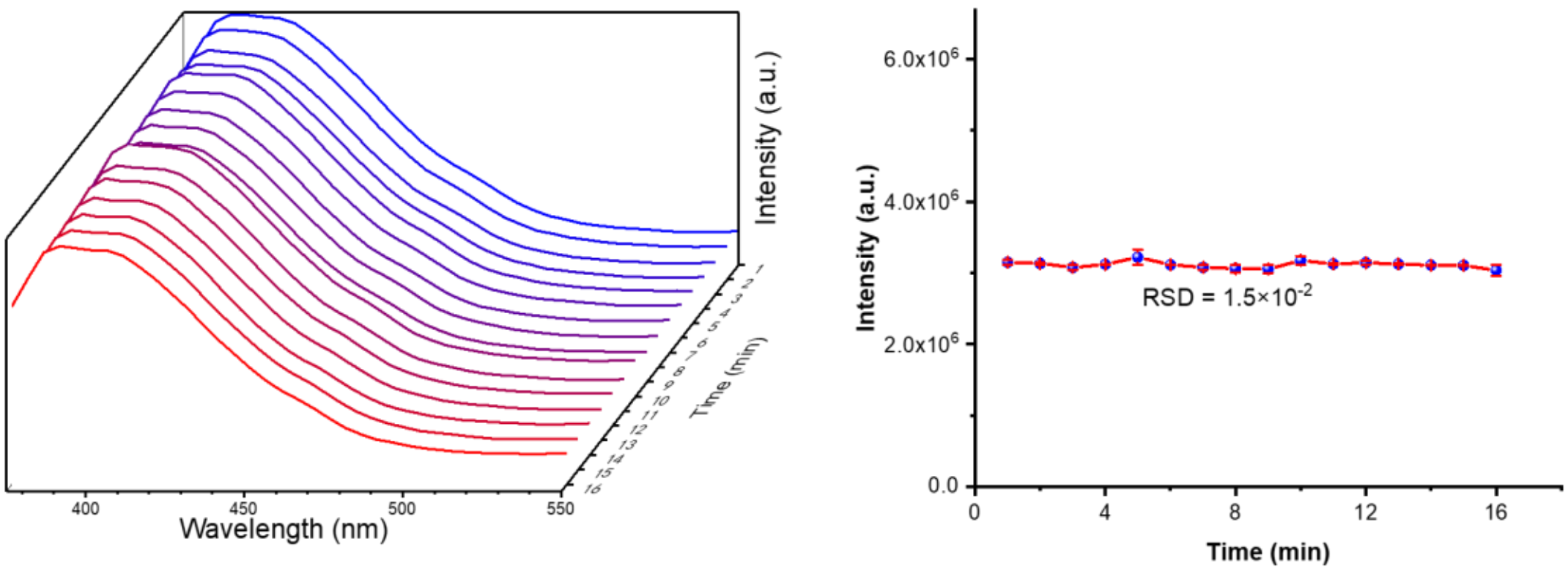

Figure S18. Emission spectra and intensity changes of Ag@UiO-66-$(COOH)_2$/NDCA with time at 60 °C.

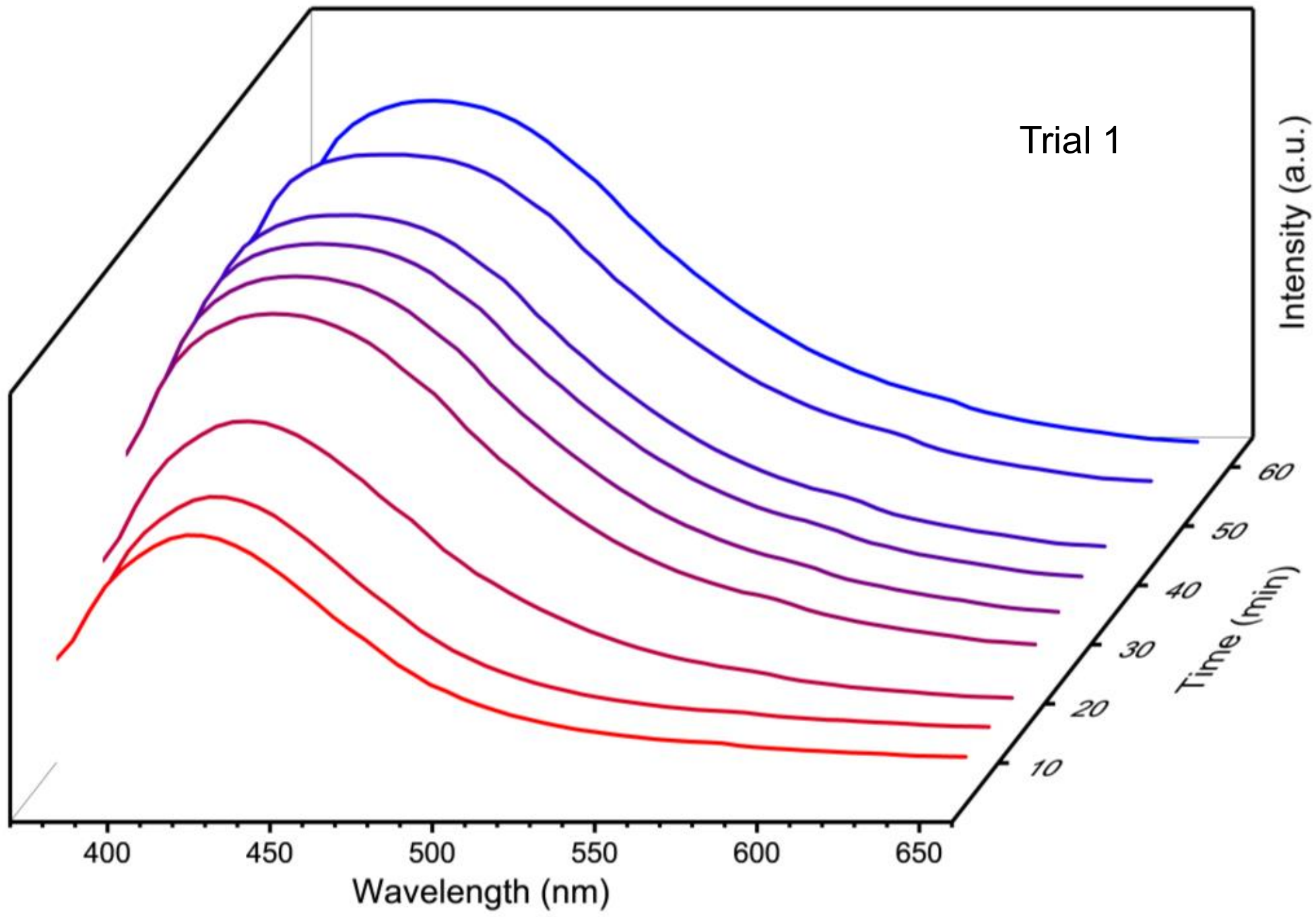
Trial 1
Intensity (a.u.)
400 450 500 550 600 650
Wavelength (nm)
10 20 30 40 50 60
Time (min)

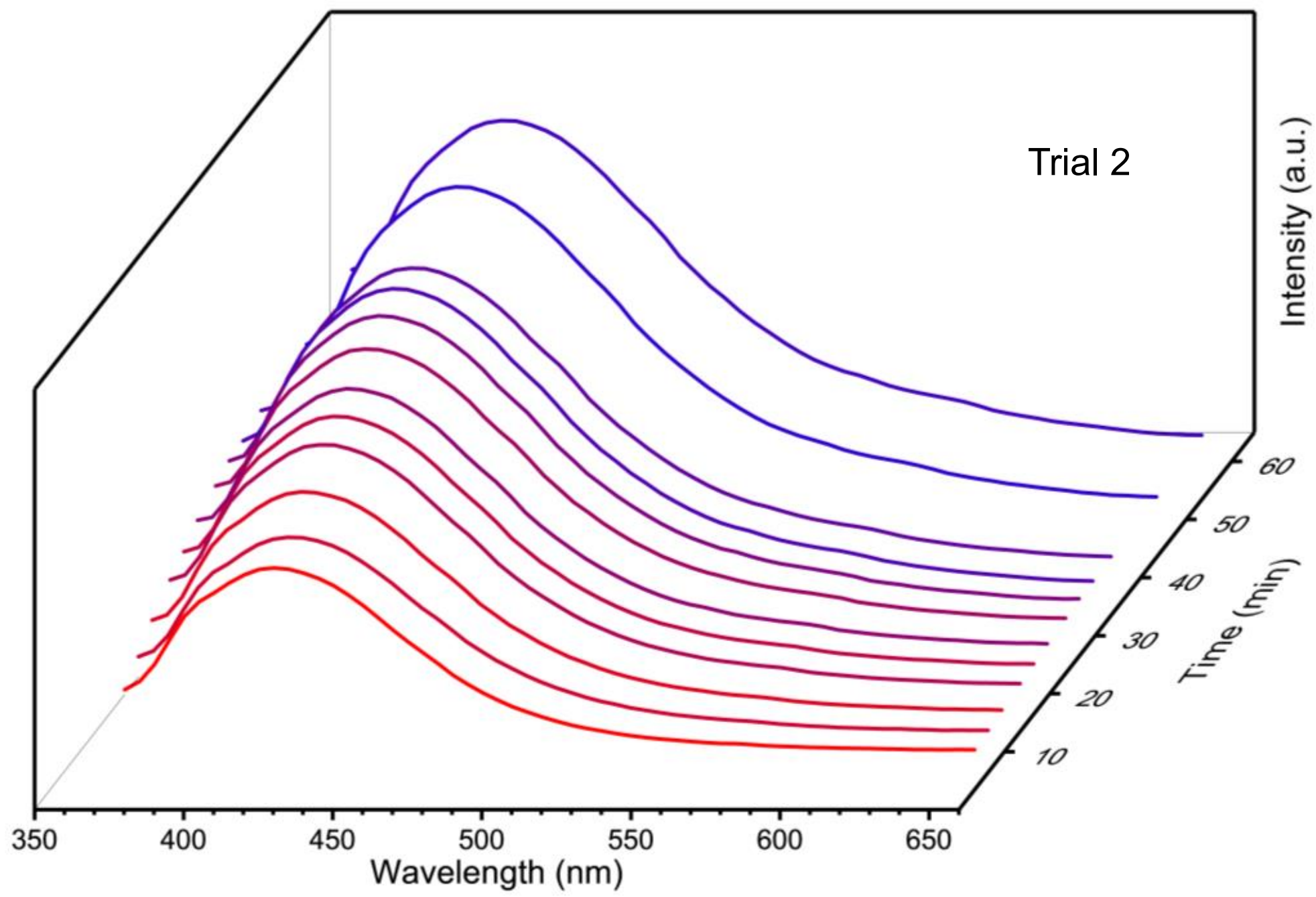
Trial 2
Intensity (a.u.)
350 400 450 500 550 600 650
Wavelength (nm)
10 20 30 40 50 60
Time (min)

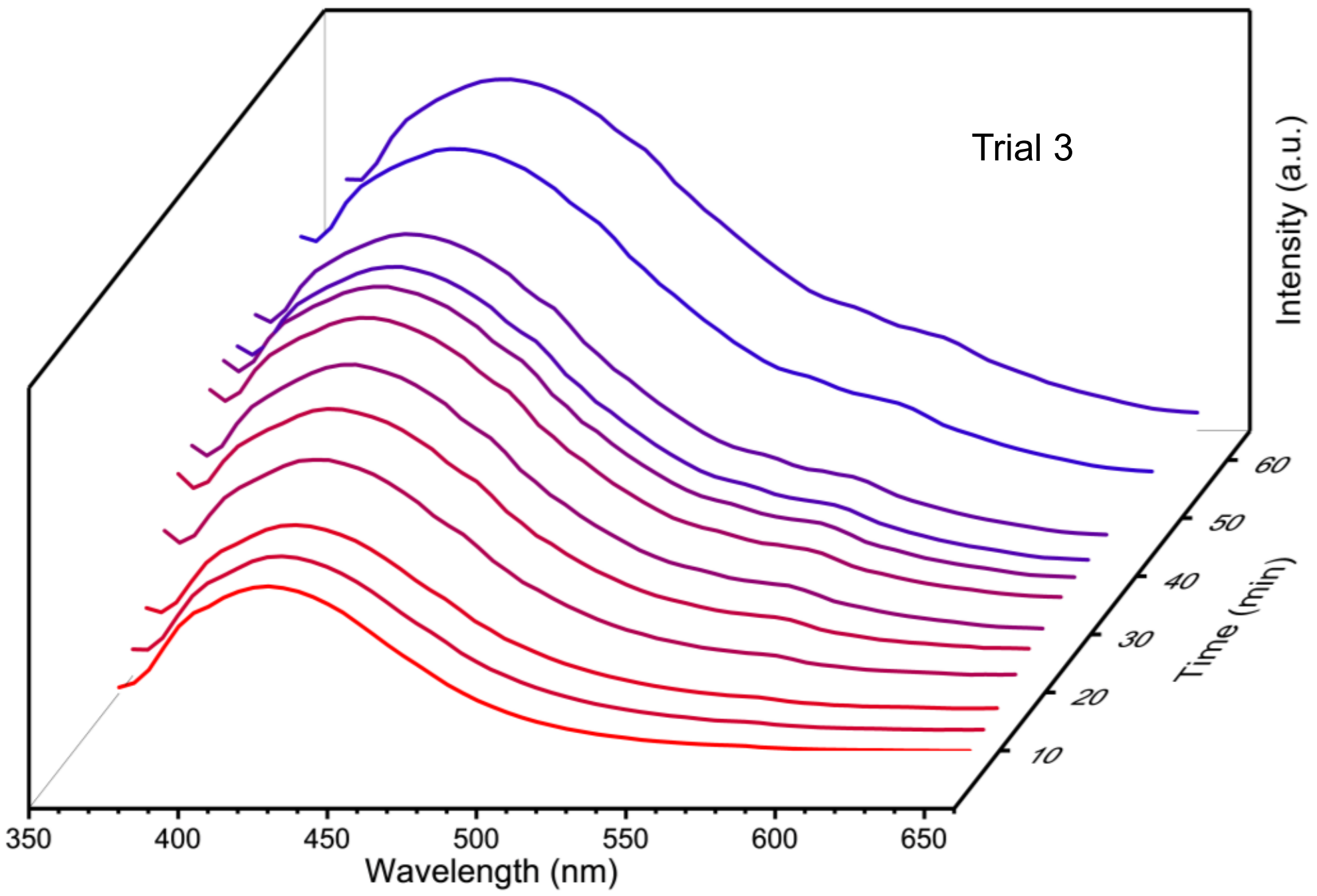

Figure S19. Reproducibility of the reaction. Three additional runs show consistent changes in the emission spectra during the reaction.

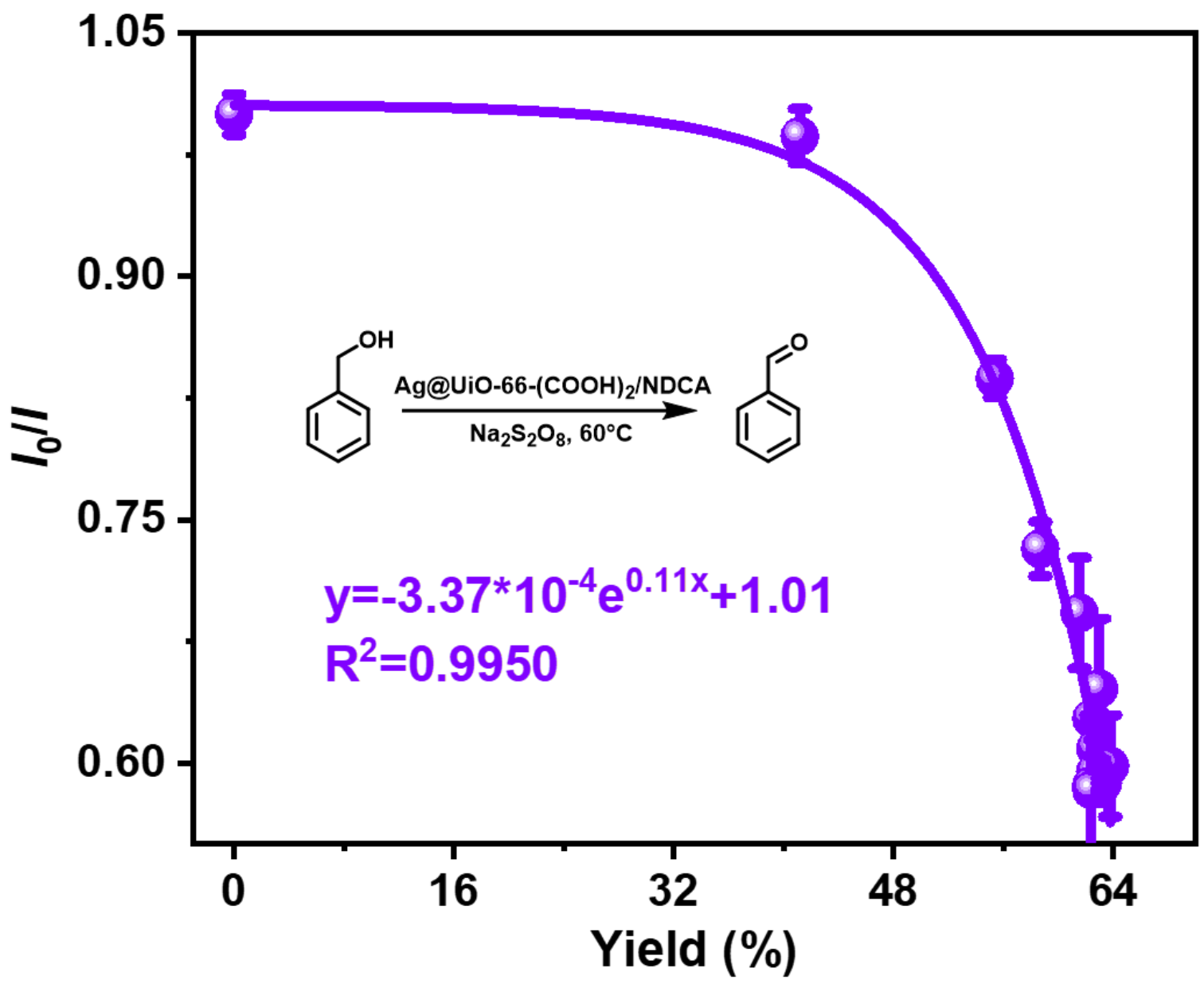

Figure S20. Fitting equation between emission intensity changes and product yields for reactant **1**.

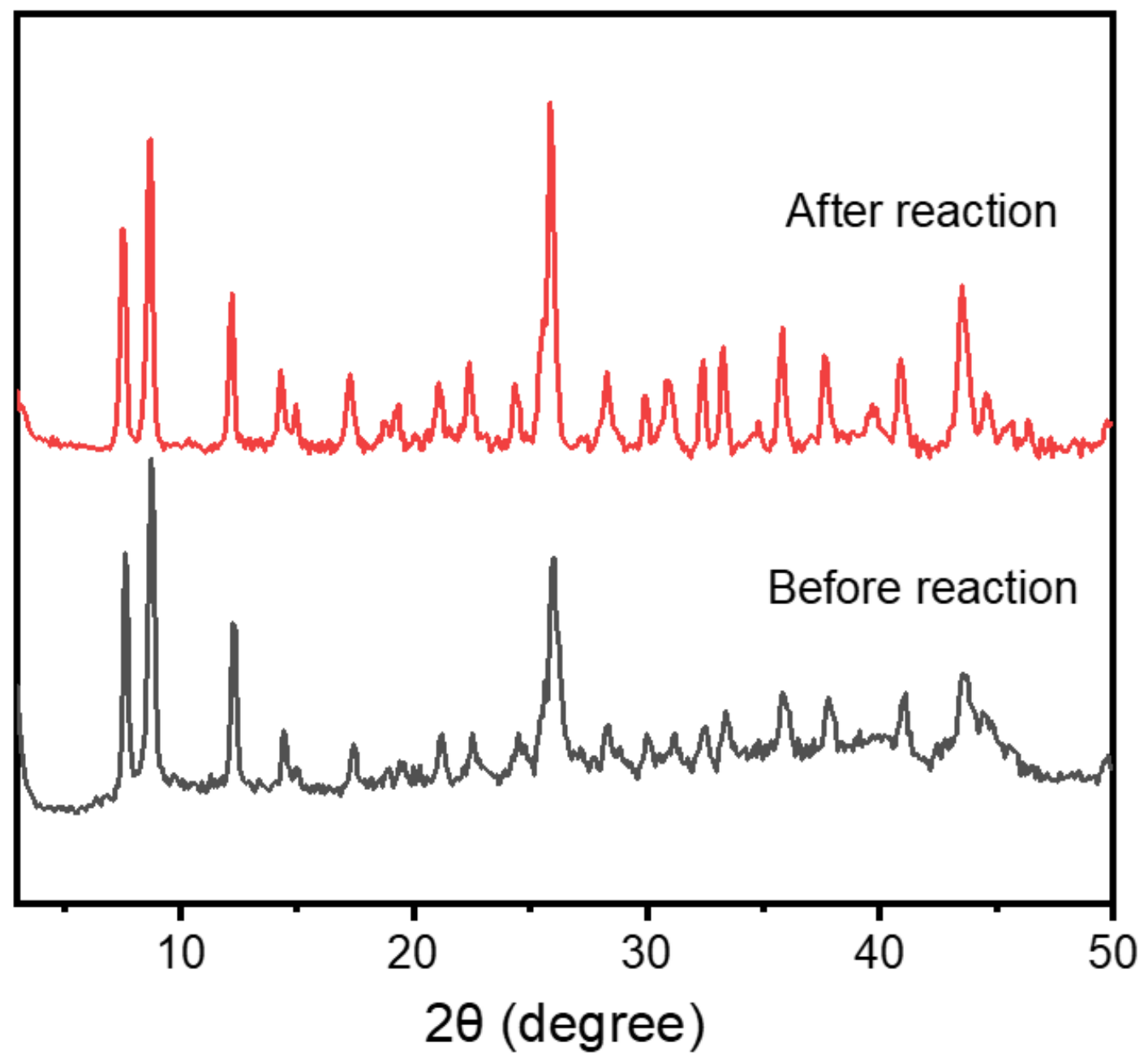

Figure S21. PXRD patterns of Ag@UiO-66-$(COOH)_2$/NDCA before and after the reaction.

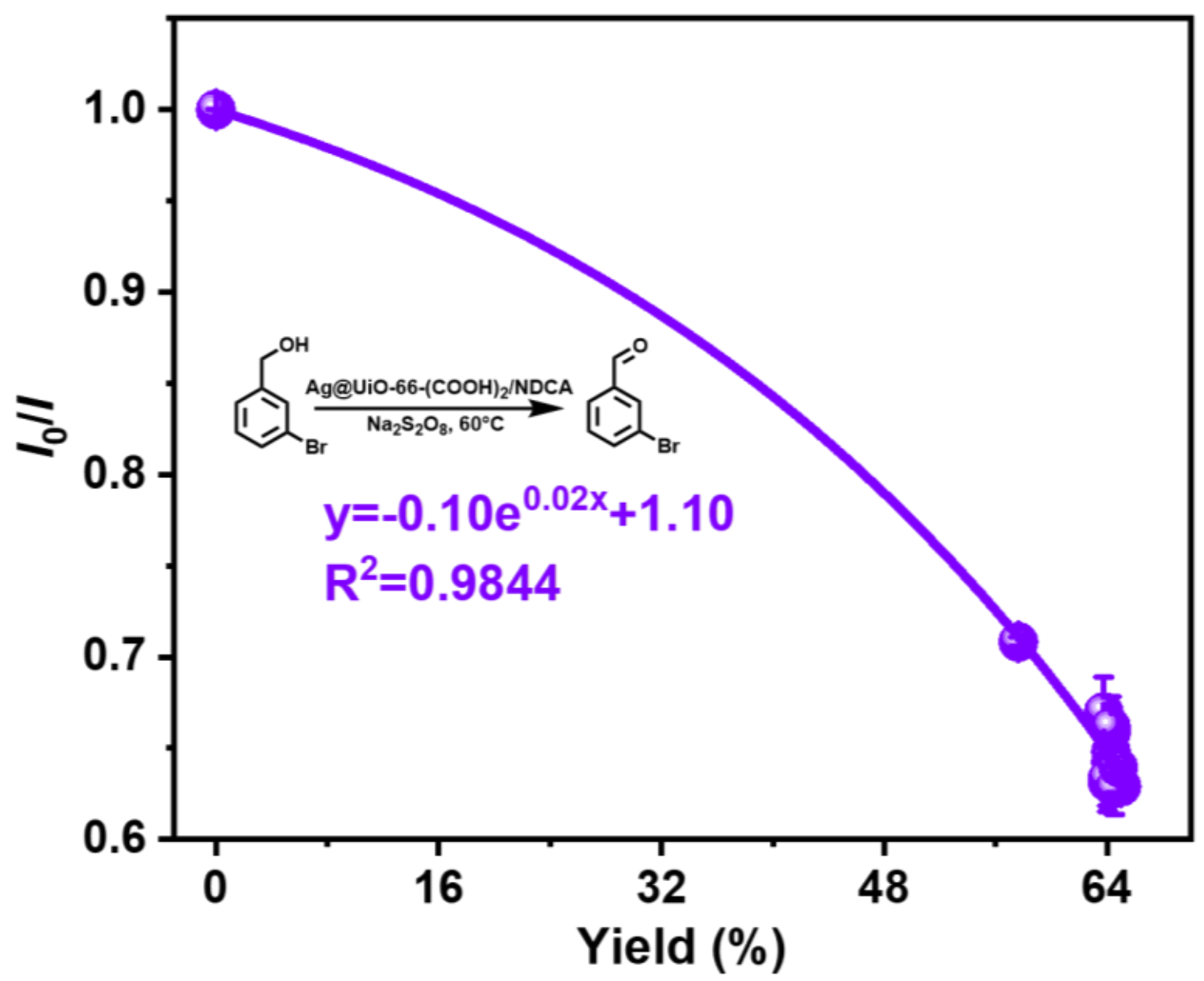

Figure S22. Fitting equation between emission intensity changes and product yields for reactant **2**.

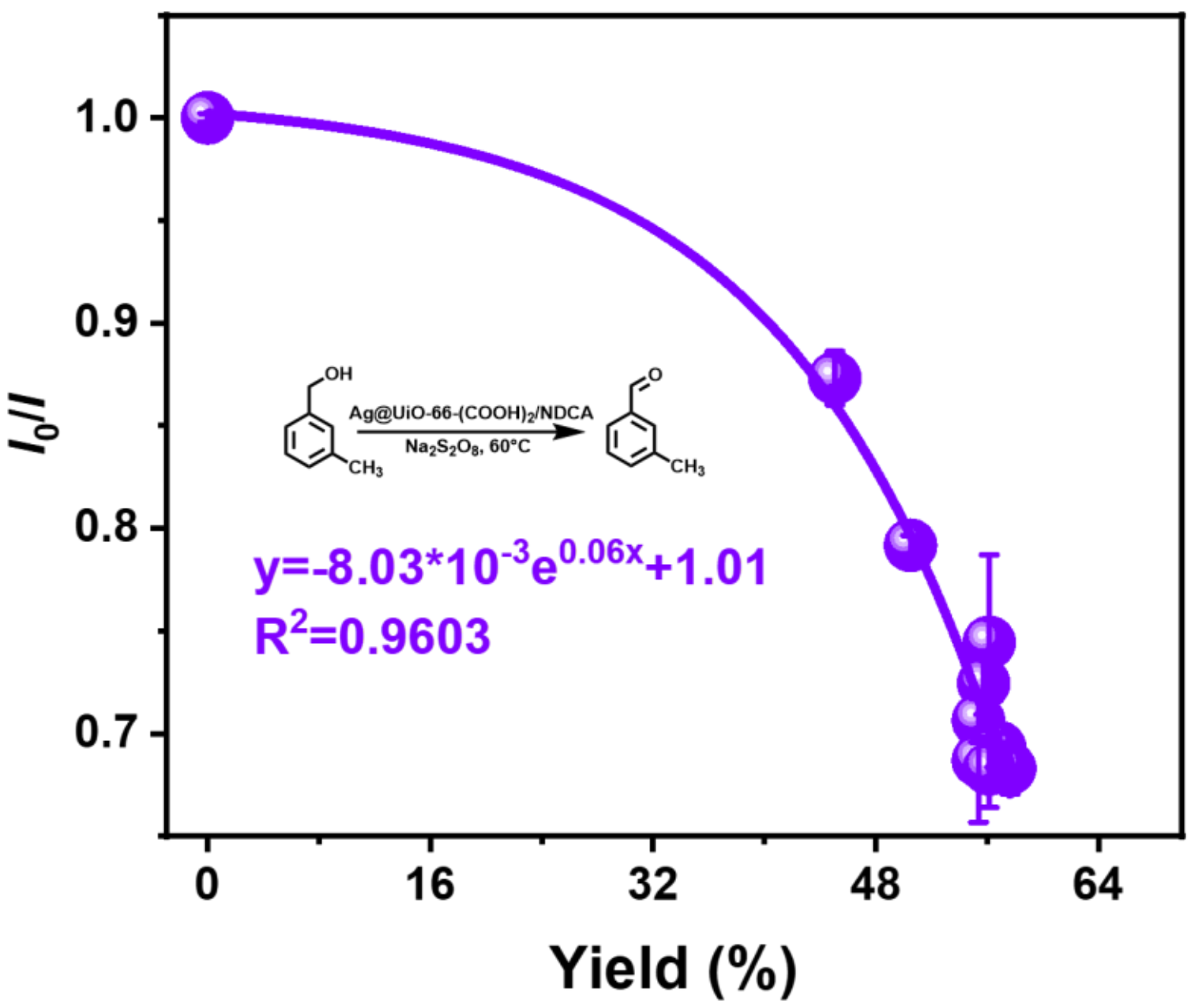

Figure S23. Fitting equation between emission intensity changes and product yields for reactant **3**.

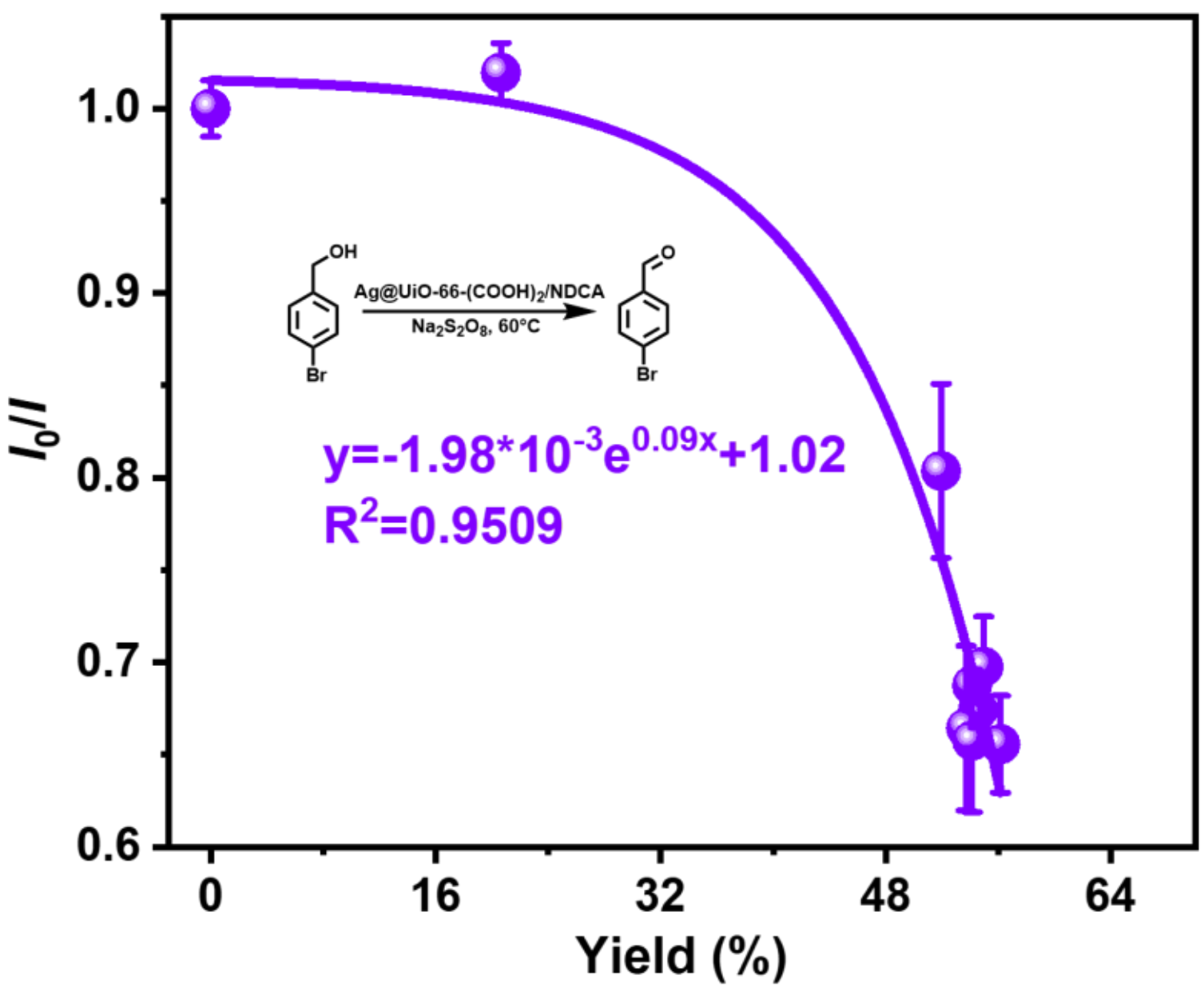

Figure S24. Fitting equation between emission intensity changes and product yields for reactant **4**.

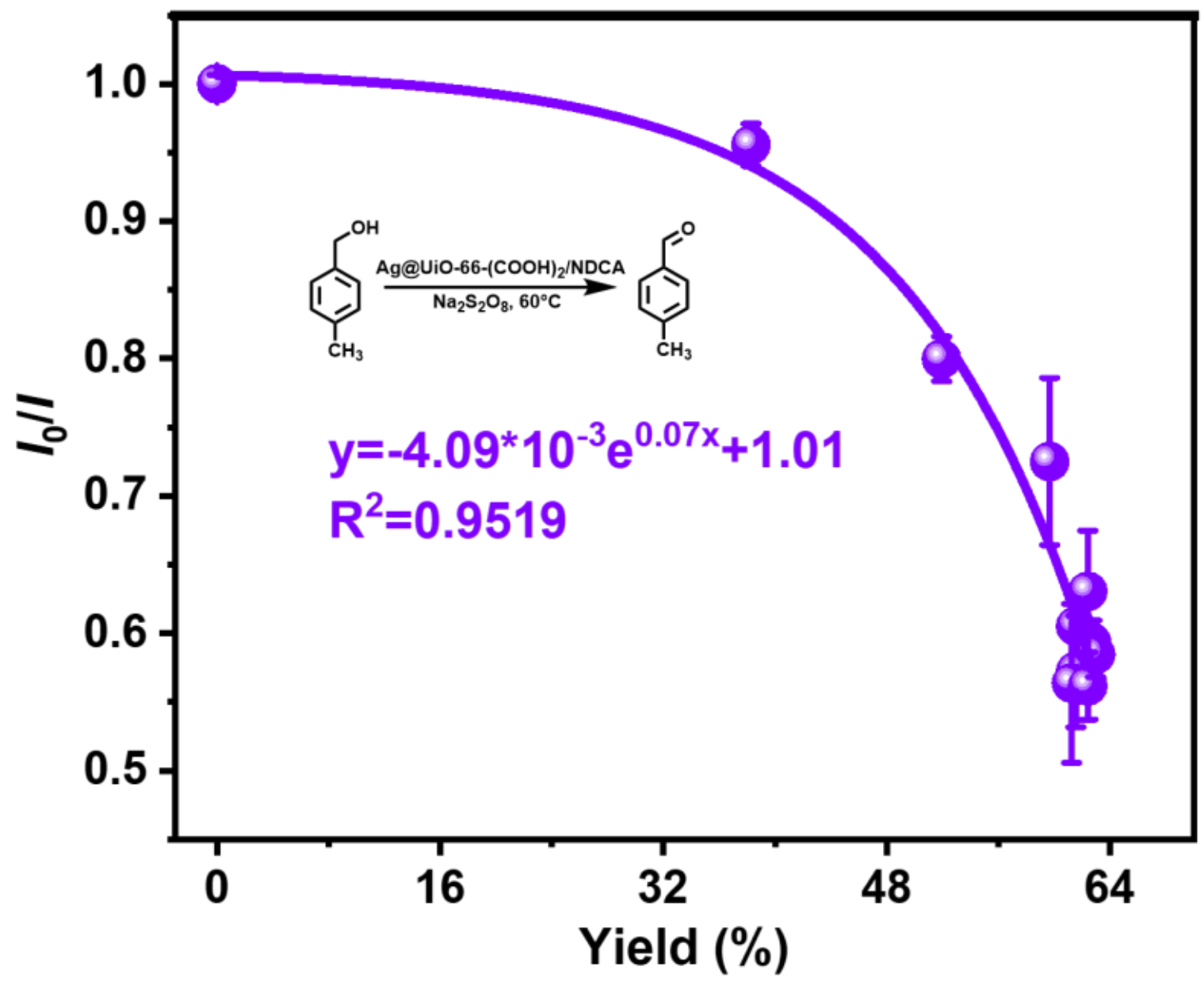

Figure S25. Fitting equation between emission intensity changes and product yields for reactant **5**.

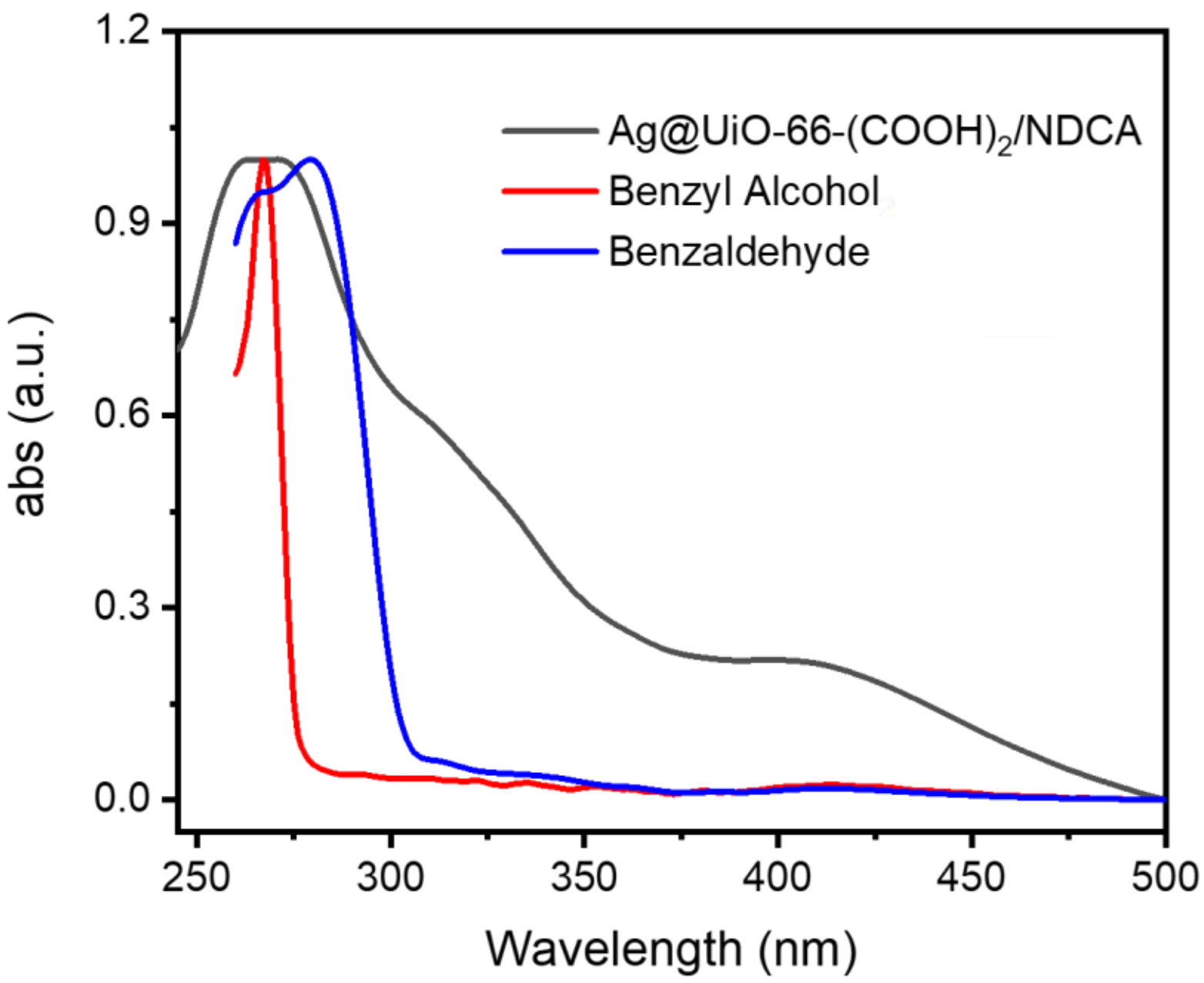

Figure S26. UV-vis spectra of Ag@UiO-66-$(COOH)_2$/NDCA, benzyl alcohol and benzaldehyde.

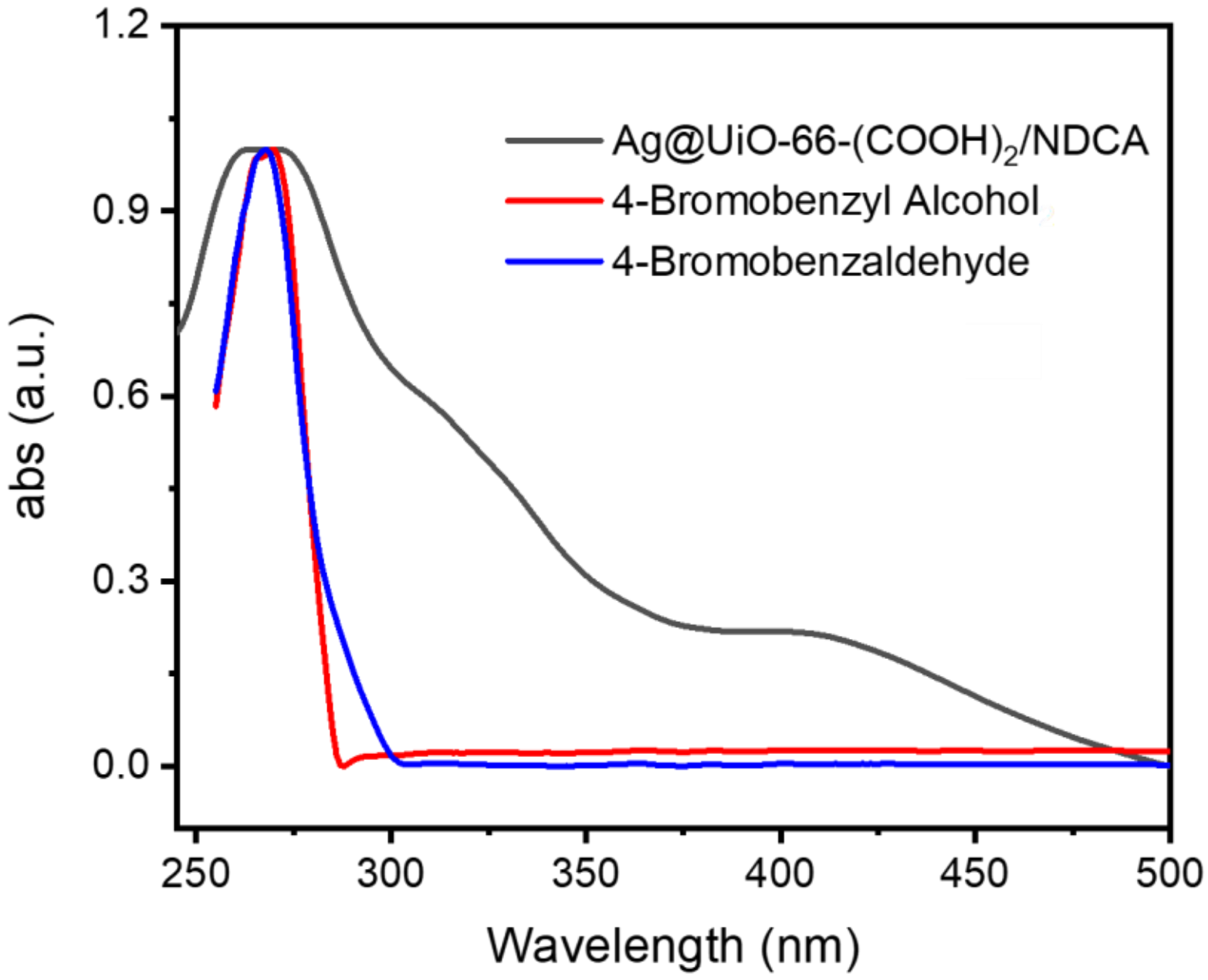

Figure S27. UV-vis spectra of Ag@UiO-66-$(COOH)_2$/NDCA, 4-bromobenzyl alcohol and 4-bromobenzaldehyde.

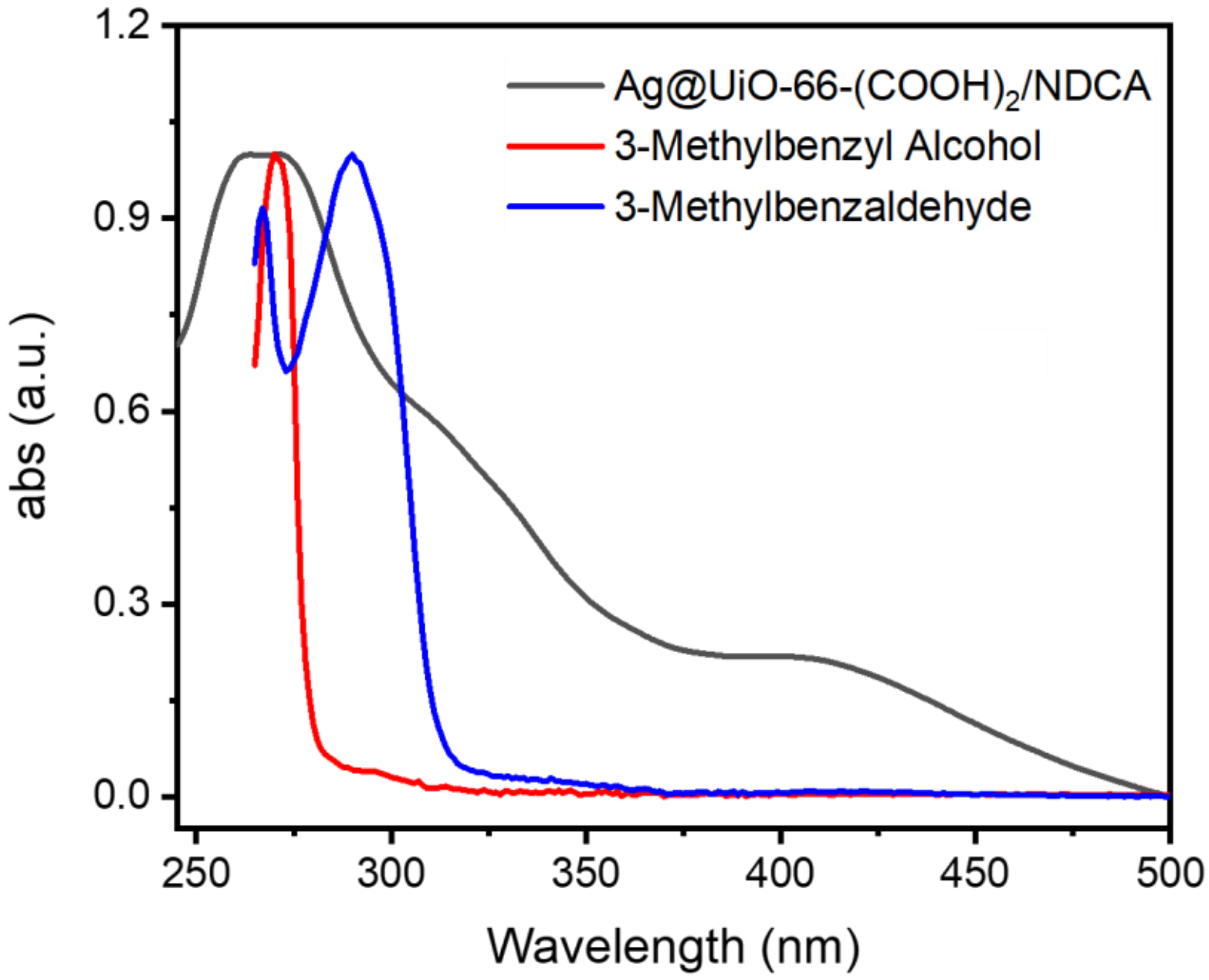

Figure S28. UV-vis spectra of Ag@UiO-66-$(COOH)_2$/NDCA, 3-methylbenzyl alcohol and 3-methylbenzaldehyde.

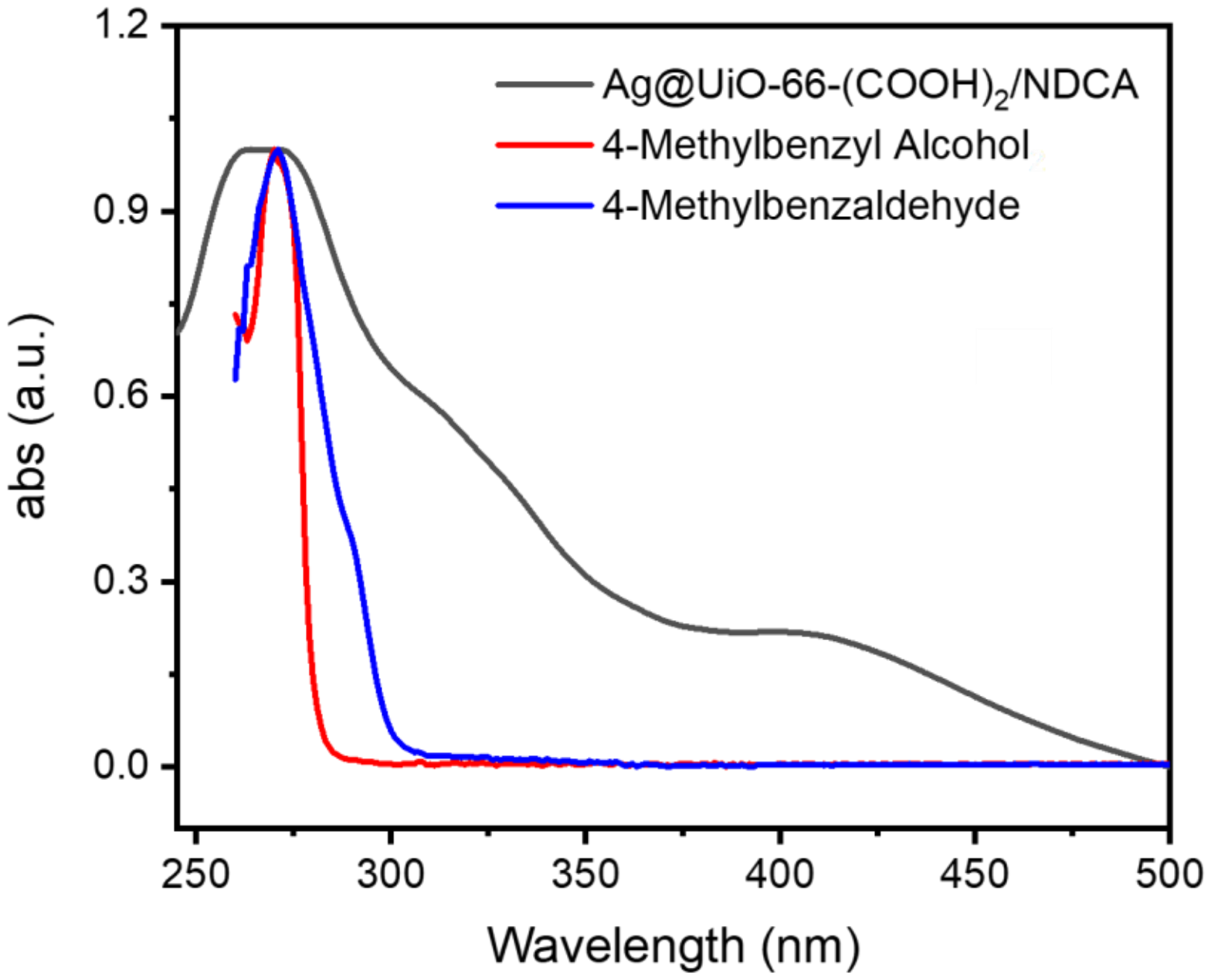

Figure S29. UV-vis spectra of Ag@UiO-66-$(COOH)_2$/NDCA, 4-methylbenzyl alcohol and 4-methylbenzaldehyde.

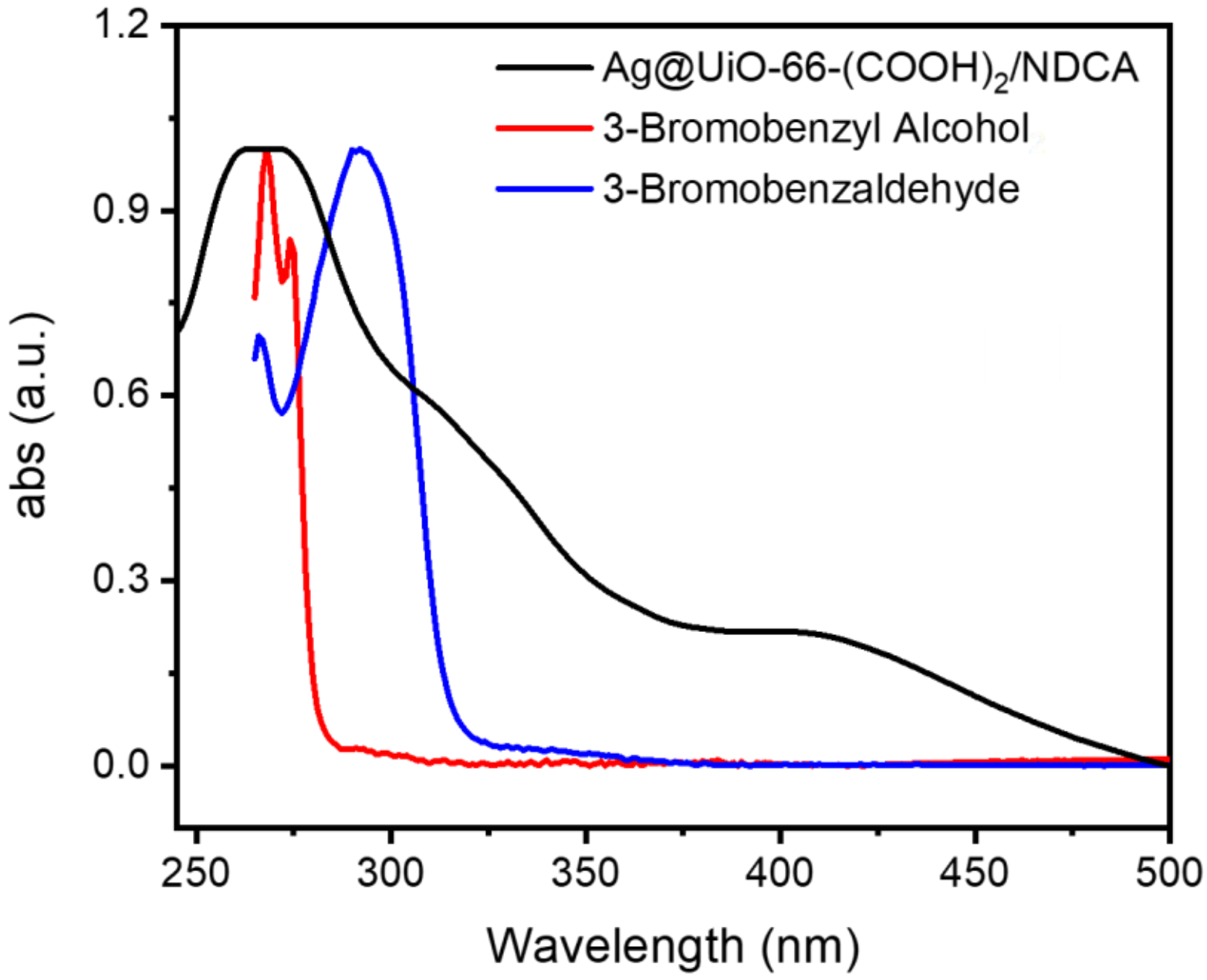

Figure S30. UV-vis spectra of Ag@UiO-66-$(COOH)_2$/NDCA, 3-bromobenzyl alcohol and 3-bromobenzaldehyde.

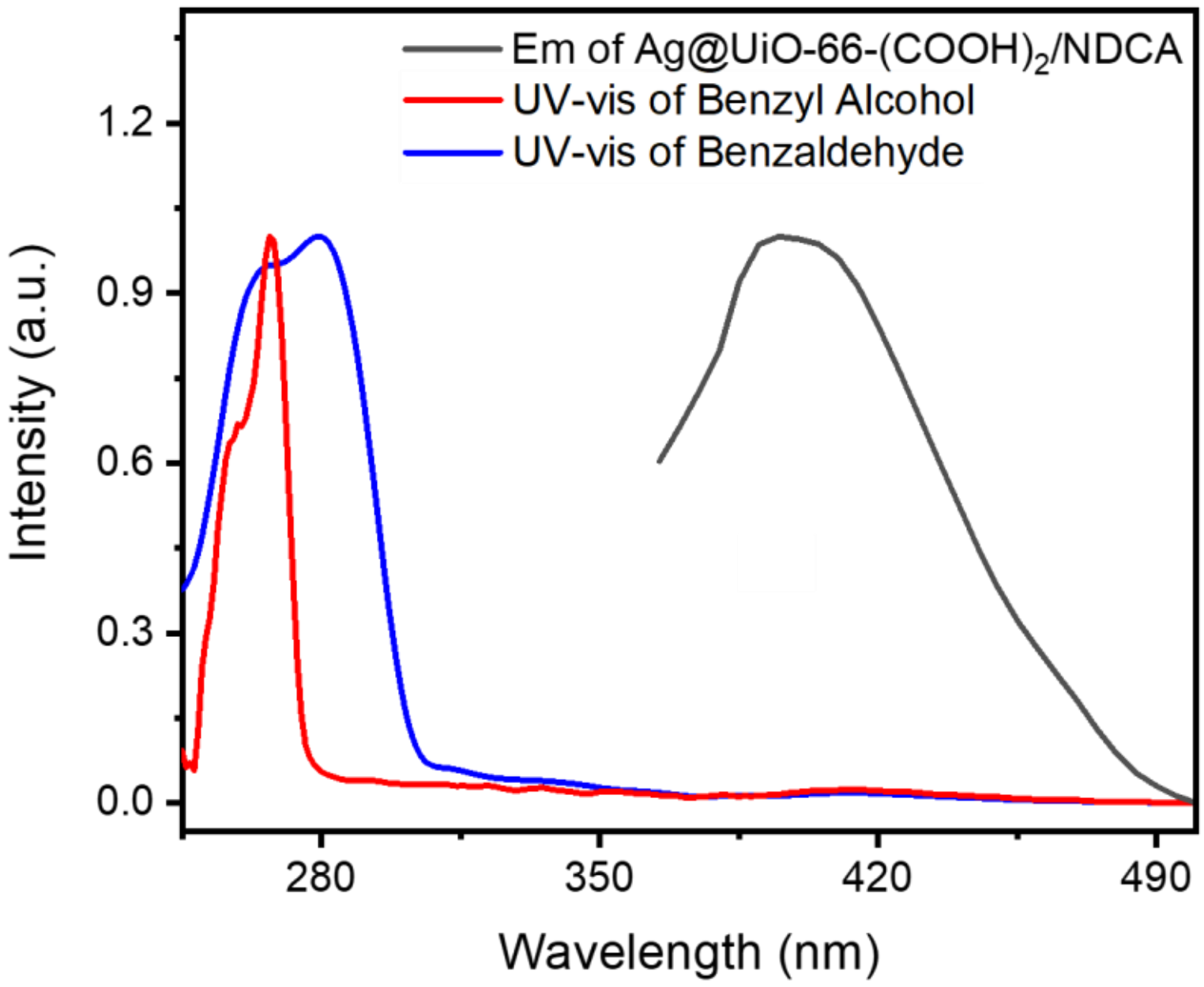

Figure S31. Emission spectrum of Ag@UiO-66-$(COOH)_2$/NDCA, and UV-vis spectra of benzyl alcohol and benzaldehyde.

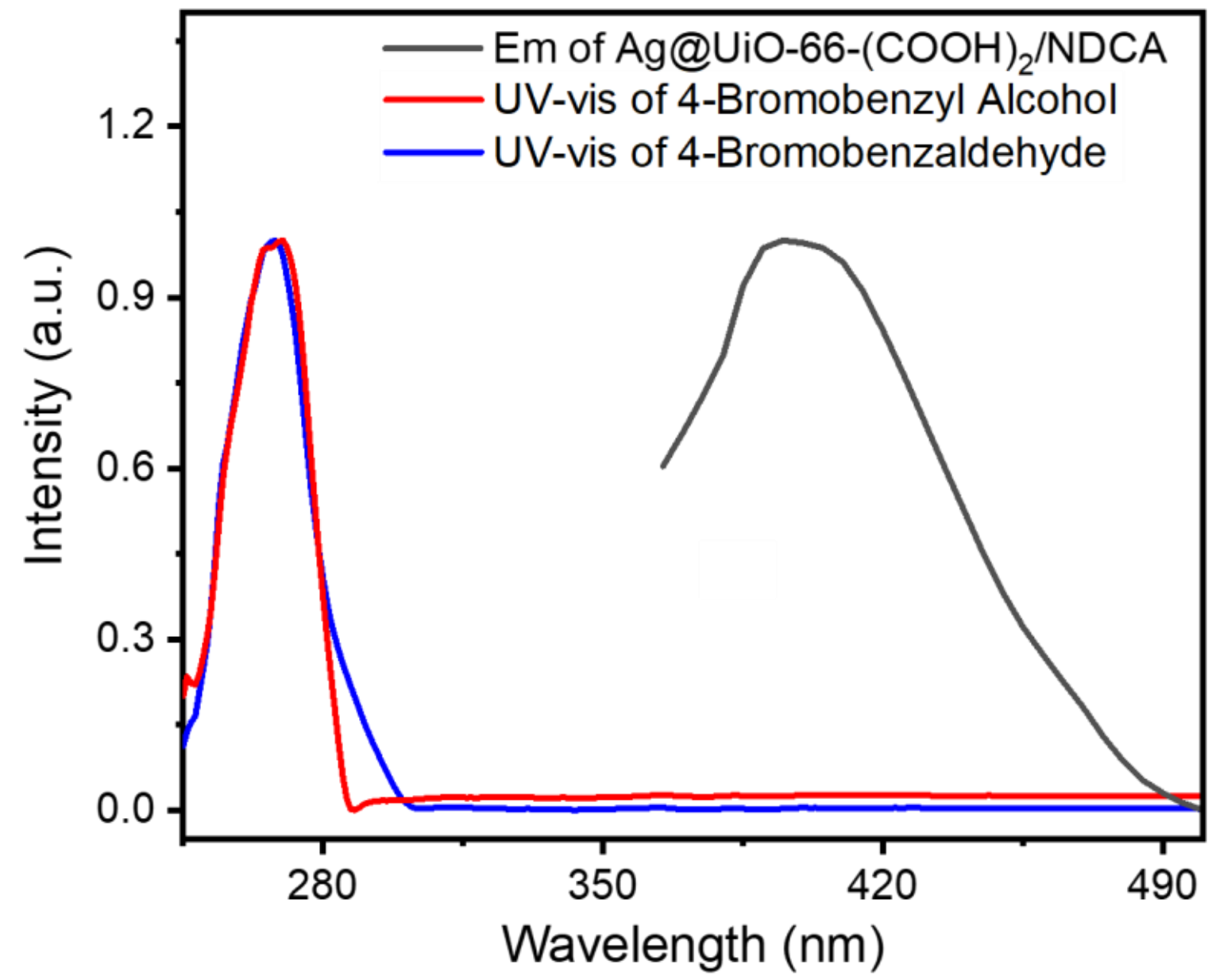

Figure S32. Emission spectrum of Ag@UiO-66-$(COOH)_2$/NDCA, and UV-vis spectra of 4-bromobenzyl alcohol and 4-bromobenzaldehyde.

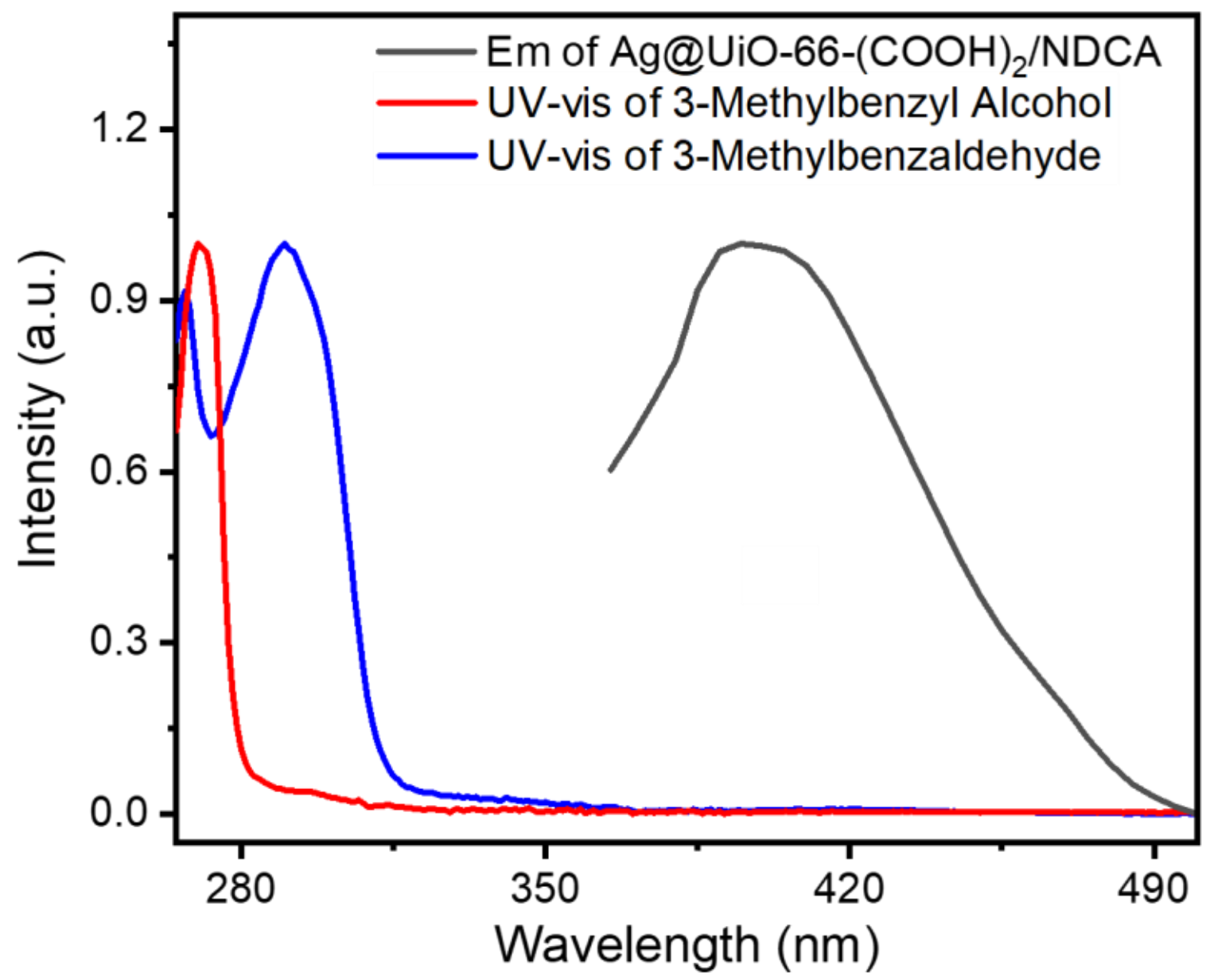

Figure S33. Emission spectrum of Ag@UiO-66-$(COOH)_2$/NDCA, and UV-vis spectra of 3-methylbenzyl alcohol and 3-methylbenzaldehyde.

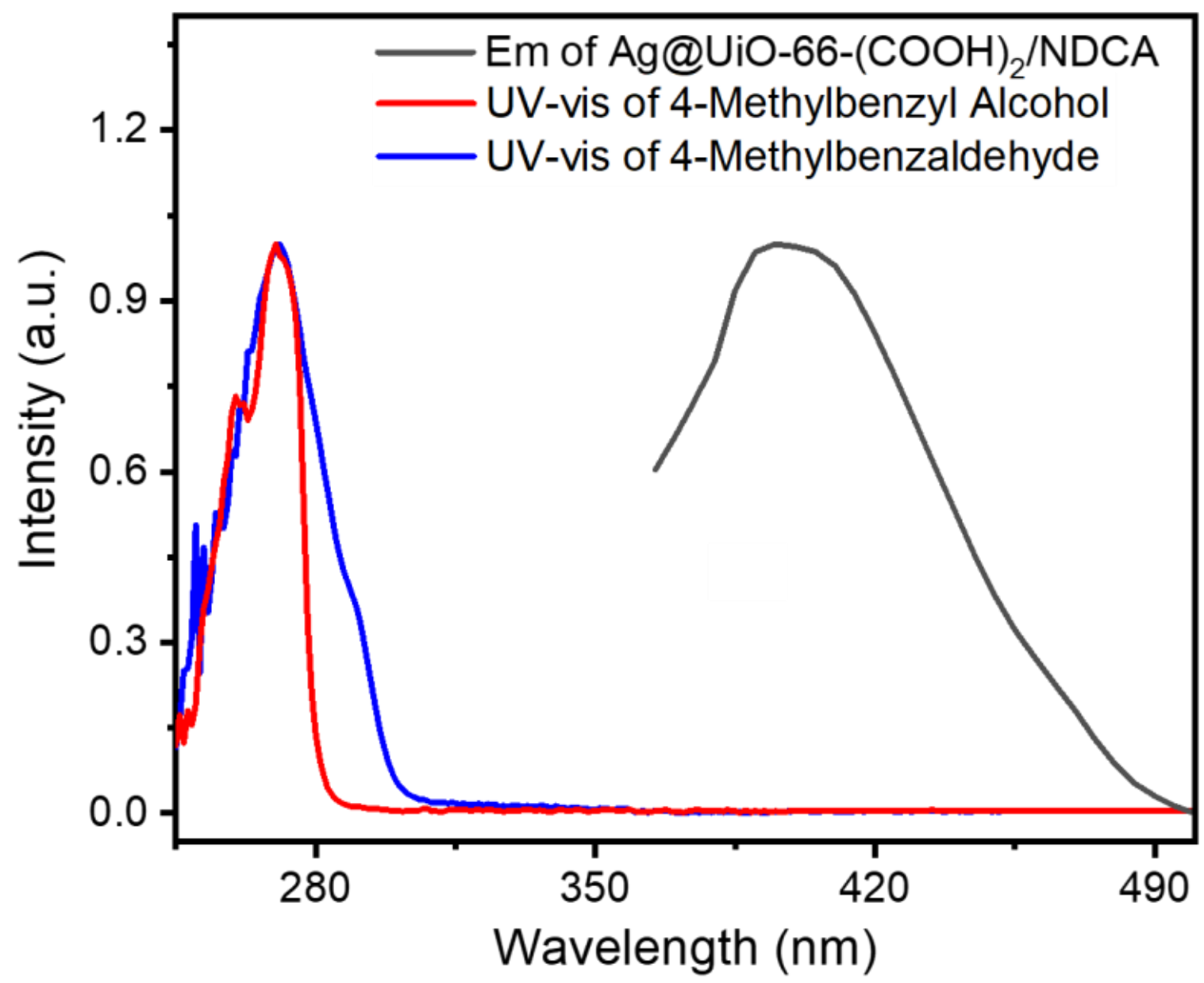

Figure S34. Emission spectrum of Ag@UiO-66-$(COOH)_2$/NDCA, and UV-vis spectra of 4-methylbenzyl alcohol and 4-methylbenzaldehyde.

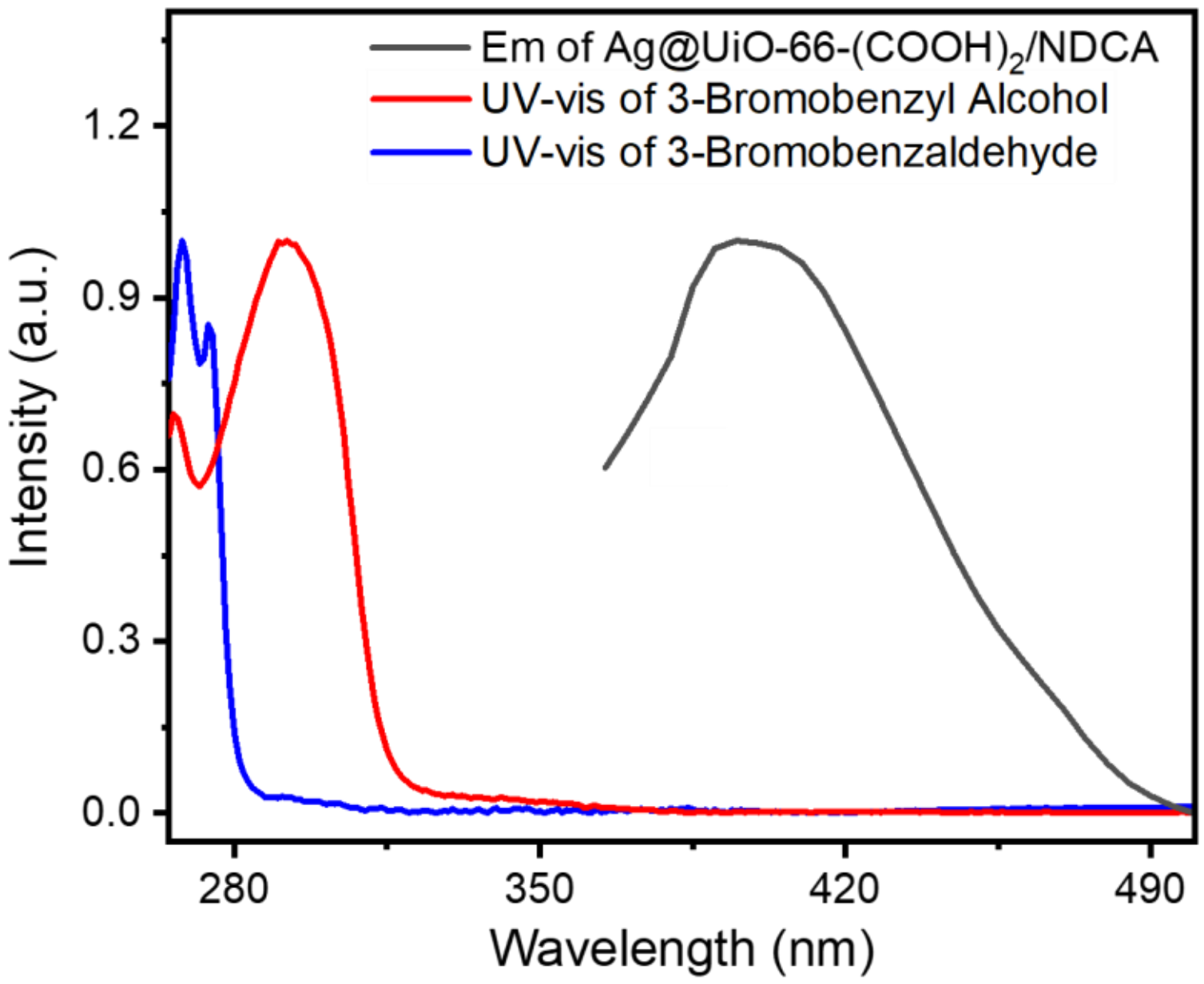

Figure S35. Emission spectrum of Ag@UiO-66-$(COOH)_2$/NDCA, and UV-vis spectra of 3-bromobenzyl alcohol and 3-bromobenzaldehyde.

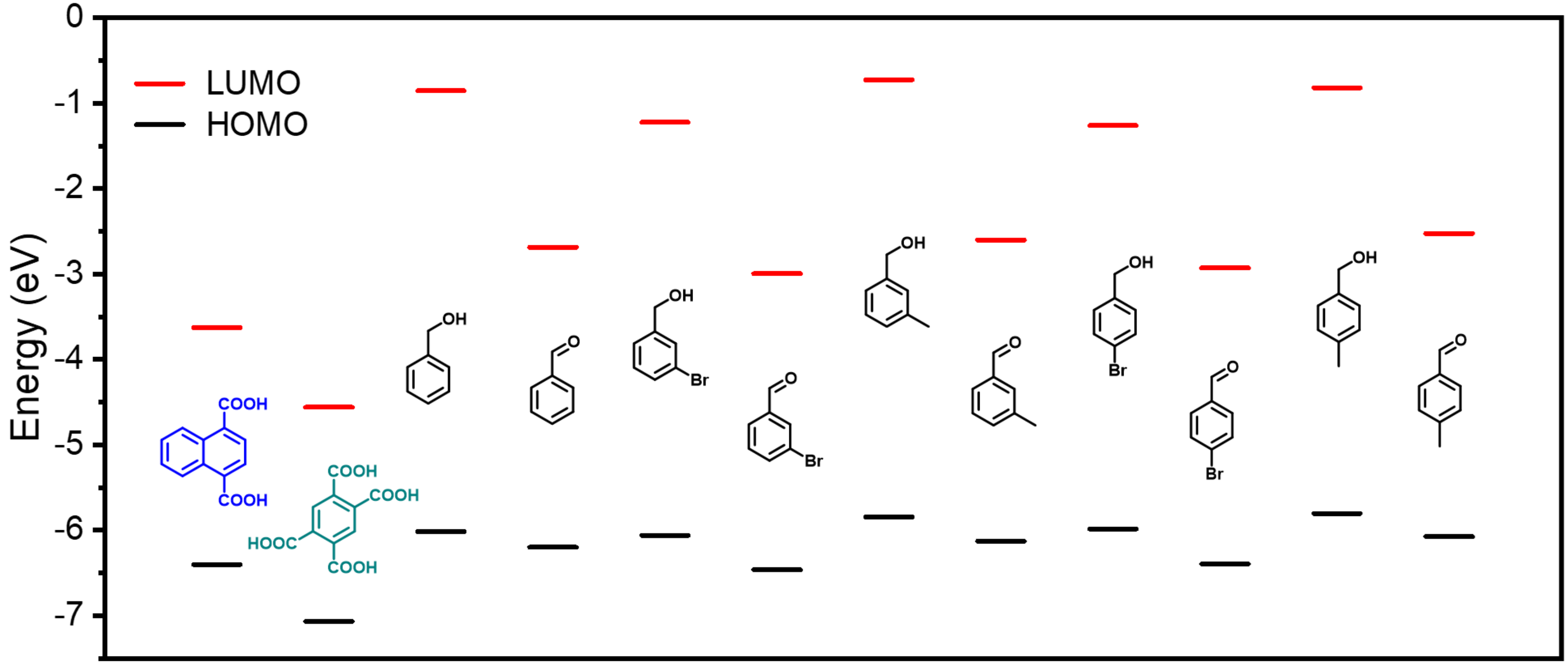

Figure S36. HOMO-LUMO energy levels of H_2NDCA, H_4PMA, all reactants and products.

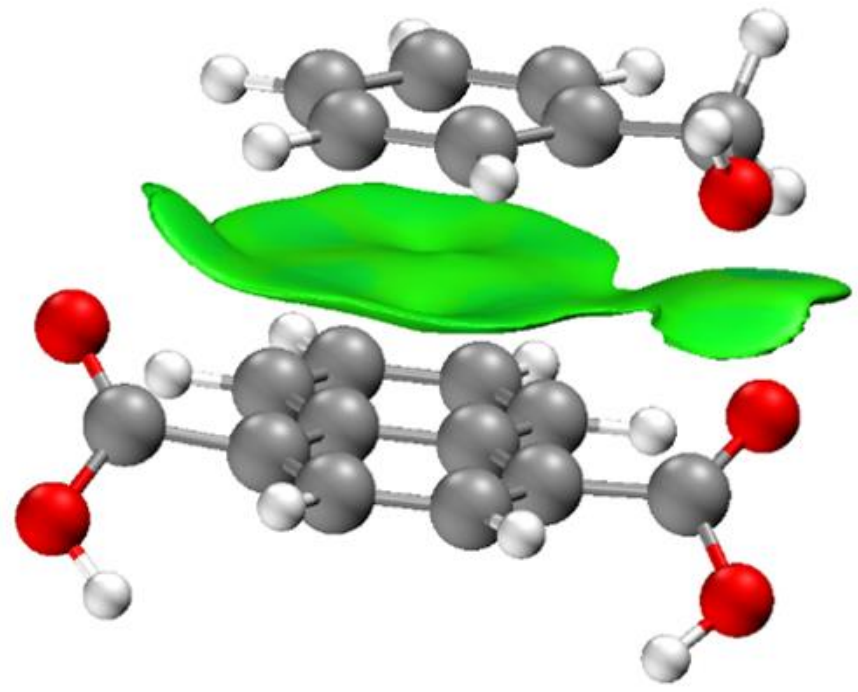

Figure S37. Dispersion interaction between H_2NDCA and benzyl alcohol. Atom code: C, gray; H, white; O, red. Green isosurfaces indicate the dispersion interaction region.

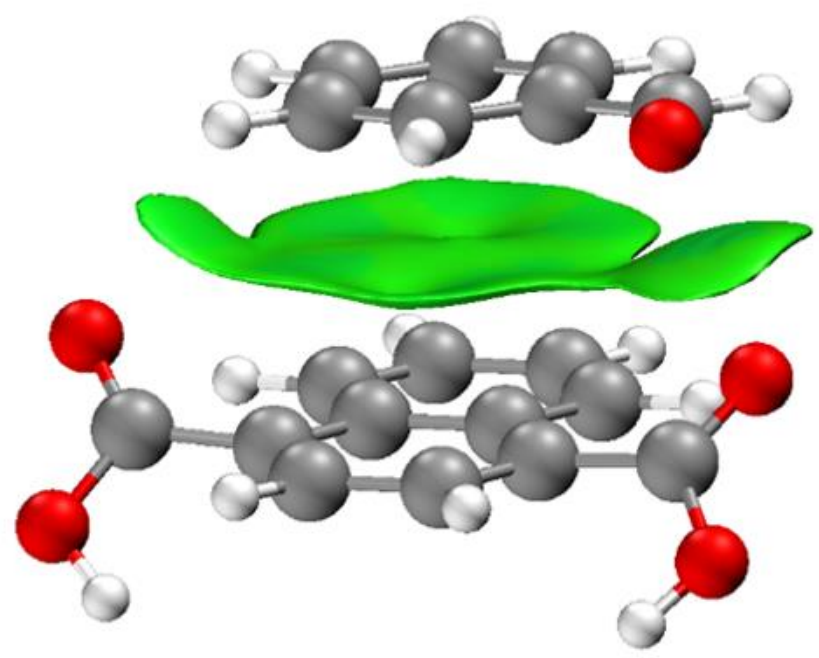

Figure S38. Dispersion interaction between H_2NDCA and benzyl aldehyde. Atom code: C, gray; H, white; O, red. Green isosurfaces indicate the dispersion interaction region.

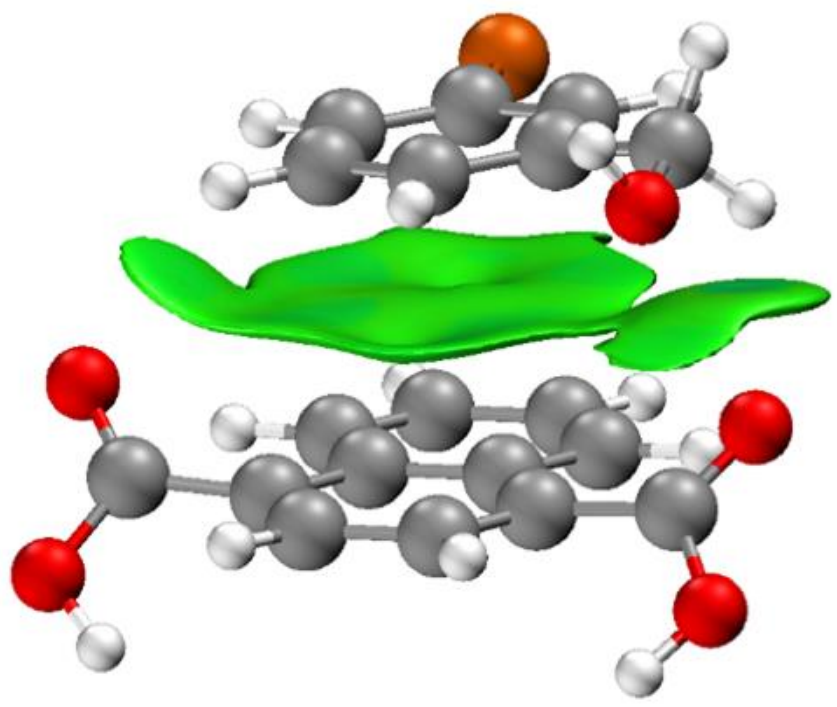

Figure S39. Dispersion interaction between H_2NDCA and 3-bromobenzyl alcohol. Atom code: C, gray; H, white; O, red; Br, orange. Green isosurfaces indicate the dispersion interaction region.

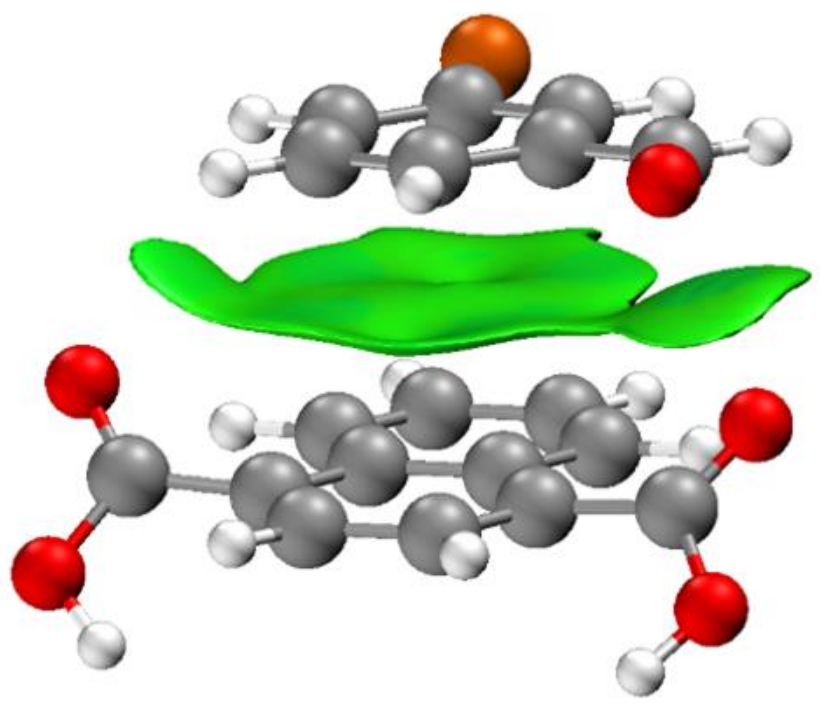

Figure S40. Dispersion interaction between H_2NDCA and 3-bromobenzaldehyde. Atom code: C, gray; H, white; O, red; Br, orange. Green isosurfaces indicate the dispersion interaction region.

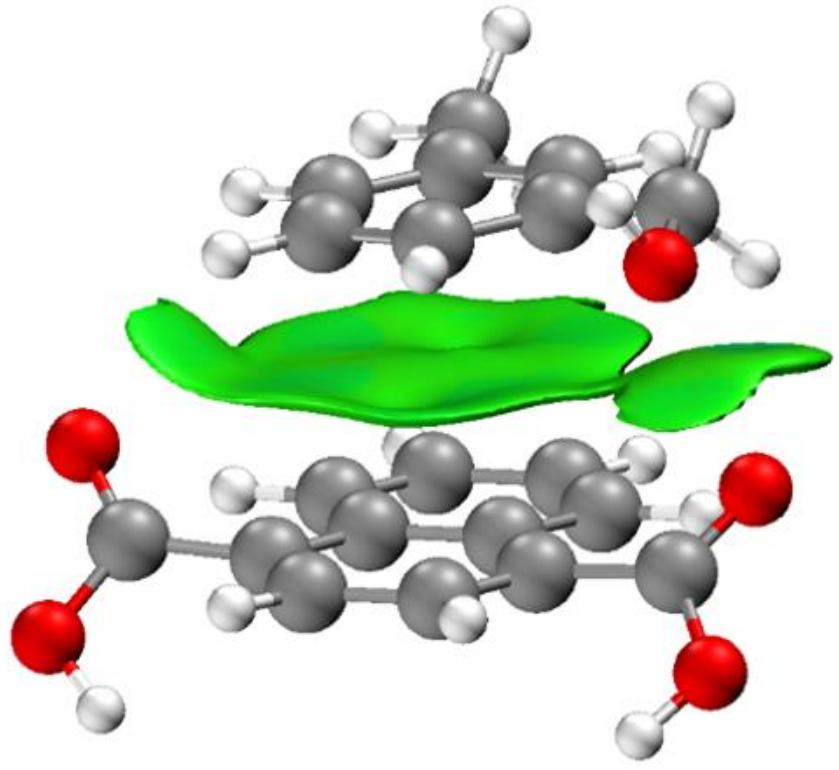

Figure S41. Dispersion interaction between H_2NDCA and 3-methylbenzyl alcohol. Atom code: C, gray; H, white; O, red. Green isosurfaces indicate the dispersion interaction region.

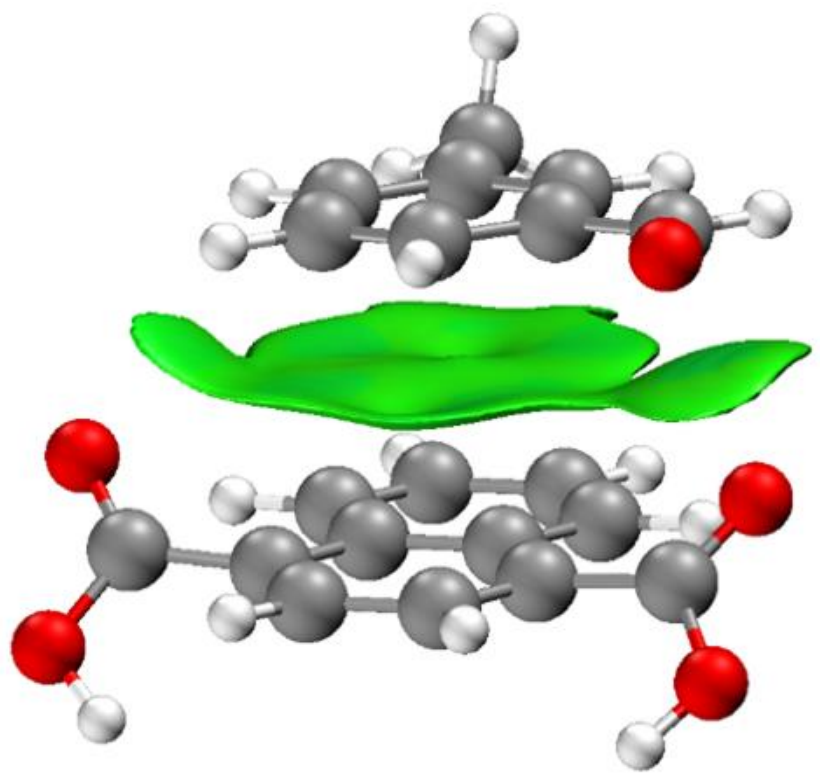

Figure S42. Dispersion interaction between H_2NDCA and 3-methylbenzaldehyde. Atom code: C, gray; H, white; O, red. Green isosurfaces indicate the dispersion interaction region.

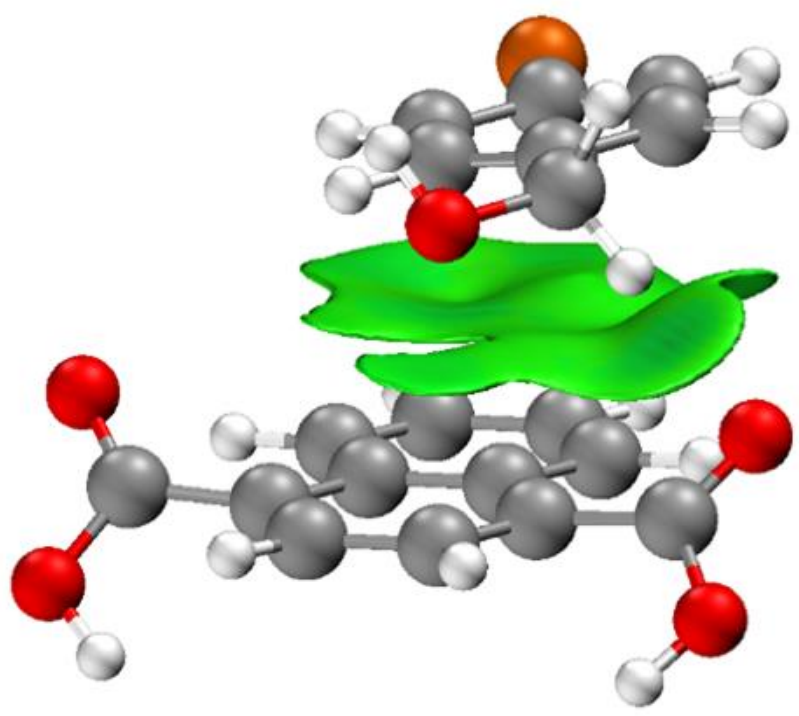

Figure S43. Dispersion interaction between H_2NDCA and 4-bromobenzyl alcohol. Atom code: C, gray; H, white; O, red; Br, orange. Green isosurfaces indicate the dispersion interaction region.

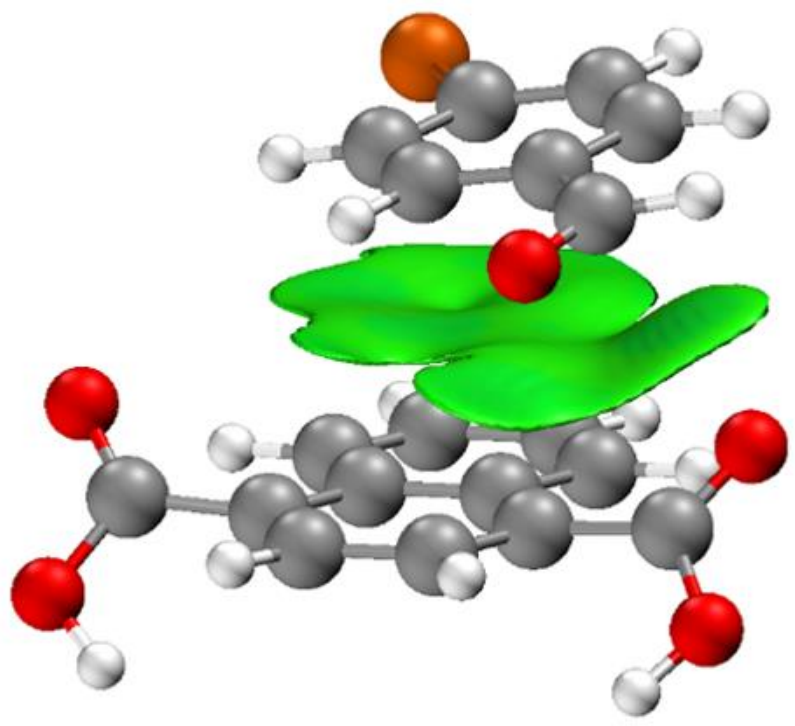

Figure S44. Dispersion interaction between H_2NDCA and 4-bromobenzaldehyde. Atom code: C, gray; H, white; O, red; Br, orange. Green isosurfaces indicate the dispersion interaction region.

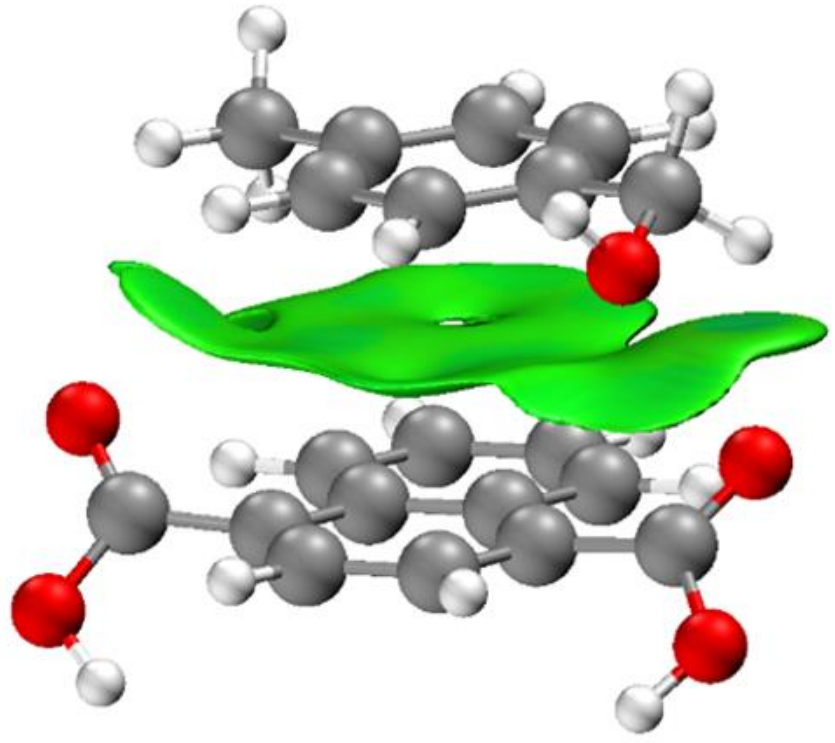

Figure S45. Dispersion interaction between H_2NDCA and 4-methylbenzyl alcohol. Atom code: C, gray; H, white; O, red. Green isosurfaces indicate the dispersion interaction region.

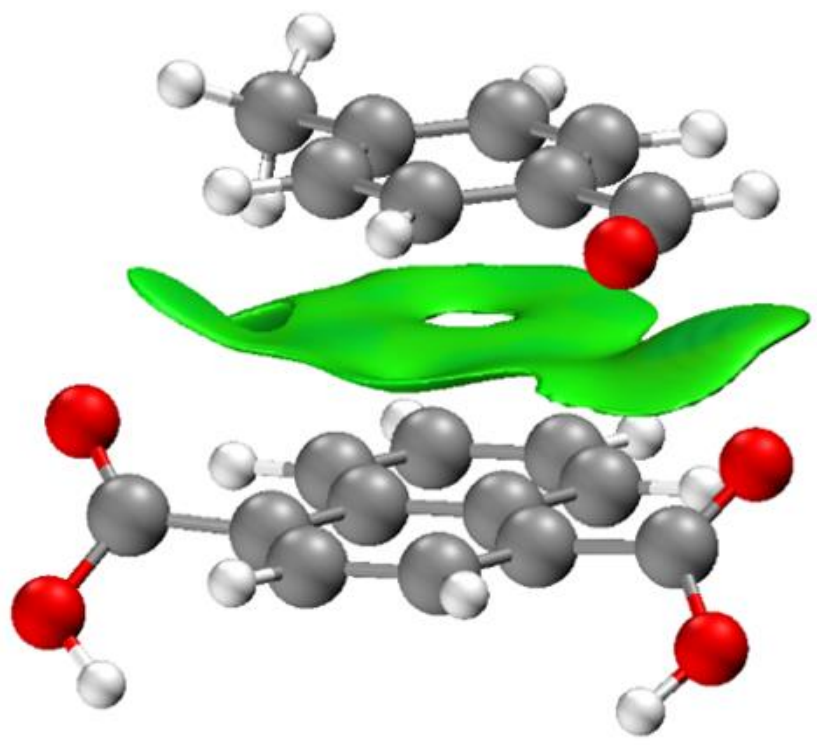

Figure S46. Dispersion interaction between H_2NDCA and 4-methylbenzaldehyde. Atom code: C, gray; H, white; O, red. Green isosurfaces indicate the dispersion interaction region.

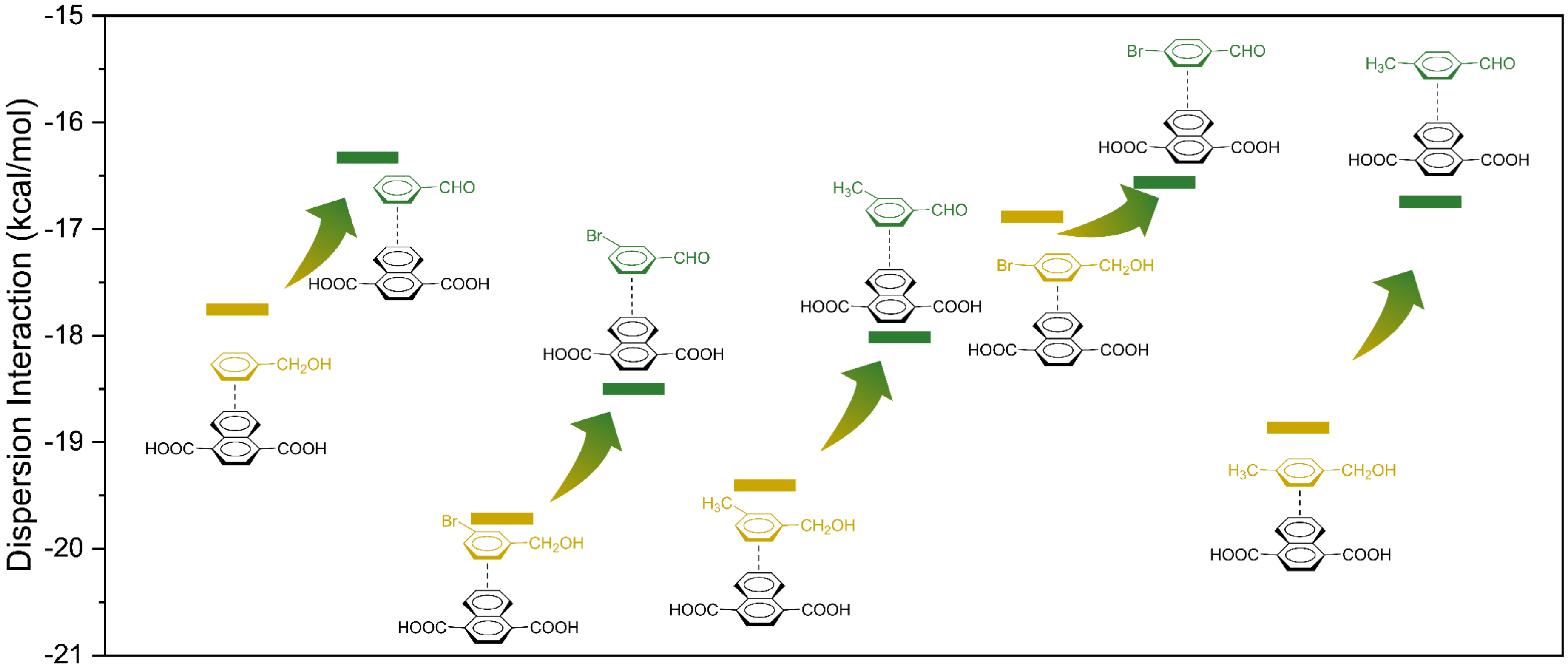

Figure S47. Dispersion interaction between H_2NDCA with reactants and products.

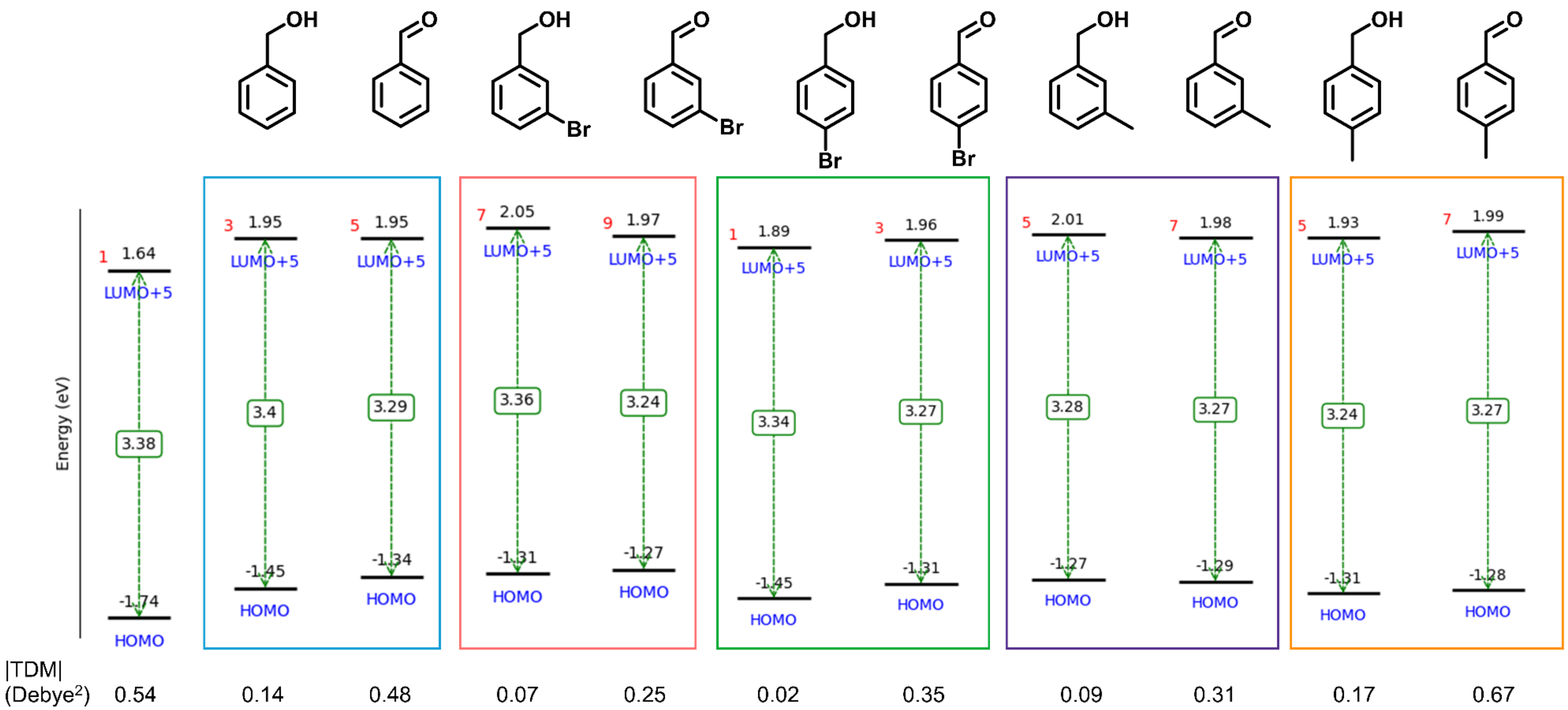

Figure S48. Transition dipole moments (TDM) of HOMO to LUMO+5 energy level around the adsorption energies. The first diagram shows the pristine UiO-66-$(COOH)_2$/NDCA structure, and the subsequent diagrams show the reactant/product pairs in the boxes. Blue: benzyl alcohol/benzyl aldehyde, red: 3-bromobenzyl alcohol/3-bromobenzaldehyde, green: 4-bromobenzyl alcohol/4-bromobenzaldehyde, violet: 3-methylbenzyl alcohol/3-methylbenzaldehyde, orange: 4-methylbenzyl alcohol/4-methylbenzaldehyde.

Tables

Table S1. Crystallographic data and structure refinement details.

	$Ag@UiO\text{-}66\text{-}(COOH)_2$
CCDC	2499997
Formula	$C_{50.4}Ag_{0.3}O_{38}Zr_6$
Formula wt	1792.99
Temperature (K)	110.00(10)
Crystal System	cubic
Space Group	*Fm*-3*m*
a (Å)	20.76120(10)
b (Å)	20.76120(10)
c (Å)	20.76120(10)
α (deg)	90
β (deg)	90
γ (deg)	90
Z	4
V (Å^3)	8948.65(13)
ρ_{calc} (g cm^{-3})	1.331
μ (mm^{-1})	6.676
F (000)	3442.0
R_{int}	0.0366
$^aR_1$, $^bwR_2$ ($I > 2\sigma(I)$)	0.0492, 0.1409
$^aR_1$, $^bwR_2$ (all data)	0.0511, 0.1413

$^aR_1 = \Sigma||F_o| - |F_c||/\Sigma|F_o|$, $^bwR_2 = [\Sigma w(F_o^2 - F_c^2)^2/\Sigma w(F_o^2)^2]^{1/2}$

Table S2. Progress of the reaction **1** confirmed by ^{1}H NMR at different time.

**Time (min)**	**Reactant : Product**	**Yield (%)**
15	1.43	41.18
20	0.81	55.36
25	0.70	58.68
30	0.63	61.54
40	0.59	62.96
50	0.60	62.41
60	0.60	62.69
70	0.57	63.77
80	0.60	62.69
90	0.58	63.24
100	0.58	63.24
110	0.60	62.41
120	0.60	62.41

Table S3. Progress of the reaction **2** confirmed by ^{1}H NMR at different time.

Time (min)	Reactant : Product	Yield (%)
15	0.74	57.63
20	0.57	63.77
25	0.56	64.29
30	0.56	64.29
40	0.55	64.54
50	0.54	65.03
60	0.56	64.03
70	0.56	64.03
80	0.55	64.54
90	0.56	64.29
100	0.54	64.79
110	0.56	64.29
120	0.56	64.29

Table S4. Progress of the reaction **3** confirmed by ^{1}H NMR at different time.

Time (min)	Reactant : Product	Yield (%)
15	1.22	45.05
20	0.98	50.50
25	0.75	57.26
30	0.78	56.14
40	0.79	55.75
50	0.81	55.36
60	0.77	56.52
70	0.76	56.90
90	0.81	55.36
100	0.78	56.14
110	0.74	57.63
120	0.78	56.14

Table S5. Progress of the reaction **4** confirmed by ^{1}H NMR at different time.

Time (min)	Reactant : Product	Yield (%)
15	3.85	20.63
20	1.09	47.92
25	0.93	51.92
30	0.81	55.36
40	0.81	55.36
50	0.82	54.95
60	0.85	54.13
70	0.83	54.55
80	0.86	53.70
90	0.85	54.13
100	0.78	56.14
110	0.89	52.83
120	0.86	53.70

Table S6. Progress of the reaction **5** confirmed by ^{1}H NMR at different time.

Time (min)	Reactant : Product	Yield (%)
15	1.61	38.27
20	0.93	51.92
25	0.68	59.68
30	0.63	61.54
40	0.60	62.41
50	0.63	61.54
60	0.60	62.69
70	0.59	62.96
80	0.68	59.68
90	0.63	61.54
100	0.71	58.33
110	0.63	61.24
120	0.60	62.41

References

1. Hu, Z.; Peng, Y.; Kang, Z.; Qian, Y.; Zhao, D. A modulated hydrothermal (MHT) approach for the facile synthesis of UiO-66-type MOFs. *Inorg. Chem.* **54**, 4862-4868 (2015).

2. Neese, F., Wennmohs, F., Becker, U. & Riplinger, C. The ORCA quantum chemistry program package. *J. Chem. Phys.* **152**, 224108 (2020).

3. Neese, F. Software update: The ORCA program system—Version 5.0. *WIREs Comput. Mol. Sci.* **12**, e1606 (2022).

4. Grimme, S., Hansen, A., Ehlert, S. & Mewes, J.-M. r^2SCAN-3c: A "Swiss army knife" composite electronic-structure method. *J. Chem. Phys.* **154**, 064103 (2021).

5. Kruse, H. & Grimme, S. A geometrical correction for the inter- and intra-molecular basis set superposition error in Hartree-Fock and density functional theory calculations for large systems. *J. Chem. Phys.* **136**, 154101 (2012).

6. Caldeweyher, E., Bannwarth, C. & Grimme, S. Extension of the D3 dispersion coefficient model. *J. Chem. Phys.* **147**, 034112 (2017).

7. Caldeweyher, E. *et al*. A generally applicable atomic-charge dependent London dispersion correction. *J. Chem. Phys.* **150**, 154122 (2019).

8. Szalewicz, K. Symmetry-adapted perturbation theory of intermolecular forces. *WIREs Comput. Mol. Sci.* **2**, 254-272 (2012).

9. Smith, D. G. A. *et al*. PSI4 1.4: Open-source software for high-throughput quantum chemistry. *J. Chem. Phys*. **152**, 184108 (2020).

10. Lu, T. & Chen, Q. Independent gradient model based on Hirshfeld partition: A new

method for visual study of interactions in chemical systems. *J. Comput. Chem.* **43**, 539-555 (2022).

11. Lu, T. & Chen, F. Multiwfn: A multifunctional wavefunction analyzer. *J. Comput. Chem.* **33**, 580-592 (2012).

12. Lu, T. A comprehensive electron wavefunction analysis toolbox for chemists, Multiwfn. *J. Chem. Phys.* **161**, 082503 (2024).

13. Humphrey, W., Dalke, A. & Schulten, K. VMD: Visual molecular dynamics. *J. Mol. Graph.* **14**, 33-38 (1996).

14. Kresse, G. & Hafner, J. Ab initio molecular dynamics for liquid metals. *Phys. Rev. B* **47**, 558-561 (1993).

15. Kresse, G. & Furthmüller, J. Efficiency of ab-initio total energy calculations for metals and semiconductors using a plane-wave basis set. *Comput. Mater. Sci.* **6**, 15-50 (1996).

16. Kresse, G. & Furthmüller, J. Efficient iterative schemes for ab initio total-energy calculations using a plane-wave basis set. *Phys. Rev. B* **54**, 11169-11186 (1996).

17. Kresse, G. & Joubert, D. From ultrasoft pseudopotentials to the projector augmented-wave method. *Phys. Rev. B* **59**, 1758-1775 (1999).

18. Perdew, J. P., Burke, K. & Ernzerhof, M. Generalized gradient approximation made simple. *Phys. Rev. Lett.* **77**, 3865-3868 (1996).

19. Grimme, S., Ehrlich, S. & Goerigk, L. Effect of the damping function in dispersion corrected density functional theory. *J. Comput. Chem.* **32**, 1456-1465 (2011).

20. Krukau, A. V., Vydrov, O. A., Izmaylov, A. F. & Scuseria, G. E. Influence of the

exchange screening parameter on the performance of screened hybrid functionals. *J. Chem. Phys.* **125**, 224106 (2006).

21. Wang, V., Xu, N., Liu, J.-C., Tang, G. & Geng, W.-T. VASPKIT: A user-friendly interface facilitating high-throughput computing and analysis using VASP code. *Comput. Phys. Commun.* **267**, 108033 (2021).

22. Macrae, C. F. *et al*. Mercury 4.0: from visualization to analysis, design and prediction. *J. Appl. Crystallogr.* **53**, 226-235 (2020).
